# Key Interventions in Friedreich's Ataxia and Their Impact on Patient Outcomes: A Systematic Review

**DOI:** 10.1002/mds.70423

**Published:** 2026-07-06

**Authors:** Dorota Sarwinska, Maresa Buchholz, Audrey Iskandar, Marcus Grobe‐Einsler, Thomas Klockgether, Jennifer Faber, Bernhard Michalowsky

**Affiliations:** ^1^ German Center for Neurodegenerative Diseases e.V., Site Rostock/Greifswald Greifswald Germany; ^2^ German Center for Neurodegenerative Diseases e.V., Site Bonn Bonn Germany; ^3^ Center for Neurology, Department of Parkinson's Disease, Sleep and Movement Disorders University Hospital Bonn Bonn Germany; ^4^ Department of Neurology Center for Movement Disorders and Neuromodulation, Medical Faculty, Heinrich‐Heine University Düsseldorf Germany; ^5^ Department of Neuroradiology University Hospital Bonn Bonn Germany; ^6^ McMaster University, Health Research Methods, Evidence and Impact Hamilton Ontario Canada

**Keywords:** Friedreich's ataxia, intervention, rehabilitation, pharmacotherapy, patient‐reported outcomes, quality of life

## Abstract

Friedreich's ataxia (FA) is a rare neurodegenerative disease with multisystemic symptoms that requires multidisciplinary care. This systematic review summarizes available pharmacological and nonpharmacological interventions, their outcomes, and alignment with patient‐centered care domains, as well as their impact on these domains. The publication search was conducted in three databases (PubMed, Embase, Cochrane Library) from 2010 to 2025 for human studies. Studies were included if they met predefined eligibility criteria (FA population/pharmacological or nonpharmacological interventions/use of comparator/any outcomes, especially patient reported). All study designs were included. Risk of bias assessment was performed by two reviewers using Cochrane RoB‐2 for randomized controlled trials and JBI Critical Appraisal Tools for case reports, case series, and quasi‐experimental studies. Results were synthesized narratively. Ninety studies (69 FA only, 21 mixed ataxia) were included. FA‐only studies included randomized controlled trials (32%), quasi‐experimental studies (35%), and case reports (33%), mostly with small samples (<100) and low‐to‐moderate bias. Pharmacological interventions (66.7%), mainly disease modifying (93.5%), were more common than nonpharmacological interventions, with the most effective interventions being omaveloxolone (pharmacological), as well as rehabilitation and heart interventions (nonpharmacological). Pharmacological studies often showed biological improvements without clear clinical benefit, whereas nonpharmacological therapies yielded more consistent patient‐relevant gains, although evidence was limited. Physical health was the most frequently addressed domain (42.3%), whereas mental health and caregiver aspects were rarely studied (1.8% and 1.4%). No study investigated caregiver burden or economic outcomes. More holistic studies are needed, integrating patient‐ and caregiver‐reported outcomes and combining multiple treatment approaches in larger cohorts. © 2026 The Author(s). *Movement Disorders* published by Wiley Periodicals LLC on behalf of International Parkinson and Movement Disorder Society.

Friedreich's ataxia (FA) is a rare neurodegenerative genetic disorder with a prevalence between 1:200,000 and 1:750,000 in Europe^1^; it is the most common autosomal recessive ataxia, most frequently caused by a GAA trinucleotide repeat expansion in the frataxin (FXN) gene. The expanded GAA ultimately leads to decreased frataxin protein levels, and thus to iron accumulation in mitochondria, increased oxidative stress, and cell degeneration.^2^, ^3^ Depending on the length of GAA1 repetitions, the disease onset can occur either earlier in childhood (longer expansion) or later in adolescence (shorter expansion).^4^ The age at onset is also inversely correlated with disease severity, where faster progression and earlier loss of ambulation are observed in patients with early onset.^4^, ^5^, ^6^

FA is a multisystemic, progressive disease with neurological core symptoms, including ataxia, dysarthria, sensory loss, muscle weakness, and spasticity, as well as nonneurological symptoms such as cardiomyopathy, diabetes mellitus, scoliosis, and pes cavus.^2^, ^7^, ^8^, ^9^, ^10^, ^11^ Mental disorders and decline in cognitive functions also occur in the course of the disease.^12^, ^13^, ^14^ Caregivers of patients with FA also report worries and uncertainty about their loved ones, as well as managing their care, representing a substantial caregiver burden.^15^ All mentioned symptoms, burdens, and challenges notably influence patients' and caregivers' quality of life.^15^, ^16^

FA requires complex treatment, care, and support. Therefore, several pharmacological and nonpharmacological approaches exist,^2^, ^17^ with a primary focus on physical health and clinical outcomes. Since 2024, omaveloxolone has been approved in both the United States and the European Union as the first disease‐modifying treatment for FA,^18^, ^19^ representing an important milestone.^20^, ^21^

Due to the variety of symptoms and available interventions, a summary of all available evidence about existing interventions in FA would help identify the possible range of treatment options. Despite this, little is known about how the interventions affected various health domains, including physical, mental, and psychosocial health, which is summarized as health‐related quality of life. Therefore, it is essential to comprehensively capture all existing evidence on interventional approaches in FA to identify key intervention categories and evaluate their impact on patient‐ and caregiver‐related outcomes. However, so far, no systematic review has synthesized the currently available interventions.

Consequently, this study aims to systematically review all available evidence on pharmacological and nonpharmacological interventions implemented in FA, with the objectives of (1) identifying key intervention categories employed in FA, (2) assessing their alignment with core patient and caregiver outcome domains, and (3) evaluating the intervention impact on these outcomes. Understanding these effects is crucial for developing holistic, patient‐ and caregiver‐centered treatment and care strategies in FA.

## Methods

This systematic review was performed in accordance with the *Cochrane Handbook for Systematic Reviews of Interventions*.^22^ The PRISMA 2020 checklist was submitted for editorial review.^23^

### Protocol and Registration

The protocol for this review was registered in PROSPERO (ID No. CRD420251075597). All predefined steps were followed throughout the whole process to ensure transparency and quality of this review.^24^

### Deviations from the Protocol

After registration, two methodological modifications were implemented: the risk of bias tool was changed to better match the study designs, and the planned certainty assessment was not conducted because of substantial heterogeneity of the evidence. All deviations were documented to maintain transparency.

### Study Eligibility Criteria

Study eligibility criteria were determined based on the PICOS (Population, Intervention, Comparison, Outcome, Study design) approach for systematic reviews.^22^ Inclusion and exclusion criteria are presented in Table S1.

### Information Sources and Search Strategy

Three databases were searched: PubMed, Embase, and Cochrane Library (CENTRAL). The pilot search was conducted before the study protocol registration. The search included publications released since 2010, with no language limitation applied, as long as an English abstract was available. Texts from foreign languages were translated into English. Animal, cell, or in silico studies were excluded.

The search strategy was based on the two dimensions: (1) “Friedreich ataxia” and (2) “interventions.” The key words used in the first dimension included “Friedreich ataxia” (MeSH), “cerebellar ataxia,” “hereditary ataxia,” and “progressive ataxia.” Because FA is a rare disease, the search also included other ataxias because studies often group several ataxia types together. In the second dimension, key words encompassed a variety of interventions that included both pharmacological and nonpharmacological aspects, such as “intervention,” “rehabilitation” [MeSH], “treatment” [MeSH], “training,” “exercise,” and “therapy.”

The search excluded reviews to limit the options for manual screening. Potential eligible publications identified in previous reviews, but not captured by the search strategy, were manually added. In addition, gray literature was searched through Google Scholar. The searching was finalized on June 3, 2025, for databases and on June 18, 2025, for manual search. The exact search strategy can be found in Table S2.

### Study Selection

Publications from the databases search, manual search, and previous reviews were then added to the online tool Rayyan. Duplicates were removed, and both initial (Title/Abstract) and full‐text screenings were performed^25^ by two reviewers (D.S. and M.B.) against the predefined inclusion and exclusion criteria. Discrepancies were discussed and solved involving the third reviewer (B.M.).

### Data Extraction

From the included publications, we extracted data for (1) study identification (author, year of publication, country), (2) characteristics (design), (3) population (sample size, age, FA characteristics), (4) intervention (type, dose, administration, duration), and (5) outcomes (types, domains, categories, outcome measure, result, observation period).

### Risk of Bias Assessment

On the basis of the designs of the included studies, we used the Cochrane RoB‐2 for randomized controlled trials (RCTs),^26^ the JBI Critical Appraisal Tool for quasi‐experimental studies,^27^ and the JBI Critical Appraisal Tool for case reports and case series^28^ for risk of bias assessment, which was performed independently by two reviewers (D.S. and M.B.). In case of discrepancies, the third reviewer (B.M.) was consulted. The results were standardized and presented in a scale in a color‐coded manner: ≥75% low risk of bias (green), 50% to 75% moderate (yellow), ≤50% high (red).^29^ Because the study design directly influences the general bias of the study, the assessed risk of bias should be considered within a study design, rather than comparing studies with different designs.

### Data Synthesis

Due to the heterogeneity of interventions, a narrative synthesis of the gathered outcomes was performed. The interventions were grouped into key categories, and their effects on the outcomes were compared descriptively. In addition, the alignment of the interventions with core outcome domains defined by the International Consortium for Health Outcomes Measurement (ICHOM) (including physical functioning, symptom control, quality of life, mental well‐being, and caregiver burden) was assessed.^23^ FA affects both children and adults and, like dementia, is a progressive neurodegenerative disease without a cure, requiring increasing medical and informal care over time. Therefore, the existing ICHOM outcome sets for general pediatric health, general adult health, and dementia were used to assess intervention outcome alignment with health domains.

## Results

### Study Selection

The search yielded 2227 studies, from which 114 were removed as duplicates. After title and abstract screening, 136 publications were eligible for full‐text screening. Finally, 90 publications (69 FA only and 21 mixed ataxia population; Fig. 1A) were included in the narrative syntheses. The study selection process is presented on the PRISMA chart in Figure S1.^30^

**FIG. 1:**
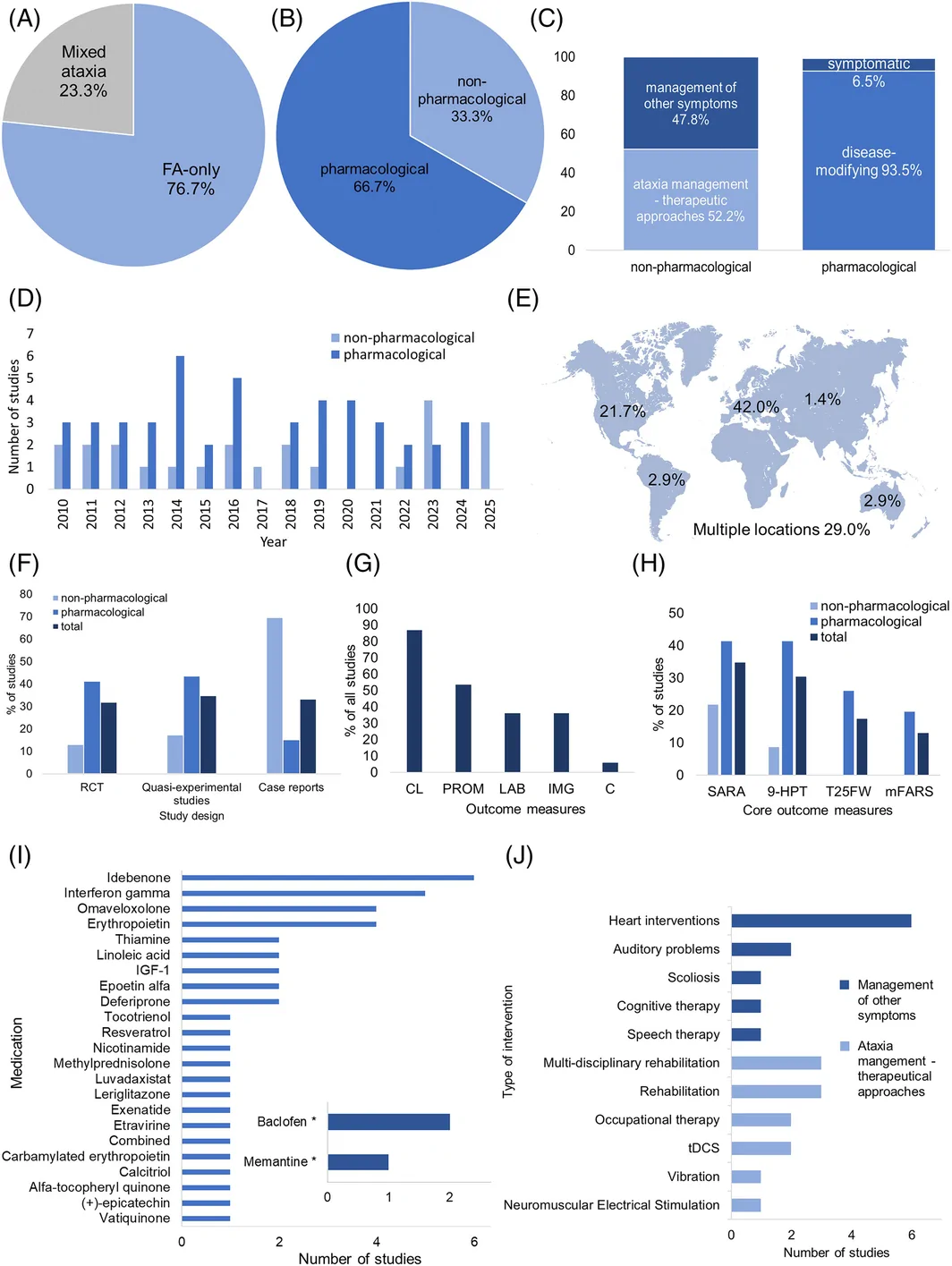
Characteristics of included studies. (**A**) Study populations: n = 69 studies for patients with Friedreich's ataxia (FA) only and n = 21 for patients with mixed ataxia. (**B**) Interventions types: n = 46 pharmacological interventions (P), n = 23 nonpharmacological interventions (NP). (**C**) Further division of nonpharmacological interventions. (**D**) Timeline for pharmacological and nonpharmacological studies. (**E**) Geographic location of included studies. (**F**) Study designs of included studies regarding an intervention type and total number of studies. (**G**) Outcome measures used in included studies: CL, clinical; L, laboratory; PROM, patient‐reported outcome measure (including subjective and quality of life); IMG, imaging. (**H**) The prevalence of core outcome measures used in included studies: SARA, Scale for the Assessment and Rating of Ataxia; 9‐HPT, 9‐Hole Peg Test; T25FW, Timed 25‐Foot Walk; mFARS, modified Friedreich Ataxia Rating Scale.^113^, ^114^ (**I**) Types of pharmacological interventions, n = 46: disease‐modifying n = 43, *symptomatic, n = 3. (**J**) Types of nonpharmacological interventions, n = 23: therapeutic approaches for ataxia management, n = 12; and management of other symptoms, n = 11. IGF‐1, Insulin‐like Growth Factor 1, RCT, randomized controlled trial; tDCS, transcranial direct current stimulation. [Color figure can be viewed at wileyonlinelibrary.com]

### Study Characteristics

Tables 1 and 2 and Figure 1 describe the characteristics of included studies for FA‐only patients, categorized by intervention type. Characteristics for mixed ataxia populations are available in Tables S4 and S5 and Figure S2.

**TABLE 1:** Characteristics of included studies with pharmacological interventions in Friedreich's ataxia (n = 46 studies)

Author (year of publication), country	Study design	Sample size (N)/Mean age ± SD (years)/FA characteristics^a^	Medication, route of administration/Dosage	Duration of treatment/follow‐up	Outcome type	Assessed feature/symptom	Outcome measure: result^b^	Comparator/Reported result
Disease modifying:								
Drinkard et al (2010)^32^ United States	RCT	42 13.2 ± 2.5 GAA1: 739 ± 230	Idebenone, orally 3 times a day (total dose: 5, 15, 45 mg/kg)	6 months treatment	L	Aerobic capacity	Peak VO_2_: 0 ns	Placebo
						Exercise capacity	Peak work rate: 0 ns	
					IMG	Cardiac function	Echo: no relationship between exercise and echo	
Lynch et al (2010)^34^ United States, Switzerland	RCT (IONIA)	70 13.7 ± 2.8 DD: 5.55 ± 3.19 y	Idebenone, orally Based on the weight at baseline: 450 mg daily (≤45 kg body weight) or 900 mg daily when >45 kg 1350 mg daily when ≤45 kg or 2250 mg daily when >45 kg	24 weeks (6 months)	CL	Ataxia severity	ICARS: (+) ns	Placebo
						Ataxia severity and functional impairment	FARS: (+) ns	
						Ataxia severity and functional impairment	FACT‐Z3: 0 ns	
						ADL	ADL: 0 ns	
Lagedrost et al (2011)^33^ United States, Switzerland					CL	Cardiac function	ECG: 0 ns	
					IMG	Cardiac function	Echo: 0 ns	
Meier et al (2012)^42^ United States, Switzerland	RCT (IONIA‐E)	68 14.0 ± 2.7 DD: 5.5 ± 3.2 y	Idebenone, orally Based on the body weight: 1350 or 2250 mg/day	6‐month treatment (controlled study) + 12‐month open label	CL	Ataxia severity and functional impairment	ICARS total: (−) ns Subscores speech, posture and stance: (−)^c^ FARS total: (−)^c^ Subscore speech: (+)^c^	12‐month open label
							ICARS total: (+) ns In all subscales: (+),^c^ except posture and stance: (−)^c^ FARS: (−)^c^, subscales speech, lower limb coordination: (+)^c^	18 months (6‐month controlled study + 12‐month open label)
Cook et al (2019)^46^ United Kingdom, Austria, Germany, Netherlands	RCT	29 37.1 ± 16.48^d^ DD: 18.39 ± 10.94^d^	Idebenone, orally 1350 mg/day if ≤45 kg, or 2250 mg/day if >45 kg	2 months of treatment or placebo (after the initial 12 months of treatment)	PROM	Perception of change	Change and status questionnaire: 0 ns	Placebo
						Fatigue	MFIS: 0 ns	
					CL	Manual dexterity and fine motor coordination	**9‐HPT:** 0 ns	
						Perception of change	CGI‐C: 0 ns	
						Speech intelligibility in dysarthria	AIDS: (+)^c^	
						Ataxia severity	ICARS: (+) ns ICARS: (+)^c^ for ambulatory patients	
Brandsema et al (2010)^115^ Canada	Quasi‐experimental	7 15.87 ± 2.21 AaO: 8.5 ± 2.78	Idebenone, orally 20 mg/kg/day twice daily	1‐year treatment	PROM (QoL)	QoL	PedsQL total: (+) ns, especially social and emotional scores PedsQL physical: (−)^c^	Pre‐ and posttreatment
					PROM	ADL	ADL: (+) ns	
					CL	Ataxia severity	ICARS total: 0 ns	
Lynch et al (2019)^35^ United States	RCT	92 (51 completed open‐label extension) 16.30 ± 4.10^d^ AaO: 9.30 ± 3.83^d^	Interferon‐γ, subcutaneously 3 times a week First 4 weeks: weekly dose escalation from 10/25/50/100 μg/m^2^	6‐month treatment + open‐label phase (1–14 months)	CL	Ataxia severity	FARS: 0 ns **mFARS:** 0 ns	Placebo
						Walking speed and mobility	T25W: 0 ns	
						Manual dexterity and fine motor coordination	**9‐HPT:** 0 ns	
						Vision	LCLA: 0 ns	
						ADL	ADL: 0 ns	
						Perception on disease	CGA: 0 ns	
					PROM	Perception on disease	PGA: n.d.	
						Fatigue	MFIS: 0	
					PROM (QoL)	QoL ≥18 years old	SF‐36 n.d.	
					PROM (QoL)C	QoL <18 years old	PedsQL caregiver n.d.	
					L	Frataxin level	Frataxin levels in the whole blood and buccal samples: 0 ns	
					CL	Ataxia severity	**mFARS:** (+) ns	Extension after 52 weeks
					L	Frataxin level	Frataxin levels in the whole blood and buccal samples: (+) ns	
Seyer et al (2015)^57^ United States	Quasi‐experimental study	12 Mean, range: 12 (8–17) AaO mean: 6	Interferon‐γ, subcutaneously 3 times a week Increasing dose: first 2 weeks: 10 μg/m^2^; next 2 weeks: 25 μg/m^2^; final 8 weeks: 50 μg/m^2^	12 weeks of treatment	L	Frataxin level	Frataxin levels in platelets and whole blood: (−),^c^ in buccal cell and red blood cells: (+)^c^	Pre‐and posttreatment
					CL	Ataxia severity and functional impairment	FARS total: (+)^c^, mainly upper extremities subscale	
						Walking speed, mobility	**T25FW:** 0 ns	
						Manual dexterity and fine motor coordination	**9‐HPT:** 0 ns	
						Vision	Visual acuity: (−) ns	
					PROM	Fatigue	MFIS: 0 ns	
						ADL	ADL: 0 ns	
					PROM(QoL) C	QoL	PedsQL: 0 ns (patient and caregiver)	
Marcotulli et al (2016)^41^ Italy	Quasi‐experimental study	9 29.78 ± 4.94 (21–38)^d^ AaO: 16.44 ± 5.17^d^	Interferon‐γ, subcutaneously, 3 administrations (at days 0, 14, 28) in doses 100, 150, 200 μg, respectively	28 days of treatment, 35 days of follow‐up	CL	Ataxia severity	**SARA:** 0 ns	Pre‐ and posttreatment
					L	Frataxin level	Frataxin levels: 0 ns	
Vavla et al (2020)^61^ Italy, United States	Quasi‐experimental study	12 17.33 ± 4.54 AaO: 8.5 ± 3.18	Interferon‐γ, subcutaneously Dose escalation: first 2 weeks 100 μg, following 22 weeks 200 μg	6 months of treatment, 6 months after treatment	CL	Ataxia severity	**SARA**—stabilized^c^ worsening trend after the discontinuation	Pre‐ and posttreatment
						Cardiac function	ECG (+)^c^	
					IMG		Echo: 0 ns	
					L	Frataxin level	Frataxin level in PBMCs: (+) ns	
Tekin and Levent (2022)^116^ Turkey	Case report	1 9 9	Interferon‐γ, subcutaneously 3 times a week Dose escalation: 14 days 10 μg/m^2^, then 14 days 25 μg/m^2^, then 50 μg/m^2^	Follow‐up: 112 days, 280 days (9 months)	CL	Ataxia severity and functional impairment	FARS: (+)	Pre‐ and posttreatment
						Manual dexterity and fine motor coordination	**9‐HPT:** 0	
						Walking speed and mobility	**T25FW:** 0	
					IMG	Cardiac function	Echo: minimal cardiological deterioration	
Lynch et al (2019)^47^ United States, Austria, Australia	RCT MOXIe part 1	69 25.6 ± 6.5 AaO: 15.3 ± 4.8	Omaveloxolone, orally, 2.5–300 mg/day, once daily	12 weeks	CL/L	Dose ranging	Optimal changes observed in doses 80–160 mg daily	Dosage 160 mg/day vs. placebo
					L	Exercise capacity	Peak workload: 0 ns	
					CL	Ataxia severity	**mFARS:** (+)^c^	
						Manual dexterity and fine motor coordination	**9‐HPT:** 0 ns	
						Walking speed and mobility	**T25FW:** 0 ns	
						Vision (low contrast)	LCLA: 0 ns	
					PROM(QoL)	QoL	SF‐36: 0 ns	
Lynch et al (2021)^48^, ^49^ United States, United Kingdom, Austria, Italy	RCT MOXIe study part 2	82 Full analysis set 23.89 ± 7.16^d^ AaO: 15.49 ± 5.48^d^	Omaveloxolone, orally, 150 mg daily	48 weeks of treatment + 4 weeks of follow‐up (52 weeks to 12 months)	CL	Ataxia severity	**mFARS:** (+)^c^ Subscores: upper limb coordination and upright stability	Placebo
					PROM	Perception of change	PGI‐C: (+) ns	
					CL	Perception of change	CGI‐C: (+) ns	
						Manual dexterity and fine motor coordination	**9‐HPT:** 0 ns	
						Walking speed and mobility	**T25FW:** 0 ns	
					PROM	Frequency of falls	Frequency of falls: (+) ns	
					L	Exercise capacity	Peak workload: (+) ns	
					CL	ADL	FA ADL: (+)^c^	
Lynch et al (2023)^73^ United States, Austria, Italy, United Kingdom	Quasi‐experimental study	82 23.89 ± 7.16^d^ AaO: 15.49 ± 5.48^d^	Omaveloxolone, orally 150 mg daily	72‐week extension (16 months) (up to 144 weeks)	CL	Ataxia severity	**mFARS:** (+) stable improvement, no worsening	Placebo
Lynch et al (2024)^86^ United States, Italy, Australia, Austria, Switzerland, United Kingdom	Quasi‐experimental study	136 from each study FACOMS, MOXIe Mean age: 26.2 ± 13.7; 26.6 ± 7.3 AaO: 15.2 ± 10.5; 15.5 ± 5.3	Omaveloxolone, orally 150 mg daily	Follow‐up: FACOMS: 2.54 ± 0.79 years MOXIe: 2.60 ± 0.52 years	CL	Ataxia severity and functional impairment	Long‐term (3‐year) efficacy of omaveloxolone **mFARS:** (+)^c^	FACOMS natural history data
Mariotti et al (2012)^36^ Italy	RCT	16 28.5 ± 5.5^d^ AaO: 12.67 ± 4.07^d^	Erythropoietin, intravenously Scaling up 20,000 IU every 3 weeks for 9 weeks, 40,000 IU every 3 weeks for 9 weeks, and 40,000 IU every 2 weeks for 6 weeks	6 months of scaling up	L	Frataxin level	Frataxin level: 0 ns	Between groups, Vs. placebo
					CL	Ataxia severity	**SARA:** 0 ns	
						Manual dexterity and fine motor coordination	**9‐HPT:** 0 ns	
					PROM(QoL)	QoL	SF‐36: 0 ns	
Nachbauer et al (2011)^75^ Austria	Quasi‐experimental study	5 Median age: 49 years (IQR 31–52) Median AaO: 21 years (IQR 10–23)	Erythropoietin, subcutaneously 3 times single injections: 5000 IU, 10,000 IU, 30,000 IU, in 1‐month intervals	3 months	L	Frataxin level	Frataxin: (+)^c^	Pre‐ and postintervention
						Iron metabolism	Reduced ferritin level: (+)^c^	
						Mitochondrial function	Mitochondrial function: 0 ns	
					CL	Ataxia severity	**SARA:** 0 ns	
Egger et al (2014)^117^ United States, Germany, Austria	Quasi‐experimental study	9 33.11 ± 10.68^d^ DD: 11.33 ± 5.43^d^	Erythropoietin, subcutaneously 5000 IU, 3 times a week/2 months + 6 months follow‐up 2000 IU 3 times weekly	8 weeks of treatment (for 2 patients 2 weeks) 10–12 months of follow‐up	IMG	White matter	DTI: fractional anisotropy in white matter: (+)^c^	Pre‐ and posttreatment
					CL	Ataxia severity and functional impairment	**SARA:** 7/9 (+) (at least −2 points) FARS: 7/9 (+) (at least −4 points) No correlation between DTI changes and changes in FARS and SARA	
Santner et al (2014)^77^ Switzerland, Austria, Germany	Quasi‐experimental study	9 33.11 ± 10.68^d^ DD: 11.44 ± 5.36^d^	Erythropoietin, subcutaneously 5000 IU, 3 times a week for 8 weeks, then 2000 IU, 3 times a week	Up to 6 months of treatment	IMG	Gray matter volume	MRI VBM: gray matter volume in pulvinar and posterior parietal cortex: (+)^c^	Pre‐ and posttreatment
					CL	Ataxia severity and functional impairment	**SARA**, FARS improvement correlated with VBM changes	
Costantini et al (2016)^69^ Italy	Quasi‐experimental study	34 36.3 ± 11.1 AaO: 17.1 ± 9.9	Thiamine (vitamin B_1_), intramuscular 100 mg, 2 times a week	Up to 24 months	CL	Ataxia severity	**SARA** total: (+)^c^	Pre‐and posttreatment
					PROM	Fatigue	FSS: 0 ns	
					CL	Deep tendon reflexes	Reappearance in 57% of patients with areflexia	
						Swallowing	Improvement in 63% of patients with dysphagia	
					IMG	Cardiac function	Echo +/−	
					L	FXN gene expression	FXN gene expression: 0 ns	
Costantini et al (2013)^118^ Italy	Case report	2 (39, 27) AaO: >24 years	Thiamine (vitamin B_1_), intramuscular 100 mg every 3 or 5 days	3 months of treatment	CL	Ataxia severity	**SARA:** (+) 58.9%	W39
					PROM	Fatigue	FSS: (+) 57.8%	
					CL	Ataxia severity	**SARA:** (+) 66.7%	M27
						Dysmetria	Dysmetria: (+)	
						Speech	Speech: (+)	
Zesiewicz et al (2018)^62^ United States	RCT	19 Median age, range Treatment: 34 (18–48) Comparator: 37 (23–47) AaO: <25 years	Linoleic acid, orally 1.8 g once daily or 4.5 g twice daily	28 days of treatment	L	Aerobic capacity	VO_2_ max: (+) ns	Placebo
						Exercise capacity	Peak workload: (+)^c^	
					CL	Ataxia severity and functional impairment	FARS‐Neuro: (+) ns	
						Walking speed and mobility	**T25FW:** 0 ns	
Lynch et al (2023)^74^ United States, Australia	RCT	65 recruited 45 completed the study 19.67 ± 7.99^d^ AaO: <26 y	Linoleic acid, orally, 1 capsule 960 mg First months: 9 capsules daily (3 × 3 × 3); later, 6 capsules daily (3 × 0 × 3)	11 months of treatment	L	Aerobic capacity	MVO_2_: 0 ns	Placebo
						Exercise capacity	Peak workload: 0 ns	
					CL	Functional mobility	T1MW: 0 ns	
						Ataxia severity	**mFARS:** (+)^c^	
						Perception of change	CGI‐C: 0 ns	
						Gait	GST: 0 ns (T1MW, peak workload, and MVO_2_)	
					PROM	Fatigue	In treatment group: modified FACIT: 0 ns	
Sanz‐Gallego et al (2014)^119^ Spain	Quasi‐experimental study	5 26.6 ± 5.4 DD: 9.4 ± 5.5 y	IGF‐1 (mecasermin), subcutaneously 50 μg/kg twice a day	12 months of treatment, 3 years of follow‐up	CL	Ataxia severity	**SARA** 2/5: (+)/(−)	Pre‐ and posttreatment 12 months/3 years
					IMG	Cardiac function	Echo: 0 ns	
					PROM(QoL)	QoL	SF‐36 3/5 fair satisfaction, 2/5 dissatisfied	
Garcia Ron and Rodriguez Mesa (2020)^68^ Spain	Case report	1 17 AaD: 11	IGF‐1 (mecasermin), subcutaneously Initially, 0.04 mg/kg twice daily, gradually increased to 0.12 mg/kg twice daily	Up to 36 months of follow‐up	CL	Ataxia severity	**SARA** total: (+) (9 points), stable over 36 months	Pre‐ and posttreatment 3 months/36 months vs. previous time points
					IMG	Cardiac function	Echo: (+)	
					PROM(S)	Self‐perceived physical function	Energy level and coordination: (+)	
Sacca et al (2016)^59^ Italy	RCT	56 35.8 ± 14.2 AaO: 19.3 ± 9.7 years	Epoetin alfa, subcutaneously 1200 IU/kg every 12 weeks, (4 administrations)	24 and 48 weeks of follow‐up	L	Aerobic capacity	VO_2_ max at the CPET: (+) ns	Between groups (vs. placebo), 24/48 weeks
						Frataxin level in PBMCs	Frataxin level: 0 ns	
					IMG	Cardiac function	Echo: 0 ns	
					CL	Ataxia severity	**SARA:** 0 ns	
						Manual dexterity and fine motor coordination	**9‐HPT:** (+)^c^/(+)^c^	
					PROM(QoL)	QoL	EQ‐5D: 0 ns	
					PROM	ADL	ADL/IADL: 0 ns	
Sacca et al (2011)^76^ Italy	Quasi‐experimental study	10 29 ± 8.2 AaO: 17.2 ± 6.4 years	Epoetin alfa, subcutaneous 1× 600 IU/kg 3 months washout 1× 1200 IU/kg	Up to 6 months after the last dose	L	Frataxin level	Frataxin levels: (+) (gradual increase 3 months after first dose and up to 6 months after the second dose)	Pre‐ and posttreatment
						Iron metabolism	Total iron: (+)^c^ Ferritin: (+)^c^	
					CL	Cardiac function	ECG: 0 ns	
						Ataxia severity	ICARS: 0 ns At frataxin peak: 0 ns	
					IMG	Cardiac function	Echo: 0 ns	
Pandolfo et al (2014)^43^ Belgium, Spain, Italy, Australia, France, Canada	RCT	72 18.89 ± 7.73^d^ GAA repeat ≥400 on the shorter allele	Deferiprone, orally 20/40/60 mg/kg/day	6 months	CL	Manual dexterity and fine motor coordination	**9‐HPT:** 0 ns/0 ns	20 mg vs. placebo/40 mg vs. placebo
						Walking speed, mobility	**T25FW:** 0 ns/0 ns	
						Vision (low contrast)	LCLA: (−) ns/(−) ns	
						Ataxia severity	ICARS: 0 ns/(−)^c^	
						Ataxia severity and functional impairment	FARS: 0 ns/(−)^c^	
					IMG	Cardiac function	Echo: 0 ns in LVSF, LVEF Echo: (+)^c^ LV	
					CL	ADL	FARS ADL: 0 ns/(−)^c^	
						Ataxia severity and functional impairment	Worsening of ataxia and adverse effects	60 mg vs. placebo
Elincx‐Benizri et al (2016)^120^ Israel	Case series	5 24.0 ± 8.0^d^ DD: 14.2 ± 6.3^d^	Deferiprone, orally 20 mg/kg/day in 2/3 divided doses, + idebenone	12–24 months of follow‐up	CL	Ataxia severity	**SARA** 2/5: (+)	Pre‐ and posttreatment (12 and 24 months)
						Ataxia severity and functional impairment	FARS 3/5: (+)	
						Manual dexterity and fine motor coordination	**9‐HPT** (R) 3/5: (+) **9‐HPT** (L) 2/5: (+)	
					IMG	Cardiac function	Echo 3/5: (+)	
					PROM(QoL)	QoL	SF‐36 1/5: (+)	
Lynch et al (2012)^71^ United States	RCT	31 31.2 ± 11.7 Shorter triplet repeat length 699 ± 205	A0001 (alfa‐tocopheryl quinone), orally 500 or 750 mg twice a day	28 days of treatment	CL	Ataxia severity and functional impairment	FARS (+)^c^/(+)^d^ [FARS subscore lower limb (+)^c^ for a high dose]	Low dose vs. placebo/high dose vs. placebo
					PROM(QoL)	QoL	SF‐36 (PFSLS): (−) ns/0 ns	
					PROM	Fatigue, life satisfaction	MFIS LS: (+) ns in high dose	
					CL	Walking speed, mobility	**T25FW:** 0 ns	
						Manual dexterity and fine motor coordination	**9‐HPT:** 0 ns	
						Perception of disease	GICS LS: (+) ns in high dose	
					PROM	ADL	ADL: 0 ns	
Boesch et al (2014)^31^ Austria, Italy, Germany, Denmark	RCT	36 27.8 ± 8.7 DoS: 12.03 ± 5.69^d^	Carbamylated erythropoietin (CEPO), intravenously 325 μg three times/week	2 weeks of treatment Up to 43 days of follow‐up after baseline (ataxia measures)	L	Frataxin level in PBMCs and buccal cells, mRNA	Frataxin levels: 0 ns	Between groups
						Oxidative stress	8‐OHdG: 0 ns	
					CL	Ataxia severity	**SARA:** 0 ns	
						Ataxia severity and functional impairment	FARS: 0 ns	
						Perception of severity	CGI‐S: 0 ns	
Paparella et al (2024)^50^ Italy	RCT	35 (30 adults, 5 children) 25.0 ± 7.1 AaO: 11.8 ± 5.7 years	Etravirine, orally, 200 or 400 mg/day	4 Months of treatment Follow‐up 4 months posttreatment	L	Aerobic capacity	Peak VO_2_: 0 ns	Between groups
						Exercise capacity	Peak workload (+)^c^	
					CL	Ataxia severity	**SARA:** (+)^c^ after 2 and 4 months	
					IMG	Cardiac function	Echo: 0 ns	
					PROM(QoL)	QoL	SF‐36 in vitality: (+)^c^ Gradual^c^ worsening after the discontinuation	
					PROM	Disability and functioning across life domains	WHODAS: (+) mobility, self‐care, life activities Gradual worsening after the discontinuation	
					L	Frataxin levels in PBMCs, mRNA	Frataxin: n.d.	
Pandolfo et al (2022)^37^ Belgium, Spain, United States, France	RCT	39 24.0 ± 10.7 AaO: 14.3 ± 7.5 years	Leriglitazone, orally Based on age and sex: 150 mg adult males, 130 mg adult females, 2.2 mg/kg in adolescents (12–17 years old) Dose modification after 4 weeks	48 weeks of treatment (11 months)	IMG	Spinal cord structure	MRI of the spinal cord area C2‐C3: 0 ns	Placebo
					IMG	Cardiac function	Echo: 0 ns	
					CL	Cerebellar dysfunction of upper limb	CCFS: (−) ns	
						Ataxia severity	**SARA:** 0 ns	
						Manual dexterity and fine motor coordination	**9‐HPT:** 0 ns	
						ADL	FARS ADL: 0 ns	
						Perception of improvement	CGI‐I: 0 (but n.i.)	
						Perception of severity	CGI‐S (but n.i.)	
					PROM	Perception of improvement	PGI‐I (but n.i.)	
					PROM(QoL)	QoL	EQ‐5D‐5L: 0 ns	
Wang et al (2021)^38^ United States	RCT	67 (63 completed a study) 31.07 ± 10.22^d^ A disease stage 2–5 according to FA functional disability stage	Luvadaxistat, orally 75 or 300 mg twice daily	12 weeks of treatment + phone follow‐up	CL	Manual dexterity and fine motor coordination	**9‐HPT:** 0 ns	Placebo
						Ataxia severity	**mFARS‐Neuro:** (+) ns	
						Walking speed and mobility	**T25FW:** 0 ns	
						ADL	FARS ADL: 0 ns	
					PROM	Perception of severity	PGI‐S: 0 ns	
					CL	Perception of improvement	CGI‐I: 0 ns	
						Perception of severity	CGI‐S: 0 ns	
						Vision (low contrast)	LCLA: 0 ns	
					PROM(S)	Self‐perceived physical function	No changes/no help (63%), improvement in the upper extremity motor function and manual dexterity during the trial (37%)	
Zesiewicz et al (2018)^72^ United States	RCT	63 29.17 ± 7.61^d^ Sum of GAA repeats: 1442 ± 301.4^d^	Vatiquinone, orally 200 or 400 mg 3 times daily	24 months (6 months placebo controlled, 18 months open label)	CL	Vision (low contrast)	LCLA: 0 ns	Placebo after 6 months
						Ataxia severity	FARS‐Neuro: 0 ns	Lower dose vs. placebo
						Walking speed and mobility	**T25FW:** 0 ns	
						Manual dexterity and fine motor coordination	**9‐HPT:** 0 ns	
					IMG	Cardiac function	Echo: 0 ns	
					CL	Ataxia severity	FARS‐Neuro: (+)^c^ 73% (vs. 43) ≥ 3 points, 64% (vs 33) ≥ 5 points	
						Ataxia severity	FARS: (+)^c^	After 24 months, natural history data
Alemany‐Perna et al (2024)^44^ Spain	Quasi‐experimental study	20 (15 patients received treatment) 29.8 ± 13.9^d^ AaO: 16.90 ± 9.27 years^d^	Calcitriol, orally, (active form of vitamin D) 0.25 μg once a day	12 months of treatment	CL	Ataxia severity	**SARA:** (−) ns	Pre‐ and posttreatment
						Manual dexterity and fine motor coordination	**9‐HPT:** 0 ns	
						Walking speed	8‐MWT: (−)^c^	
						Speech motor control	PATA test: 0 ns	
						Independence in daily activities	Barthel Index: 0 ns	
					PROM(QoL)	QoL	SF‐36 total: 0 ns [subscore: reported health transition (+)^c^]	
					L	Frataxin level	Frataxin levels (+)^c^	
Arpa et al (2013)^39^ Spain	Quasi‐experimental study	9 28 ± 8 DD (n = 8): 15.5 ± 9.9^d^	Darbepoetin alfa, route of administration not specified (subcutaneously or intravenously) 150 μg every 2 or 3 weeks Idebenone, orally 10–20 mg/kg/day, twice daily Riboflavin, orally 10–15 mg/kg/day, twice daily	Mean treatment (range) 32 ± 19.4 months (8–56)	CL	Ataxia severity	**SARA:** 0 ns	Pre‐and posttreatment, 2 years
					PROM(QoL)	QoL	SF‐36v2 44.4% felt better (2 years), 22.2% dissatisfied	
					IMG	Cardiac function	Echo: +/−	
					CL	Ataxia severity	**SARA:** (+)	Vs. annual worsening rate
Qureshi et al (2021)^121^ United States	Quasi‐experimental study	10 14.5 years (10–22) DD mean: 4.5 years	(+)‐epicatechin, orally 75 mg daily (150 mg daily at 12 weeks, when no improvement)	24 months of treatment	CL	Ataxia severity and functional impairment	FARS/**mFARS:** (+) ns FARS: 56% (+)	Pre‐ and posttreatment
						Walking speed	8‐MTW: 33% (+)	
						Manual dexterity and fine motor coordination	**9‐HPT:** 60% (+)	
					IMG	Cardiac function	Echo: 0 ns	
						Spinal cord	DTI anisotropy: 0 ns	
Igoillo‐Esteve et al (2020)^122^ Belgium, Italy, United States	Quasi‐experimental study	16 32 ± 3 AaO: 14 ± 1	GLP‐1 analogues, subcutaneously Exenatide: 5 μg twice daily—1 week, 10 μg twice daily—4 weeks Or liraglutide: 0.6 mg once daily, 1.2 mg/kg once daily—next 2 weeks, 1.8 mg/kg—last 2 weeks	5‐week GLP‐1 analogue treatment; and 4‐week washout	L	Frataxin expression in PBMCs and platelets, mRNA	Frataxin levels: (+)	Pre‐ and posttreatment, exenatide
					CL	Ataxia severity	**SARA:** 0 ns	
						Noncerebellar signs	INAS: 0 ns	
						ADL	ADL: 0 ns	
Patel et al (2019)^45^ United States	Quasi‐experimental study	11 (8 children, 3 adults) Median (range): 12 years (9–65) GAA repetition, short allele: 99–900	Methylprednisolone, orally At baseline, 48 mg (6 × 8‐mg tablets), then 7 cycles, 28 days each: 6‐day treatment, dose tapering: 8 mg (1 tablet) each day	26 weeks	CL	Walking speed and mobility	**T25FW:** 0 ns	Pre‐ and posttreatment
						Ataxia severity and functional impairment	FARS: 0 ns	
						Functional mobility	T1MW: (+)^c^ in children subgroup	
						Manual dexterity and fine motor coordination	**9‐HPT:** 0 ns	
					PROM	ADL	ADL: 0 ns	
						Fatigue >18	MFIS: 0 ns	
					PROM(QoL)	QoL >18	SF‐36: 0 ns	
					CL	Perception of improvement	CGI: 0 ns	
					PROM	Perception of improvement	PGI: (+)^c^ in overall population	
					L	Frataxin level	Frataxin level: 0 ns (n.d.)	
					CL	Ataxia severity and functional impairment	**mFARS:** 0 ns	
Libri et al (2014)^58^ United Kingdom, Germany, China, New Zealand	Quasi‐experimental study	Phase 1: n = 10; mean age: 33 years AaO: 17.9 ± 8.1 years Phases 2 and 3: n = 10; mean age: 33 years AaO: 21.7 ± 9.5 years	Nicotinamide, orally Phase 1: escalating doses: 0–8 g Phase 2: escalating doses 3.5–6 g for 5 days Phase 3: maximum tolerated doses daily for 8 weeks	Phase 2: 5 days Phase 3: 8 weeks	L	Frataxin level	Dose–response relation with frataxin protein concentration^c^	Phase 1
							Sustained frataxin upregulation	Phase 2, 3 Pre‐ and posttreatment
					CL	Ataxia severity	**SARA:** 0 ns	
						Composite ataxia scale (8 MW + 9‐HPT + PATA)	**SCAFI:** 0 ns	
						Walking speed	8 MW: 0 ns	
						Manual dexterity and fine motor coordination	**9‐HPT:** 0 ns	
						Speech motor control	PATA: 0 ns	
						Speech understandability	SIT: 0 ns	
					PROM C	ADL	FARS ADL: (+) ns (caregivers)	
Yiu et al (2015)^60^ Australia, Austria, China	Quasi‐experimental study	24 34.9 ± 12.7 (low dose) 39.2 ± 7.7 (high dose) 37.05 ± 10.50^d^ AaO (years): 15.6 ± 6.1 (low‐dose) 19.7 ± 7.7 (high dose) 17.65 ± 7.11^d^	Resveratrol, orally 0.5 or 2.5 g twice daily	12 weeks	L	Frataxin level in PBMCs	Frataxin level: (+) ns/(+) ns	Low dose/high dose
						Frataxin mRNA	FXN mRNA: 0 ns/0 ns	
						Oxidative stress markers	0 ns/(+)^c^	
					CL	Ataxia severity and functional impairment	FARS: (+) ns/(+)^c^ (mostly upper limb and bulbar components)	
						Ataxia severity	ICARS: 0 ns/(+)^c^ (mostly in posture)	
						Ataxia severity	**SARA:** 0 ns/(+) ns	
						Functional impairment	FACT Z2, Z3: 0 ns/0 ns	
						Speech assessment	CNC word list: 0 ns/(+)^c^	
					PROM	Impact of FA on daily living	FAIS: 0 ns/0 ns	
					PROM(QoL)	QoL	SF‐36‐v2: 0 ns/0 ns	
					IMG	Cardiac function	Echo: 0 ns/0 ns	
					CL	Vision (low contrast)	LCLA: 0 ns/0 ns	
						Walking speed and mobility	**T25FW:** 0 ns/0 ns	
						Manual dexterity and fine motor coordination	**9‐HPT:** 0 ns/0 ns	
Bolotta et al (2020)^40^ Italy	Quasi‐experimental study	7 (5 completed the study) 21.43 ± 5.91^d^ AaO: 9.57 ± 4.04 years^d^	Tocotrienol, orally 5 mg/kg/day	12 months	CL	Ataxia severity	ICARS: (−) ns	Pre‐ and posttreatment
					IMG	Cardiac function	Cardiac MRI: (+) ns	
Symptomatic treatment								
Berntsson et al (2013)^123^ Sweden	Case report	1 50 AaD: 31	Baclofen 25 μg—test, then via the ITB‐delivery pump, 100 μg daily	2 years Follow‐up	PROM(S)	Spasm self‐assessment	Relief of spasms	Pre‐ and posttreatment
Kalyvas et al (2018)^124^ Greece	Case report	1 65 AaO: 22	Baclofen 50 μg—test, then intrathecal delivery pump 80 μg daily (80–95 μg/day)	3 years Follow‐up	PROM(S)	Spasm self‐assessment	Relief of spasms, reduced frequency of spasm	Pre‐ and posttreatment
						Self‐assessment of physical function	Pain relief, sleep improvement	
Peter et al (2016)^125^ Austria	Case report	1 24 10	Memantine, orally n.i.	1‐year follow‐up	L	Optic nerve assessment	OCT: no further loss of the nerve fiber layer	Pre‐ and posttreatment
						Vision	Visual acuity stable	
					PROM(S)	Subjective assessment	Slight deterioration in vision but clear progression of loss of visual acuity	

^a^ FA characteristics: AaO, DD, DoS, DS, AaD, or YsD.

^b^ Core outcome measures results: +, improvement; −, deterioration regardless of the treatment; 0, no effect.

^c^ Significant effect.

^d^ Mean (age at onset, disease duration) calculated from the available data in the publication.

Abbreviations: 8‐MWT, 8‐Meter Walking Test; 8‐OHdG, 8‐hydroxydeoxyguanosine (oxidative stress marker); 9‐HPT, 9‐Hole Peg Test; AaD, age at diagnosis; AaO, age at onset; ADL, activities of daily living; AIDS, assessment of intelligibility of dysarthric speech; C, caregiver; CCFS, Composite Cerebellar Functional Scale; CGA, Clinical Global Assessment; CGI‐C/‐S/‐I, Clinical Global Impression of Change/Severity/Improvement; CL, clinical; CNC, consonant‐nucleus‐consonant word list; CPET, maximal/the highest oxygen consumption during exercise (Cardiopulmonary Exercise Test); DD, disease duration; DoS, duration of symptoms; DS, disease stage; DTI, diffusion tensor imaging; ECG, electrocardiography; echo, echocardiography; EQ‐5D(‐5L), EuroQol 5‐dimension (5‐level) questionnaire; FA, Friedreich's ataxia; FACIT, Functional Assessment of Chronic Illness Therapy Fatigue Scale; FACOMS, Friedreich's Ataxia Clinical Outcome Measures Study; FACT Z2/Z3, Friedreich's Ataxia Composite Test (composite of 2 *Z* scores from 9‐HPT and T25FW/composite of 3 *Z* scores from 9‐HPT); FAIS, Friedreich Ataxia Impact Scale; FARS, Friedreich Ataxia Rating Scale; FARS ADL, Friedreich Ataxia Rating Scale subscore activities of daily living; FARS‐Neuro, Friedreich Ataxia Rating Scale neurological function; FSS, fatigue severity scale; FXN, Frataxin; GAA, guanine–adenine–adenine trinucleotide repeat; GICS LS, global impression of clinical severity (life satisfaction); GLP‐1 Glucagon‐like Peptide‐1; GST, gait speed test; IADL, instrumental activities of daily living; ICARS, International Cooperative Ataxia Rating Scale; IGF‐1, Insulin‐like Growth Factor 1; IMG, imaging; INAS, inventory of non‐ataxia symptoms; IONIA, clinical trial on idebenone; IONIA‐E, clinical trial on idebenone ‐ extention; ITB, Intrathecal Baclofen; IQR, interquartile range; L, laboratory; LCLA, low‐contrast letter acuity; LV, Left Ventricle; LVEF, Left Ventricular Ejection Fraction; LVSF, Left Ventricular Shortening Franction; mFARS, modified Friedreich Ataxia Rating Scale; MFIS (LS), modified fatigue impact scale (life satisfaction); MOXIe, clinical trial on omaveloxolone; MRI, magnetic resonance imaging; mRNA, messenger RNA; MVO_2_, maximum oxygen consumption; n.i., no information; ns, not significant; OCT, optical coherence tomography; PATA, PATA speech task; PBMC, Peripherial Blood Mononuclear Cells; PedsQL, pediatric quality of life inventory; PGA, patient global assessment; PGI, patient global impression; PGI‐C/‐I/‐S, patient's global impression of change/improvement/severity; PROM, patient‐reported outcome measure; QoL, quality of life; RCT, randomized controlled trial; S, subjective; SARA, Scale for the Assessment and Rating of Ataxia; SCAFI, Spinocerebellar Ataxia Functional Index; SD, standard deviation; SF‐36 (PFSLS), Short Form Health Survey with 36 questions (Physical Functioning subscale); SIT, speech intelligibility test; T1MW, Timed 1‐Minute Walk Test; T25FW, Timed 25‐Foot Walk test; VBM, voxel‐based morphometry; VO_2_ max, maximal oxygen uptake; VO_2_ peak, peak oxygen uptak; WHODAS, World Health Organization disability assessment schedule; YsD, years since diagnosis.

**TABLE 2:** Characteristics of included studies with nonpharmacological interventions in Friedreich's ataxia (n = 23 studies)

Author (year), country	Study design	Sample size (N)/Mean age ± SD (years)/FA characteristics^a^	Intervention details	Duration of intervention/follow‐up	Outcome type	Assessed feature/symptom	Outcome measure: result^b^	Comparator, reported result
Rehabilitation, physiotherapy, sport								
Seco Calvo et al (2014)^126^ Spain	RCT	16 53.31 ± 5.65 AaD: 12.33 ± 1.82	Rehabilitation (physical condition, specific physical condition, prefunctional training, and functional training) + pharmacological treatment	5‐year program, 2‐year follow‐up	CL	Ataxia severity	ICARS: (+)^c^	Between groups after follow‐up
						Functional independence	FIM: 0 ns	
					PROM(QoL)	QoL	SF‐36: 0 ns	
Milne et al (2018)^66^ Australia	RCT	19 36.88 ± 12.26^d^ AoO: 14.79 ± 8.59^d^	Rehabilitation (strengthening, postural control, coordination and control, functional mobility, balance training, stretching, cardiovascular fitness)	6 weeks inpatient + 6 weeks home based	CL	Functional independence	FIM: 0 ns	Between groups after 6 weeks
						Ataxia severity	FARS: 0 ns	
						Balance	BBS for ambulant patients: (+) ns	
					PROM	Impact of FA on daily living	FAIS movement subscale: (+)^c^ FAIS ADL subscale: 0 ns	
						Perception of change	PGI‐C: (+)^c^	
Battal et al (2023)^127^ Lebanon, France	Case report	1 26 AaO: 16	Rehabilitation + home adjustment (therapeutic adaptive equipment)	3‐Month intervention	CL	Ataxia severity	**SARA:** (+) (−9 points)	Pre‐ and postintervention
						Functional independence	FIM: (+) (+30 points)	
					PROM(S)	Self‐perceived physical function	Feeling stronger, more confident, steadier	
Multidisciplinary therapy/Combined therapy (rehabilitation + other)								
Paparella et al (2023)^56^ Italy	Quasi‐experimental study	42 (29 adults, 13 children) 20.7 ± 8.6 AaO: 11.7 ± 7.7 years	Intensive rehabilitation (physiotherapy, occupational therapy, practical manual activities, psychological support, speech therapy)	3 weeks for children 4 weeks for adults	CL	Ataxia severity	**SARA:** (+)^c^	Pre‐ and postintervention, results for the whole cohort
						Ataxia severity and functional impairment	FARS total: (+)^c^	
						Manual dexterity and fine motor coordination	**9‐HPT:** (D, ND) 0 ns	
Samardzic (2023)^128^ Montenegro	Case report	1 26 AaO: 21	Multicomponent physical therapy with proprioceptive neuromuscular facilitation stabilization techniques, manual massage, occupational therapy	3 weeks of rehabilitation	CL	Ataxia severity	**SARA** total: (+) (− 4 points)	Pre‐ and postintervention
						Functional independence	FIM (locomotion): (+) (+2 points)	
Teixeira et al (2018)^129^ Brazil	Case report	1 29 AaD: 17	Dance (contemporary dance, belly dance) and somatic education	8 months	PROM	Impact of educational dance	IAADE: (+) (in all skills: to get up, to sit, shifting feet, shifting the ground, sitting position, body image, joint mobility, movement rhythm, fluency movement)	Pre‐ and postintervention
Heart interventions								
Yoon et al (2012)^51^ Canada	Case report	1 14 AaD: 11	Heart transplantation	20‐month follow‐up	CL	Ataxia severity and functional impairment	FARS: (−) (+97) (mainly upper limb coordination, upright stability/ambulation)	Pre‐ and postintervention
					PROM(QoL)	QoL	PedsQL total: (+) (+5) (subscale physical functioning +21, psychosocial −1)	
					IMG	Cardiac function	Echo (+)	
Ivak et al (2016)^52^ Czech Republic	Case report	1 23 AaD: 16		6‐month follow‐up 1, 2, 3, 4, 5, 6, 7, 8 years	CL	Ataxia severity and functional impairment	FARS total: (+) (−86) FARS total: (+) (−28) FARS total: (−) (+58)	Preintervention and 6 months postintervention/before surgery vs. 8 years after 6 months after surgery vs. 8 years after
					IMG	Cardiac function	Echo: (+)	
McCormick et al (2017)^53^ United States	Case report	3 Child, 37, 37 AaD: 12, 24, 34		19‐, 5‐, and 8‐year follow‐up	CL	Neurological function	Neurological function (−)	Pre‐ and postintervention
					IMG	Cardiac function	Echo (+)	
Valero et al (2022)^130^ Spain	Case report	1 58 AaO: 33		2.5 months after transplant	CL	Neurological function	Neurological function stable	Pre‐ and postintervention
					IMG	Cardiac function	Echo stable	
Anderson et al (2016)^131^ United States	Case report	1 Child AaD: 4	Septal myectomy	7‐year follow‐up	IMG	Cardiac function	Echo (+)	Pre‐ and postintervention
Hayiroglu et al (2025)^132^ Turkey	Case report	1 31 n.i.	Catheter ablation	1‐year follow‐up	IMG	Cardiac function	Echo (+)	Pre‐ and postintervention
					CL		ECG (+)	
tDCS								
Naeije et al (2023)^70^ Belgium	RCT	24 Median age, range: 32 (15–66) Median AaO, range: 17 (6–45)	Anodal cerebellar tDCS	1‐week treatment	CL	Ataxia severity	**SARA:** (+)^c^ anodal tDCS 0 ns in sham	Pre‐ and postintervention, within groups
						Cognitive and emotional changes (nonmotor cerebellar function)	CCAS‐S: (+)^c^ anodal tDCS 0 ns in sham	
Portaro et al (2019)^133^ Italy	Case report	1 29 AaO: 10	Robotic gait training, with a virtual reality game (Lokomat Pro) + Cerebellar anodal tDCS	8‐week follow‐up	CL	Ataxia severity	**SARA** total: (+) (−4) (subscales: gait, stance, sitting, heel‐shin slide)	Pre‐ and postintervention
					L	Cerebellar modulation of motor cortex	CBI: (+) (−18)	
Auditory problems								
Rance et al (2010)^63^ Australia	Quasi‐experimental study	10 24.80 ± 11.43^d^ AaO: 10.10 ± 4.59^c^	Use of personal FM‐listening devices (Phonak Inspiro FM‐transmitter paired with bilateral ISense ear‐level receivers)	6‐week device trial	CL	Hearing assessment	ABR, frequency, AM detection (−) vs. healthy control	Pre‐ and postintervention
					PROM	Hearing with the hearing aid	APHAB (+) (+)^c^ in communication, noise, reverberation scores	
Frewin et al (2013)^65^ United Kingdom	Case report	1 41 AaD: 10	Bilateral cochlear implantation + Rehabilitation (audiobooks, auditory training materials)	2 months of implant use (right unilateral) 2 months—bilateral	CL	Speech discrimination performance	Unilateral and bilateral: BKB sentences (+)	Pre‐ and postintervention
					PROM(QoL)	QoL	Unilateral: EuroQol 0	
						QoL in cochlear implant users	Unilateral: NCIQ (+) (especially social domain)	
Occupational therapy								
Ciancarelli et al (2011)^54^ Italy	Quasi‐experimental study	10 26.9 ± 8.2 n.i.	Occupational therapy + Intensive conventional neuromotor rehabilitation program	12 months	CL	Independence in daily living	Barthel Index (+)^c^	Pre‐ and postintervention
						Physical performance	Physical performance test (−)^c^	
						Basic daily living independence	Katz Index (+)^c^	
						Cerebellar dysfunction	Klockgether Scale: 0 ns	
						Manual dexterity and fine motor coordination	**9‐HPT:** 0 ns	
Zanona et al (2015)^134^ Brazil	Case report	2 28, 36 AaD: 8, AaO: 12	Occupational therapy	5 months of therapy	CL	Functional independence	FIM: (+)	Pre‐ and postintervention
						Motor performance	Muscle strength: 0 Range of motion: 0	
Vibration								
Herrero et al (2011)^64^ Spain	Quasi‐experimental study	10 38.1 ± 10.5 AaD: 14.3 ± 6.4	Whole‐body vibration (WBV)	3 weeks	L	Circulation in muscles	Blood flow velocity: (+)^c^	Pre‐ and postintervention
						Neuromuscular activity	EMG amplitude: (+)^c^	
					PROM	Exercise intensity	RPE: (+) by higher vibration	
Neuromuscular electrical stimulation								
McGarrell et al (2025)^135^ United States	Case report	1 36 n.i.	Neuromuscular electrical stimulation (NMES)	1 month	L	Muscle endurance	EI: (+)	Pre‐ and postintervention
						Mitochondrial capacity	Mitochondrial capacity: 0	
						Muscle response to a single stimulus	Twitch contractions: (+)	
Scoliosis management								
Leighton et al (2013)^55^ Canada	Case report	2 (twin sisters) 14 Early onset	Posterior spinal fusion	Follow‐up: 2, 6, 25 weeks, 2 years	CL	Physical function	Standing alignment: (+) Pain: (+) Gait and endurance: (−) Stable sitting position: (+) Upper extremities function: 0	Pre‐ and postintervention
					PROM (S) C	Satisfaction	Satisfaction of patients and caregivers: (+)	
Speech therapy								
Batista and Conceição (2025)^136^ Brasil, Australia	Case report	1 23 AaO: 23	Speech therapy (in‐person) Expressiveness Development Program for Oral Communication	n.i.	CL	Speech assessment	Auditory‐perceptual evaluation: Reading: (+) Absence of vocal deviation: (+) Adequate of vocal frequency: (+) adequate speech rate: (+)	Pre‐ and postintervention
					PROM (S)		Diction: 0 Sound of the voice: (+) Speaking experience: (+)	
Cognitive therapy								
Ciancarelli et al (2010)^67^ Italy	Case series (case report)	24 32.48 ± 10.81 DD: 13.38 ± 7.98 years^d^	Cognitive therapy (modules and tasks to develop several skills, eg, memory, attention and concentration, language and literacy, visuospatial processing, daily life and functional skills, reading and writing)	6 days a week during three separate 3‐week hospital stays 1‐year follow‐up	CL	Cognitive function	MMSE: 0 ns	Pre‐ and postintervention
						Long‐term memory	The Rey 15‐Item Memorization Test for long‐term memory: 0 ns	
						Nonverbal reasoning/fluid intelligence	RCPM: 0 ns	
						Executive function and language	Phonemic Verbal Fluency Test: 0 ns	
						Processing speed and attention	SDMT: 0 ns	
						Short‐term memory	Rey 15‐Item Memorization test for short‐term memory: (+)^c^	
						Cognitive flexibility and inhibition	Stroop Colour‐Word Interference Task: (+)^c^	
					PROM	Depression symptoms	Zung SDS: 0 ns	

^a^ FA characteristics: AaO, DD, DS, AaD, or YsD.

^b^ Core outcome measures results: +, improvement; −, deterioration regardless of the treatment; 0, no effect.

^c^ Significant effect.

^d^ Mean (age at onset, disease duration) calculated from the available data in the publication.

Abbreviations: 9‐HPT (D, ND), 9‐Hole Peg Test (dominant, nondominant); AaD, age at diagnosis; AaO, age at onset; ABR, auditory brainstem response; AM, amplitude modulation; APHAB, abbreviated profile of hearing aid benefit; BBS, Berg Balance Scale; BKB, Sentences Bamford–Kowal–Bench Sentences; C, caregiver; CBI, Cerebellar Brain Inhibition; CCAS‐S, Cerebellar Cognitive Affective Syndrome Scale; CL, clinical; DD, disease duration; DS, disease stage; ECG, electrocardiography; Echo, echocardiography; EI, endurance index; EMG, electromyography; EuroQol/EQ‐5D (5 L), EuroQol 5‐dimension (five‐level) questionnaire; Friedreich's ataxia; FAIS, Friedreich Ataxia Impact Scale; FAIS ADL, Friedreich's Ataxia Impact Scale Activities of Daily Living; FARS, Friedreich Ataxia Rating Scale; FIM, Functional independence measure; IAADE, Instrumento de Avaliação da Aprendizagem na Dança Educacional (Monitoring Instrument Learning in Educational Dance); ICARS, International Cooperative Ataxia Rating Scale; IMG, imaging; L, laboratory; MMSE, Mini‐Mental State Examination; NCIQ, Nijmegen Cochlear Implant Questionnaire; n.i., no information; ns, not significant; PedsQL, Pediatric Quality of Life Inventory; PGI‐C, patient global impression–change; PROM, patient‐reported outcome measure; QoL, quality of life; RCPM, Raven's coloured progressive matrices; RCT, randomized controlled trial; RPE, rate of perceived exertion; S, subjective; SARA, Scale for the Assessment and Rating of Ataxia; SD, standard deviation; SDMT, symbol digit modalities test; SF‐36, Short Form Health Survey with 36 questions; tDCS, transcranial direct current stimulation; YsD, years since diagnosis; Zung SDS, Zung self‐rating depression scale.

Almost all included studies were written in English (n = 86; 96%), with only four publications written in languages other than English. Most studies with FA‐only patients (n = 69) were conducted in Europe (n = 29; 42.0%), North America (n = 15; 21.7%), or were international studies (n = 20; 29%). The majority of studies included pharmacological interventions (n = 69; 66.7%), with only 33.3% (n = 23) investigating nonpharmacological interventions (Fig. 1D).

Most pharmacological studies were quasi‐experimental studies (n = 20; 43.5%) or RCTs (n = 19; 41.3%), whereas nonpharmacological interventions were mostly case reports including case series (n = 16; 69.6%) and quasi‐experimental studies (n = 4; 17.4%) (Fig. 1F). Considering all studies, the prevalence of RCTs, quasi‐experimental studies, and case reports was similar (32%, 35%, and 33%, respectively) (Fig. 1F). Sample sizes were small, with only one pharmacological study having more than 100 patients.

### Risk of Bias

Studies differed in levels of bias and strength of evidence, from RCTs with the highest reliability, through the quasi‐experimental studies, up to case reports with the highest bias. The summary of risk of bias assessment for specific study designs is presented in Figure 2. The details from the performed assessments are available in Tables S5 to S10 and Figure S3.

**FIG. 2:**
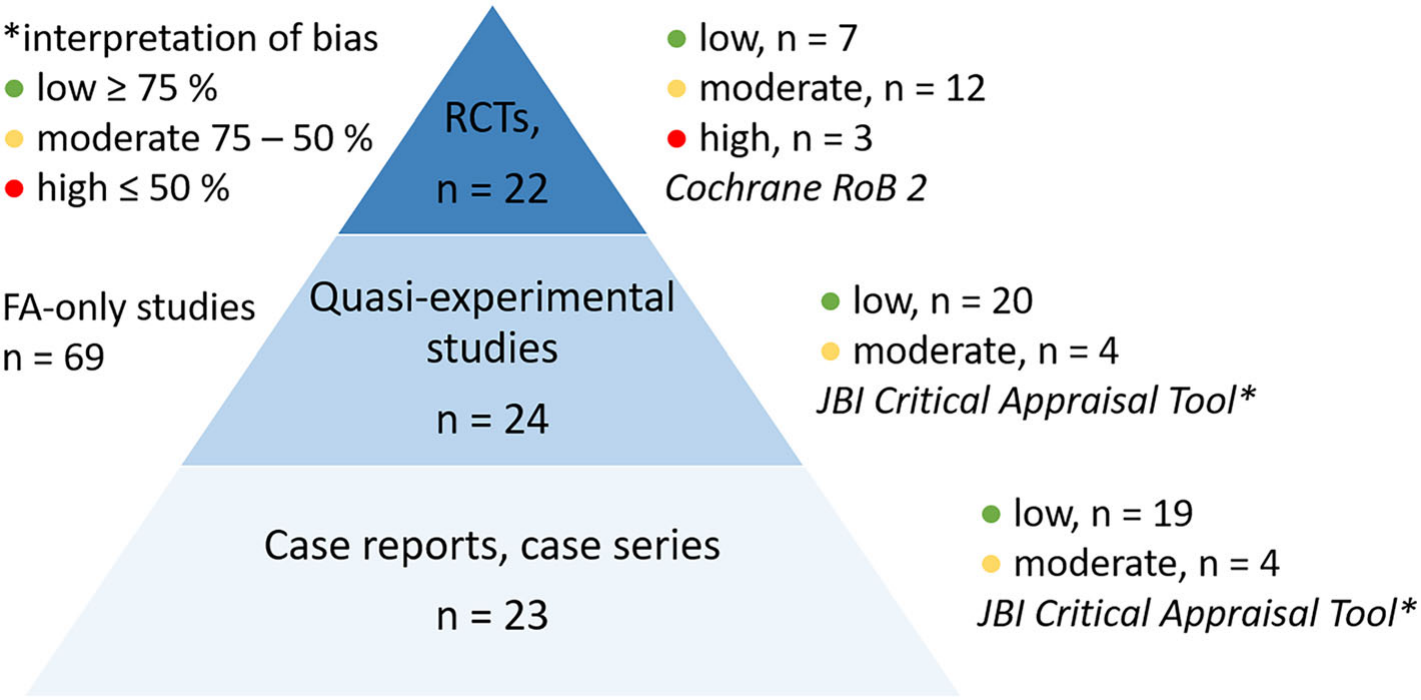
Risk of bias assessment performed for all included studies (n = 69 for Friedreich's ataxia [FA] only) using certain tools depending on the study design. *The answers from JBI Critical Appraisal Tool have been standardized^29^ and presented in the color manner. RCT, randomized controlled trial. [Color figure can be viewed at wileyonlinelibrary.com]

The majority of included studies had a low‐to‐moderate risk of bias. For case reports/series, the bias mostly considered the reporting of adverse events or the clear and full presentation of patients' disease histories. For quasi‐experimental studies, the most considerable bias was related to internal validity because most studies did not include a control group but instead employed a pre‐/post‐intervention design. For RCTs, the most concerning aspects were the randomization process and measurement of outcomes, specifically the blinding of assessors and the assessment of outcomes. In addition, RCTs had small sample sizes.

### Study Population

FA study participants were children (from 9‐year‐old patient in case report), young adults with mean age most commonly around 30 years old, up to 65 years old (individual case report). Mostly, the study population had relatively early FA onset, with different ages and, therefore, different disease durations. In mixed ataxia studies, patients with FA represented only a small percentage of study populations and were generally older than in FA‐only studies. Other ataxias included in these studies were various types of, for example, autosomal dominant cerebellar ataxia such as spinocerebellar ataxias (eg, types 3, 6, 14, 28), idiopathic late‐onset cerebellar ataxia, and multiple system atrophy of cerebellar type, which accordingly varied in their clinical characteristics. Therefore, results cannot be generalized to patients with FA; however, they may provide evidence for potential interventions, which target the ataxia symptoms that are not specific for FA.

### Interventions

#### Pharmacological Interventions

Most pharmacological interventions in FA were disease‐modifying therapies (93.5%), with idebenone (13.0%), interferon‐γ (10.9%), erythropoietin (8.7%), and omaveloxolone (8.7%) as the most commonly investigated medications (Fig. 1C,I). Symptomatic treatment was described in three case reports (twice intrathecal baclofen for spasms and memantine for optic nerve atrophy). For mixed ataxia studies, in pharmacological interventions, three disease‐modifying treatments (vatiquinone and twice riluzole) and one symptomatic treatment (baclofen) were investigated.

As demonstrated in Table 1, 42% of pharmacological RCTs (n = 8) did not show a significant positive effect of the pharmacotherapies, including idebenone (three studies), interferon‐γ, erythropoietin, carbamylated erythropoietin, liraglutide, and luvadaxistat, on the investigated outcome measures.^31^, ^32^, ^33^, ^34^, ^35^, ^36^, ^37^, ^38^ Similarly, no positive trend of the treatment on outcomes was demonstrated in 15% of quasi‐experimental studies on interferon‐γ, tocotrienol, and mixed therapy with darbepoetin alfa, idebenone, and riboflavin.^39^, ^40^, ^41^ Other medications such as idebenone,^42^ deferiprone in high doses^43^ (RCTs), and calcitriol^44^ (quasi‐experimental study) demonstrated significant increase of ataxia severity (measured with Friedreich's Ataxia Rating Scale [FARS] or International Cooperative Ataxia Rating Scale [ICARS], FARS, and FARS ADL [activities of daily living]) or other outcomes (walking speed).

Although erythropoietin reduced oxidative stress and improved brain structures (imaging and laboratory outcomes), it showed no clear benefit in ataxia severity (Scale for the Assessment and Rating of Ataxia [SARA]) in quasi‐experimental studies and no efficacy in an RCT.^36^ Methylprednisolone improved patients' global impression in the overall population but showed no significant improvement in other clinical, laboratory, or patient‐reported outcomes.^45^ In contrast, calcitriol treatment significantly increased frataxin levels but was associated with worsened walking speed.^44^

When observing specific subgroups, pharmacological interventions tended to be more effective in younger, pediatric, and ambulant patients with a shorter disease duration but longer GAA1 repetitions, indicating accelerated disease progression.^34^, ^45^, ^46^, ^47^, ^48^, ^49^ Similarly, better effects were reported for patients with lower initial ataxia severity as measured with ICARS or SARA.^43^, ^50^

#### Nonpharmacological Interventions

Nonpharmacological interventions were rehabilitation, multimodal rehabilitation, occupational therapy, transcranial direct current stimulation, whole‐body vibration therapy, and neuromuscular electrical stimulation (52.2%). Specific target symptoms (47.8%) included cardiomyopathy, hearing, scoliosis, cognitive function, or dysarthria (Fig. 1C), with heart intervention (including heart transplantation, septal myectomy, and catheter ablation; 26.1% of all NP interventions) and rehabilitation (26.1%) being the most common treatments (Fig. 1J).

Nonpharmacological interventions showed more consistent positive outcome tendencies compared with pharmacological interventions, with at least one statistically significant or notable improvement in at least one outcome measure in each study. Conversely, some nonpharmacological interventions also demonstrated deterioration in some outcomes, for example, neurological function after heart transplantation,^51^, ^52^, ^53^ physical performance after the occupational therapy,^54^ or gait and endurance after scoliosis surgery.^55^

When observing subgroups, nonpharmacological interventions tended to be more effective in ambulant, younger patients.^56^

The most common nonpharmacological interventions in studies with mixed ataxia patients were transcranial direct current stimulation (29.4%) and any rehabilitation (23.5%, including multimodal rehabilitation of 5.9%). In addition, other approaches were presented for mixed populations, such as video games (ie, training including body‐controlled video games, often with the use of virtual reality) (17.7%) and noninvasive focal mechanical vibration‐proprioceptive stabilizer (11.8%).

### Alignment of Outcome Measures and Evidence Synthesis According to the ICHOM Criteria

#### Outcome Measures Used

A summary of outcome measures used across all interventions for FA‐only studies is demonstrated in Fig 1G.

Mostly, clinical measures (CL) were used (60 studies in total; 87%), with SARA (24 studies) and FARS (19 studies; FARS Neuro [neurological examination from FARS] 2 studies, mFARS/mFARS Neuro [modified FARS] 9 studies) being the most prevalent measures for ataxia severity. Also, the 9‐HPT (9‐Hole Peg Test) was commonly used for manual dexterity and upper limb coordination (21 studies) and the T25FW (Timed 25‐foot Walk) for mobility and gait assessments (12 studies) (Fig. 1H).

Laboratory measures (L) (25 studies; 36%) mainly included frataxin levels (16 studies), oxidative stress markers (2 studies), iron metabolism (2 studies), and various assessments of cerebellar function (1 study) or muscle function (2 studies). Imaging outcomes (IMG) (25 studies, 36%) included echocardiography (23 studies) imaging of the brain (white or gray matter) or spinal cord (2 studies).

Patient‐reported outcome measures (PROMs) were included in only 37 studies (54%), whereas subjective patient perspectives were gathered in 7 studies (10%) and quality‐of‐life assessments in 18 studies (26%). The most commonly used PROM was the short form health survey with 36 questions (SF‐36; 12 times), modified Fatigue impact scale (MFIS; 6 times), and self‐reported assessment of ADL (5 times). Only four studies (6%) for FA‐only patients included subjective caregiver perspective,^35^, ^55^, ^57^, ^58^ but no caregiver burden or well‐being measures were used.

Administered outcome measures in mixed ataxia studies are demonstrated in Tables S3 and S4 and Figure S2.

#### Addressed Health Domains

Figure 3 categorizes the interventions for patients with FA into seven domains: physical health, overall health, social health, mental health, clinical status, caregiver support, and economic considerations. Each domain demonstrates the number of assessments in FA‐only studies (n = 69 studies, n = 280 assessments in total), in which at least one of the earlier‐mentioned outcomes was addressed. Identified studies most often addressed the physical health domain, with 118 assessments (42.3%) from 54 studies (78.3%). The predominant assessments were cardiac function (23 studies), upper limb and manual dexterity (21 studies), as well as mobility and gait (17 studies). The overall health domain encompassed aspects of health‐related quality of life, which was the most prevalent assessment in this domain (18 studies), as well as ADL (14 studies) and independence (7 studies). Overall health was addressed in 42 assessments (15.1%) from 30 studies (43.5%). Clinical status was the second most commonly addressed domain, with 54 studies (78.3%) and 103 assessments (36.8%). Generic and FA‐specific ataxia severity (35 and 27 studies, respectively) and molecular markers (17) were the most prevalent in this domain.

**FIG. 3:**
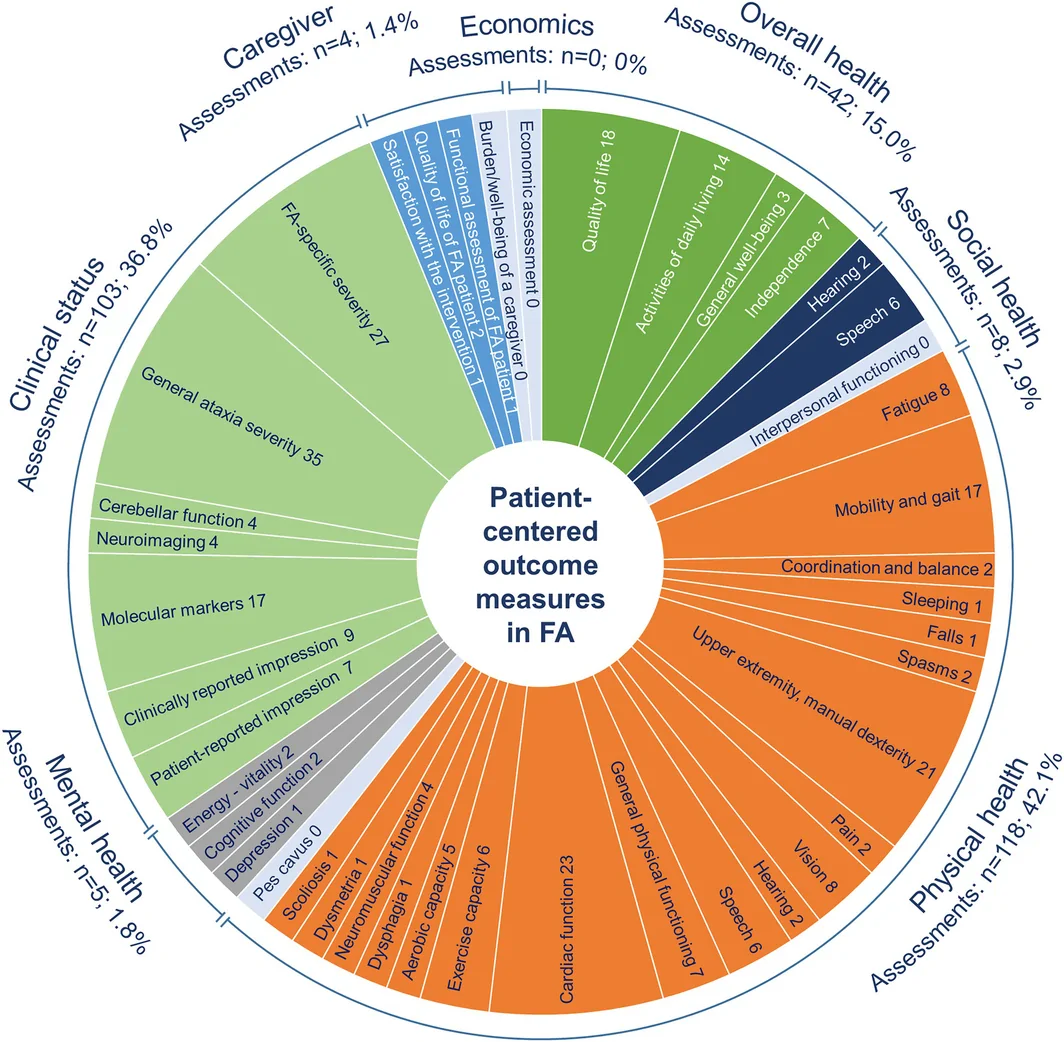
Patient‐centered outcome measures used in the included studies; n = 280 assessments from 69 studies for Friedreich's ataxia (FA)‐only patients and the division in domains and subdomains. [Color figure can be viewed at wileyonlinelibrary.com]

None of the interventions focused on the social health domain in terms of interpersonal functioning. However, hearing (two studies) and speech (six studies) were addressed, which are physical symptoms that directly influence interactions with other people. In total, social health was investigated in only 11.6% of studies (n = 8) (and 2.9% of assessments, n = 8). Mental health was one of the least addressed domains, with four studies (5.8%) and five assessments (1.8%). Although caregivers were involved in four assessments (1.4%) from four studies (5.8%) as proxy ratings, caregiver burden and well‐being were never used. The economic domain—that is, healthcare service utilization, costs, and cost‐effectiveness—was neglected in all of the included studies.

The main targets for patients with mixed ataxia are demonstrated in Figure S4 and Table S12.

#### Effectiveness of Intervention Across Health Domains

Most of the reported outcomes showed no differences, although several demonstrated positive effects, but they were rarely significant. Case reports for most subdomains reported positive effects. Details about domains and the effectiveness of interventions, including study designs and assessments, are available in Table S11.

Even though the physical health domain was most frequently addressed, just 10 significant positive results were reported for the following subdomains: upper extremity and manual dexterity,^59^ mobility and gait (in the child population),^45^ speech,^46^, ^60^ cardiac function,^43^, ^61^ exercise capacity,^50^, ^62^ hearing,^63^ and neuromuscular function.^64^

Included studies did not show a clear improvement in patients' quality of life (overall health domain). The improvement was obtained in specific subdomains, for example, vitality,^58^ physical functioning,^49^ health transition,^36^ or social health.^65^ Moreover, independence significantly improved in one study^54^ and ADL in two studies.^48^, ^66^

Symptoms important for social interactions were effectively addressed. Speech improved significantly after pharmacotherapy^46^, ^60^ and hearing problems after the use of hearing device and cochlear implantation.^63^, ^65^ Speech therapy was more often investigated in mixed ataxia studies.

Several aspects of mental health were addressed in cognitive therapy,^67^ but with no significant positive effect, except for improvements in short‐term memory, cognitive flexibility, and inhibition. There was no effect on depression. Conversely, significant improvements in cognitive function (measured by the Cerebellar Cognitive Affective Syndrome Scale [CCAS‐S]) have been reported, as well as subjective improvements in energy levels and significant improvements in the vitality.^50^, ^68^

Significant improvements in the generic ataxia scale SARA were reported in six studies,^39^, ^50^, ^56^, ^61^, ^69^, ^70^ in the FA‐specific scale FARS in four studies,^57^, ^60^, ^71^, ^72^ and in the mFARS in four studies.^47^, ^48^, ^73^, ^74^

The patient's impression of the disease reported two significant improvements.^45^, ^66^ Frataxin concentration was the most commonly investigated biomarker, with a significant positive effect reported four times.^44^, ^57^, ^58^, ^75^ In addition, reduced oxidative stress was reported once^84^ or twice as a reduced ferritin level, leading also to a reduction in oxidative stress.^75^, ^76^ In neuroimaging, significant improvement in fractional anisotropy in white matter was reported, but with no correlation with SARA or FARS,^32^ as well as a significant increase of gray matter volume in the pulvinar and posterior parietal cortex with a correlation with SARA.^77^

## Discussion

This systematic review identified 90 intervention studies in FA (69 FA only and 21 mixed ataxia) and analyzed key interventions, as well as their effects and alignment with standardized outcome sets of patient‐centered treatment and care. Most studies were small and varied in design and quality, ranging from case reports to RCTs. Overall, pharmacological interventions often showed mixed effects, with omaveloxolone demonstrating more consistent benefits. In contrast, nonpharmacological approaches, such as rehabilitation and particularly multidisciplinary programs, were more frequently reported to improve patient‐relevant outcomes. However, the evidence was based primarily on small case series and potential biases. Subgroup analyses also demonstrated a tendency for better intervention effects in a younger population, groups at earlier stages of FA, with a higher likelihood of faster disease progression.

Clinical Management Guidelines for FA indicated that most intervention recommendations are based on very low levels of evidence, with only a few achieving moderate strength but strong recommendation.^78^ Multimodal rehabilitation has the strongest support for neurological components and fatigue, whereas cardiac interventions (eg, transplantation or heart devices), with limited evidence, may be considered on an individual basis in patients with impaired cardiac function. In our review, (multimodal) rehabilitation and heart interventions were the most prevalent nonpharmacological interventions, highlighting the importance of these approaches. Heart and muscle function are interdependent because the heart supplies oxygen and nutrients essential for muscle performance, while muscle activity supports cardiovascular health. Although movement‐related symptoms are often the most disruptive in everyday life in FA,^15^, ^38^, ^79^ cardiac complications may be less apparent to patients yet represent a significant life‐threatening risk^38^; therefore, it is crucial to address these issues. Presented findings highlight both the potential of specific therapies and the substantial gaps in the existing evidence base of interventional research in FA. Future research should focus on strengthening the evidence base for interventions in FA, with particular attention to their impact on patients' daily lives.

Nonpharmacological interventions were more patient and symptom oriented and more frequently reported positive outcomes because they typically target specific functional impairments. They may also involve more active engagement of patients; for example, physical activity can improve energy and motivation,^80^ or social interaction may contribute to a better overall effect.^81^ However, these findings should be interpreted with caution given the frequent use of case reports and quasi‐experimental designs, which are associated with a higher risk of bias. Pharmacological interventions often aim either to stop or at least to slow the disease progression; therefore, the positive effect may not be directly visible. Although placebo effects in neurodegenerative ataxias may reach up to 32% showing improvement in at least one ataxia rating scale,^82^ the successful blinding of the RCT may minimize the expectation‐related bias.^54^

Many medications proved ineffective; some improved only certain outcomes. Clear evidence for effective disease‐modifying therapies remains lacking. Current disease‐modifying therapeutic strategies in FA aim to increase frataxin expression or replacement, improve mitochondrial function, reduce oxidative stress, modulate downstream metabolic pathways, and provide neuroprotection.^83^ Targeting multiple pathogenic mechanisms may enable complementary or combination approaches and support the development of more individualized treatments. Several mixed effects were reported for pharmacological interventions, where laboratory or imaging outcomes were improved, with no effect on clinical measures or PROMs. This mismatch between biological signals and clinical benefit underscores the complexity of FA and may indicate that an intervention influences underlying biological processes without yet producing detectable clinical or patient‐perceived benefits. Clinically meaningful changes in patient‐relevant outcomes may evolve gradually, requiring longer treatment and observation periods to capture clinical improvements. Understanding the mechanisms underlying therapeutic effects is crucial for optimizing interventions, establishing causal pathways, and translating promising findings into effective clinical treatments. Future research should therefore integrate the variety of outcomes, including laboratory, clinical, and patient‐reported outcomes, in a complementary manner,^84^, ^85^ to better capture the association between outcomes and the full spectrum of treatment effects, ensuring that advances translate into meaningful benefits for patients.

Idebenone was the most frequently investigated medication; however, the results were inconclusive. The clinical trial IONIA in the pediatric population showed that idebenone did not improve cardiac function,^33^ whereas in the open‐label extension, potential therapeutic benefit to stabilize the disease progression and to improve specific symptoms, for example, fine motor skills and speech, was shown.^42^ So far, omaveloxolone in the MOXIe trial demonstrated more consistent positive effects in terms of neurological progression assessed with mFARS.^47^, ^48^ Short‐term effects and the long‐term slowing of the progression in comparison with natural progression (almost 3 years) were demonstrated.^86^ Greater improvement was reported for the patients without pes cavus and those who started the treatment earlier, suggesting its efficacy at the less severe FA stages.^73^ Future research should therefore prioritize identifying responsive populations and specific symptoms to support more personalized and effective therapeutic strategies.

Outcome measures were heterogeneous across studies in this review, with clinical scales being most common and PROMs less prevalent. PROMs were reported in just more than half of the FA‐only studies, highlighting the need for possible greater patient involvement and the development of standardized, responsive measures.^87^, ^88^, ^89^ Several recommendations have been made regarding outcome measures used in FA research (eg, core measure instruments, including clinical scales such as SARA, 9‐HPT, T25FW, and mFARS^113^, ^114^) to facilitate a comprehensive evaluation^119^ and good comparability between studies. It is crucial to use standardized outcome measures and reporting, with consistent thresholds for clinical relevance. Although the minimal clinically important difference (MCID) is essential for determining whether observed changes are clinically meaningful, currently, no disease‐specific MCID values are available for FA.^90^ Standard outcome measures in FA should capture clinically meaningful aspects of the disease, including neurological function and disease progression, health‐related quality of life, mental well‐being, and patient perspective. Selection should prioritize validated, responsive measures that are relevant to patients and aligned with the specific objectives of the intervention. This will enable consistent reporting and a clearer overview of interventional outcomes, ultimately leading to improved development of new therapies.

Physical health was the most frequently addressed domain, which demonstrates that FA is primarily considered a movement disorder. Holistic care of patients with FA requires recognizing it as a multidimensional disease and addressing more health domains, for example, psychosocial health. FA considerably affects mental health, with literature reporting the prevalence of depression in FA up to 40%^91^, ^92^ or other emotional issues (eg, decreased motivation, feeling fearful about the future, social isolation).^14^, ^15^ Deterioration in cognitive functions (eg, in emotional processing, social cognition) and CCAS has also been reported.^13^, ^93^, ^94^, ^95^ In addition, a loss of ambulation or transition between mobility devices as the disease progresses may directly influence mental health.^96^, ^97^ Patients with FA are often stigmatized in social life because of symptoms of movement and speech impairments resembling alcohol or drug use, which may further exacerbate the social isolation and mental health issues.^98^ Although the gathered evidence level is low, the Clinical Management Guidelines strongly recommend counseling or therapy in case of depression and anxiety, as well as lifestyle changes (such as exercise, diet, social activities) or pharmacotherapy.^78^ This indicates that psychosocial health is important and should be addressed more thoroughly to gather further evidence. Future interventional studies should therefore expand beyond physical function to systematically address psychosocial and cognitive domains, ensuring a holistic and patient‐centered approach.

In our review, we found no studies that considered caregivers' burden or the impact of the availability of a caregiver. The perspective of caregivers of patients with FA has been largely neglected but should be included in research and interventions. Caregivers play a central role in supporting individuals with FA, yet often experience substantial physical, emotional, social, and financial burden, which can negatively affect their own health and caregiving capacity.^137^ Supporting caregivers may therefore not only improve caregiver well‐being but also indirectly enhance patient care.

FA is a tremendous economic and societal burden.^99^, ^100^, ^101^, ^102^ The economic and health resource outcomes in FA, including healthcare utilization, informal care, caregiver productivity losses, and adverse health events, constitute a gap in the current research that has only recently begun to be addressed.^103^, ^104^ Therefore, future interventional studies should also consider incorporating such outcomes to capture the full societal and economic impact of FA and to inform sustainable healthcare planning and policy. Given the complexity of FA, comprehensive evaluation, including clinical, patient‐reported, caregiver, health, economic, and psychosocial outcomes, may yield the best results.

### Limitations

Although the searching strategy was broad and included several interventions, we cannot ensure that all existing interventions were included in this review. Studies with various designs and small sample sizes have been included, exhibiting a low‐to‐moderate bias. These aspects may significantly impact the level of evidence supporting the presented outcomes and limit their robustness and generalizability.

### Implications for Future Research

Future interventional studies should use multicenter and robust study designs that incorporate a variety of clinical, patient‐reported, and health system–relevant end points. The literature has indicated that patients with FA are motivated to participate in studies, which could help facilitate the setup of larger studies.^105^ However, common barriers include the ethical allocation to a placebo group, treatment side effects, and the logistics of travel and participation.^105^ Innovative, remote, or app‐based interventional designs might overcome these barriers by reducing patient burden, increasing accessibility, and enabling more flexible participation in FA trials.

Interventions implemented in mixed ataxia populations can be further investigated in FA‐only populations to confirm efficacy and reduce heterogeneity across distinct disease entities. Several nonpharmacological interventions are currently ongoing that target various issues, for example, swallowing and respiratory function,^106^ standing balance,^107^ speech therapy involving behavioral intervention,^108^ biofeedback therapy,^109^ or NAD+ (Nicotinamide adenine dinucleotide (oxidized form)) and exercise.^110^ Nonpharmacological interventions must be investigated using more robust trial designs because controlled designs are still underrepresented. Similarly, several ongoing clinical studies at various stages are investigating new medications for FA.^111^ Omaveloxolone has been approved by the US Food and Drug Administration (FDA) and European Medicines Agency (EMA), since 2023 and 2024, respectively,^18^, ^19^ as the first FA disease‐modifying treatment for patients aged 16 years and older. Recently, vatiquinone, another candidate drug for FA treatment, was evaluated by the FDA; however, further evidence is still needed to prove its efficacy.^112^ All presented studies and protocols show that there is constant development in the research on interventions for FA management, and new approaches are being tested, providing hope for patients with FA to gain access to another new and effective therapy.

## Conclusion

This review summarizes current research on pharmacological and nonpharmacological interventions in FA and highlights symptoms and health domains that remain underexplored. Pharmacological interventions as disease‐modifying therapies targeting pathophysiology of FA have shown mostly limited benefits to date, with omaveloxolone exhibiting considerable effectiveness. Omaveloxolone, although not yet widely available, may potentially become part of standard care in the future. Nonpharmacological approaches showed promise in symptom‐driven research. Research has focused primarily on physical symptoms affecting movement, whereas many comorbidities, psychosocial health, caregiver burden, well‐being, and economic aspects remain poorly studied. These gaps underscore the urgent need for further research. Translating findings into effective therapies requires rigorous research and multidisciplinary approaches that integrate all relevant health domains and involve both patients and caregivers.

## Author Roles

(1) Research Project: A. Design, B. Organization, C. Execution; (2) Manuscript Preparation: A. Drafting Initial Version, B. Editing Final Version.

D.S.: 1A, 1B, 1C, 2A, 2B.

M.B.: 1A, 1C, 2B.

A.I.: 2B.

M.G.‐E.: 1A, 2B.

T.K.: 1A, 2B.

J.F.: 2B.

B.M.: 1A, 1C, 2B.

## Financial Disclosures and Conflicts of Interest

J.F. has received consultancy honoraria from Vico Therapeutics and Biogen. M.G.‐E. has received honoraria from Biogen and travel support from Apario and the Ataxia Global Initiative. B.M. has received consultancy honoraria from Biogen, Lilly, and EuroQol. T.K. has received, within the last 24 months, consulting fees from Arrowhead, Bristol‐Myers Squibb, and UCB. All funding is unrelated to this study. Author disclosures are available in the Supporting Information.

## Supporting information


**Figure S1.** PRISMA chart: the process of studies selection.^1^
**Figure S2**. Characteristics of included studies for mixed ataxia patients. (A) Study populations from all included publications: n = 69 studies for FA‐only patients, n = 21 for mixed ataxia patients. (B) Interventions types: n = 4 pharmacological interventions (P), n = 17 nonpharmacological interventions (NP). (C) Timeline for pharmacological and nonpharmacological studies. (D) Study designs of included studies regarding an intervention type and total number of studies. (E) Outcome measures used in included studies: CL, clinical; L, laboratory; PROM, patient‐reported outcome measure (incl. subjective and quality of life); IMG, imaging. (F) The prevalence of core outcome measures used in included studies: SARA, Scale for the Assessment and Rating of Ataxia; 9‐HPT, 9‐Hole Peg Test; T25FW, Timed 25‐Foot Walk; mFARS, modified Friedreich's Ataxia Rating Scale.^24^, ^25^ (G) Types of pharmacological interventions, n = 4: disease‐modifying, n = 3; *symptomatic, n = 1. (H) Types of nonpharmacological interventions, n = 17.
**Figure S3**. Risk of bias assessment performed for all included studies for mixed ataxia population (n = 21) using certain tools depending on the study design. *The answers from JBI Critical Appraisal Tool have been standardized^28^ and presented in the color manner.
**Figure S4**. Patient‐centered outcome measures used in the included studies; n = 71 assessments from 21 studies for mixed ataxia patients and the division in domains and subdomains.
**Table S1**. Inclusion and exclusion criteria of studies in the presented review according to PICOS methodology.^2^
**Table S2**. Final search strategy applied in the presented review.
**Table S3**. Characteristics of included studies with pharmacological interventions for mixed ataxia patients, n = 4 studies.
**Table S4**. Characteristics of included studies with non‐pharmacological interventions in mixed ataxia patients, n = 17 studies.
**Table S5**. Risk of bias assessment for randomized controlled trials in FA‐only population, n = 22, Cochrane RoB‐2.^26^
**Table S6**. Risk of bias assessment for quasi‐experimental studies in FA‐only population, n = 24, JBI Critical Appraisal Tool.^27^ The answers have been standardized and presented in the color manner according to the literature^28^: ≥75% “Yes” answers = low bias–high quality; 50–74% = moderate bias; <50% = high bias.
**Table S7**. Risk of bias assessment performed for case reports for FA‐only population, n = 21.^29^ The answers have been standardized and presented in the color manner according to the literature^28^: ≥75% “Yes” answers = low bias‐high quality; 50–74% = moderate bias; <50% = high bias.
**Table S8**. Risk of bias assessment performed for case series, n = 2 for FA‐only population.^30^ The answers have been standardized and presented in the color manner according to the literature^28^: ≥75% “Yes” answers = low bias‐high quality; 50–74% = moderate bias; <50% = high bias.
**Table S9**. Risk of bias assessment for randomized controlled trials in mixed ataxia population, n = 10, Cochrane RoB‐2.^26^
**Table S10**. Risk of bias assessment for quasi‐experimental studies in mixed ataxia population, n = 11, JBI Critical Appraisal Tool.^27^ The answers have been standardized and presented in the color manner according to the literature^28^: ≥75% “Yes” answers = low bias‐high quality; 50–74% = moderate bias; <50% = high bias.
**Table S11**. Patient‐centered outcome measures and their effects on certain health domains and subdomains in FA‐only studies, n = 280 assessments from 69 studies.
**Table S12**. Patient‐centered outcome measures and their effects on certain health domains and subdomains in mixed ataxia studies, n = 71 assessments from 21 studies.

**Data S1**. Supporting Information.

## Data Availability

The data that supports the findings of this study are available in the supplementary material of this article.

## References

[1] Vankan P . Prevalence gradients of Friedreich's ataxia and R1b haplotype in Europe co‐localize, suggesting a common Palaeolithic origin in the Franco‐Cantabrian ice age refuge. J Neurochem 2013;126(Suppl 1):11–20. 10.1111/jnc.12215 23859338

[2] Harding AE . FRIEDREICH'S ataxia: A clinical and genetic study of 90 families with an analysis of early diagnostic criteria and intrafamilial clustering of clinical features. Brain 1981;104:589–620. 10.1093/brain/104.3.589 7272714

[3] Lynch DR , Farmer JM , Balcer LJ , Wilson RB . Friedreich ataxia: effects of genetic understanding on clinical evaluation and therapy. Arch Neurol 2002;59:743–747. 10.1001/archneur.59.5.743 12020255

[4] Indelicato E , Nachbauer W , Eigentler A , et al. Onset features and time to diagnosis in Friedreich's ataxia. Orphanet J Rare Dis 2020;15(1):198. 10.1186/s13023-020-01475-9 PMC7397644 32746884

[5] Patel M , Isaacs CJ , Seyer L , et al. Progression of Friedreich ataxia: quantitative characterization over 5 years. Ann Clin Transl Neurol 2016;3(9):684–694. 10.1002/acn3.332 PMC5018581 27648458

[6] Salvucci A , Qian C , Powell L , Lynch D , Vasco G , Johnston K , Tomazos I . CO105 A retrospective study characterizing age at loss of ambulation among patients with Friedreich ataxia using health administrative claims data in the United States. Value Health 2023;26(12):S33–S34.

[7] Campuzano V , Montermini L , Moltò MD , et al. Friedreich's ataxia: autosomal recessive Disase caused by an intronic GAA triplet repeat expansion. Science 1996;271:1423–1427. 10.1126/science.271.5254.1423 8596916

[8] Cook A , Giunti P . Friedreich's ataxia: clinical features, pathogenesis and management. Br Med Bull 2017;124(1):19–30. 10.1093/bmb/ldx034 PMC5862303 29053830

[9] Naeije G , Schulz JB , Corben LA . The cognitive profile of Friedreich ataxia: a systematic review and meta‐analysis. BMC Neurol 2022;22(1):97. 10.1186/s12883-022-02615-3 PMC8928653 35300598

[10] Reetz K , Dogan I , Hilgers RD , et al. Progression characteristics of the European Friedreich's ataxia consortium for translational studies (EFACTS): a 4‐year cohort study. The Lancet Neurology 2021;20(5):362–372. 10.1016/S1474-4422(21)00027-2 33770527

[11] Fichera M , Castaldo A , Mongelli A , Marchini G , Gellera C , Nanetti L , Mariotti C . Comorbidities in Friedreich ataxia: incidence and manifestations from early to advanced disease stages. Neurol Sci 2022;43(12):6831–6838. 10.1007/s10072-022-06360-w 36053339

[12] Nieto A , Hernández‐Torres A , Pérez‐Flores J , Montón F . Depressive symptoms in Friedreich ataxia. Int J Clin Health Psychol 2018;18(1):18–26. 10.1016/j.ijchp.2017.11.004 PMC6220911 30487906

[13] Hernandez‐Torres A , Montón F , Hess Medler S , Nóbrega É , Nieto A . Longitudinal study of cognitive functioning in Friedreich's ataxia. J Int Neuropsychol Soc 2021;27(4):343–350. 10.1017/S1355617720000958 33050966

[14] Karamazovova S , Stovickova L , Jester DJ , et al. Exploring neuropsychiatric symptoms in Friedreich ataxia. Sci Rep 2024;14(1):29076. 10.1038/s41598-024-80258-9 PMC11585572 39580547

[15] Lowit A , Greenfield J , Cutting E , Wallis R , Hadjivassiliou M . Symptom burden of people with progressive ataxia, and its wider impact on their friends and relatives: a cross‐sectional study. AMRC Open Res 2021;3:28. 10.12688/amrcopenres.13036.1 PMC11064976 38708068

[16] Xiong E , Lynch AE , Corben LA , et al. Health related quality of life in Friedreich ataxia in a large heterogeneous cohort. J Neurol Sci 2020;410:116642. 10.1016/j.jns.2019.116642 31901720

[17] European Reference Network . Consensus guideline für klinisches management der Friedreich's Ataxie. Luxembourg: Publications Office of the European Union, 2019.

[18] Administration, U.S.F.D . FDA approves first treatment for Friedreich's ataxia; 2023. Accessed May 12, 2025. https://www.fda.gov/drugs/news-events-human-drugs/fda-approves-first-treatment-friedreichs-ataxia.

[19] Biogen . Biogen received european commission approval for SKYCLARYS® (omaveloxolone), the first therapy to treat Friedreich's Ataxia; 2024. Accessed May 12, 2025. https://investors.biogen.com/news-releases/news-release-details/biogen-received-european-commission-approval-skyclarysr.

[20] Biogen Netherlands B.V . Summary of product characteristics Skyclarys.

[21] Umrao A , Pahuja M , Chatterjee NS . Safety and efficacy of omaveloxolone v/s placebo for the treatment of Friedreich's ataxia in patients aged more than 16 years: a systematic review. Orphanet J Rare Dis 2024;19(1):495. 10.1186/s13023-024-03474-6 PMC11684145 39736600

[22] Higgins JPT , T J , Chandler J , Cumpston M , Li T , Page MJ , Welch VA . Cochrane Handbook for Systematic Reviews of Interventions Version 6.5 (Updated August 2024). London, UK: Cochrane; 2024.

[23] Moher D , Liberati A , Tetzlaff J , Altman DG , The PRISMA Group . Preferred reporting items for systematic reviews and meta‐analyses: the PRISMA statement. PLoS Med 2009;6(7):e1000097. 10.1371/journal.pmed.1000097 PMC2707599 19621072

[24] PROSPERO home page; Accessed June 19, 2025. https://www.crd.york.ac.uk/PROSPERO/.

[25] Ouzzani M , Hammady H , Fedorowicz Z , Elmagarmid A . Rayyan‐a web and mobile app for systematic reviews. Syst Rev 2016;5(1):210. 10.1186/s13643-016-0384-4 PMC5139140 27919275

[26] Sterne JAC , Savović J , Page MJ , et al. RoB 2: a revised tool for assessing risk of bias in randomised trials. BMJ 2019;366:l4898. 10.1136/bmj.l4898 31462531

[27] Barker TH , Habibi N , Aromataris E , et al. The revised JBI critical appraisal tool for the assessment of risk of bias for quasi‐experimental studies. JBI Evid Synth 2024;22(3):378–388. 10.11124/JBIES-23-00268 38287725

[28] Munn Z , Barker TH , Moola S , et al. Methodological quality of case series studies: an introduction to the JBI critical appraisal tool. JBI Evid Synth 2020;18(10):2127–2133. 10.11124/JBISRIR-D-19-00099 33038125

[29] de Smalen AW , Chan ZX , Abreu Lopes C , Vanore M , Loganathan T , Pocock NS . Developing an evidence assessment framework and appraising the academic literature on migrant health in Malaysia: a scoping review. BMJ Open 2021;11(1):e041379. 10.1136/bmjopen-2020-041379 PMC7813391 33462099

[30] Page MJ , McKenzie JE , Bossuyt PM , et al. The PRISMA 2020 statement: an updated guideline for reporting systematic reviews. BMJ 2021;372:n71. 10.1136/bmj.n71 PMC8005924 33782057

[31] Boesch S , Nachbauer W , Mariotti C , et al. Safety and tolerability of carbamylated erythropoietin in Friedreich's ataxia. Mov Disord 2014;29(7):935–939. 10.1002/mds.25836 24515352

[32] Drinkard BE , Keyser RE , Paul SM , Arena R , Plehn JF , Yanovski JA , di Prospero NA . Exercise capacity and idebenone intervention in children and adolescents with Friedreich ataxia. Arch Phys Med Rehabil 2010;91(7):1044–1050. 10.1016/j.apmr.2010.04.007 PMC2995920 20599042

[33] Lagedrost SJ , Sutton MSJ , Cohen MS , et al. Idebenone in Friedreich ataxia cardiomyopathy‐results from a 6‐month phase III study (IONIA). Am Heart J 2011;161(3):639–645 e1. 10.1016/j.ahj.2010.10.038 21392622

[34] Lynch DR , Perlman SL , Meier T . A phase 3, double‐blind, placebo‐controlled trial of Idebenone in Friedreich ataxia. Arch Neurol 2010;67(8):941–947. 10.1001/archneurol.2010.168 20697044

[35] Lynch DR , Hauser L , McCormick A , et al. Randomized, double‐blind, placebo‐controlled study of interferon‐gamma 1b in Friedreich ataxia. Ann Clin Transl Neurol 2019;6(3):546–553. 10.1002/acn3.731 PMC6414489 30911578

[36] Mariotti C , Fancellu R , Caldarazzo S , et al. Erythropoietin in Friedreich ataxia: No effect on frataxin in a randomized controlled trial. Mov Disord 2012;27(3):446–449. 10.1002/mds.24066 22411849

[37] Pandolfo M , Reetz K , Darling A , et al. Efficacy and safety of Leriglitazone in patients with Friedreich ataxia: A phase 2 double‐blind, randomized controlled trial (FRAMES). Neurol Genet 2022;8(6):e200034. 10.1212/NXG.0000000000200034 PMC9747094 36524101

[38] Wang H , Norton J , Xu L , et al. Results of a randomized double‐blind study evaluating luvadaxistat in adults with Friedreich ataxia. Ann Clin Transl Neurol 2021;8(6):1343–1352. 10.1002/acn3.51373 PMC8164851 34018342

[39] Arpa J , Sanz‐Gallego I , Rodríguez‐de‐Rivera FJ , et al. Triple therapy with darbepoetin alfa, idebenone, and riboflavin in Friedreich's ataxia: an open‐label trial. Cerebellum 2013;12(5):713–720. 10.1007/s12311-013-0482-y 23625326

[40] Bolotta A , Pini A , Abruzzo PM , et al. Effects of tocotrienol supplementation in Friedreich's ataxia: A model of oxidative stress pathology. Exp Biol Med (Maywood) 2020;245(3):201–212. 10.1177/1535370219890873 PMC7045332 31795754

[41] Marcotulli C , Fortuni S , Arcuri G , et al. GIFT‐1, a phase IIa clinical trial to test the safety and efficacy of IFNgamma administration in FRDA patients. Neurol Sci 2016;37(3):361–364. 10.1007/s10072-015-2427-3 26621361

[42] Meier T , Perlman SL , Rummey C , Coppard NJ , Lynch DR . Assessment of neurological efficacy of idebenone in pediatric patients with Friedreich's ataxia: data from a 6‐month controlled study followed by a 12‐month open‐label extension study. J Neurol 2012;259(2):284–291. 10.1007/s00415-011-6174-y 21779958

[43] Pandolfo M , Arpa J , Delatycki MB , et al. Deferiprone in Friedreich ataxia: a 6‐month randomized controlled trial. Ann Neurol 2014;76(4):509–521. 10.1002/ana.24248 25112865

[44] Alemany‐Perna B , Tamarit J , Cabiscol E , et al. Calcitriol treatment is safe and increases frataxin levels in Friedreich ataxia patients. Mov Disord 2024;39(7):1099–1108. 10.1002/mds.29808 38696306

[45] Patel M , Schadt K , McCormick A , Isaacs C , Dong YN , Lynch DR . Open‐label pilot study of oral methylprednisolone for the treatment of patients with friedreich ataxia. Muscle Nerve 2019;60(5):571–575. 10.1002/mus.26610 31206761

[46] Cook A , Boesch S , Heck S , et al. Patient‐reported outcomes in Friedreich's ataxia after withdrawal from idebenone. Acta Neurol Scand 2019;139(6):533–539. 10.1111/ane.13088 30887496

[47] Lynch DR , Farmer J , Hauser L , et al. Safety, pharmacodynamics, and potential benefit of omaveloxolone in Friedreich ataxia. Ann Clin Transl Neurol 2019;6(1):15–26. 10.1002/acn3.660 PMC6331199 30656180

[48] Lynch DR , Chin MP , Delatycki MB , et al. Safety and efficacy of Omaveloxolone in Friedreich ataxia (MOXIe study). Ann Neurol 2021;89(2):212–225. 10.1002/ana.25934 PMC7894504 33068037

[49] Lynch DR , Chin MP , Delatycki MB , et al. Safety and efficacy of Omaveloxolone in Friedreich ataxia (MOXIe study)‐correction. Ann Neurol 2021;89(2):212–225. 10.1002/ana.25934 PMC7894504 33068037

[50] Paparella G , Stragà C , Pesenti N , et al. A pilot phase 2 randomized trial to evaluate the safety and potential efficacy of etravirine in Friedreich ataxia patients. Children (Basel) 2024;11(8):958. 10.3390/children11080958 PMC11352957 39201893

[51] Yoon G , Soman T , Wilson J , et al. Cardiac transplantation in Friedreich ataxia. J Child Neurol 2012;27(9):1193–1196. 10.1177/0883073812448229 PMC3671892 22752490

[52] Ivak P , Zumrova A , Netuka I . Friedreich's ataxia and advanced heart failure: an ethical conundrum in decision‐making. J Heart Lung Transplant 2016;35(9):1144–1145. 10.1016/j.healun.2016.06.021 27469014

[53] McCormick A , Shinnick J , Schadt K , et al. Cardiac transplantation in Friedreich ataxia: extended follow‐up. J Neurol Sci 2017;375:471–473. 10.1016/j.jns.2017.01.027 28109580

[54] Ciancarelli I , Cofini V , Carolei A . Disabilità e terapia occupazionale nei pazienti con atassia di Friedreich. G Ital Med Lav Ergon 2011;33(2):201–204. 21796925

[55] Leighton J , Howard J , El‐Hawary R . Posterior spinal fusion for Friedreich ataxia‐related scoliosis in twin girls, A case report. J Bone Joint Surg Case Connector 2013;3(1):e39. 10.2106/JBJS.CC.L.00056 29252233

[56] Paparella G , Stragà C , Vavla M , et al. Effectiveness of rehabilitation intervention in persons with Friedreich ataxia. Front Neurol 2023;14:1270296. 10.3389/fneur.2023.1270296 PMC10653317 38020600

[57] Seyer L , Greeley N , Foerster D , et al. Open‐label pilot study of interferon gamma‐1b in Friedreich ataxia. Acta Neurol Scand 2015;132(1):7–15. 10.1111/ane.12337 25335475

[58] Libri V , Yandim C , Athanasopoulos S , et al. Epigenetic and neurological effects and safety of high‐dose nicotinamide in patients with Friedreich's ataxia: an exploratory, open‐label, dose‐escalation study. Lancet 2014;384(9942):504–513. 10.1016/S0140-6736(14)60382-2 24794816

[59] Sacca F , Puorro G , Marsili A , et al. Long‐term effect of epoetin alfa on clinical and biochemical markers in friedreich ataxia. Mov Disord 2016;31(5):734–741. 10.1002/mds.26552 26879839

[60] Yiu EM , Tai G , Peverill RE , et al. An open‐label trial in Friedreich ataxia suggests clinical benefit with high‐dose resveratrol, without effect on frataxin levels. J Neurol 2015;262(5):1344–1353. 10.1007/s00415-015-7719-2 25845763

[61] Vavla M , D'Angelo M , Arrigoni F , et al. Safety and efficacy of interferon gamma in friedreich's ataxia. Mov Disord 2020;35(2):370–371. 10.1002/mds.27979 31930551

[62] Zesiewicz T , Heerinckx F , de Jager R , Omidvar O , Kilpatrick M , Shaw J , Shchepinov MS . Randomized, clinical trial of RT001: early signals of efficacy in Friedreich's ataxia. Mov Disord 2018;33(6):1000–1005. 10.1002/mds.27353 29624723

[63] Rance G , Corben LA , du Bourg E , King A , Delatycki MB . Successful treatment of auditory perceptual disorder in individuals with Friedreich ataxia. Neuroscience 2010;171(2):552–555. 10.1016/j.neuroscience.2010.09.013 20849937

[64] Herrero AJ , Martín J , Martín T , García‐López D , Garatachea N , Jiménez B , Marín PJ . Whole‐body vibration alters blood flow velocity and neuromuscular activity in Friedreich's ataxia. Clin Physiol Funct Imaging 2011;31(2):139–144. 10.1111/j.1475-097X.2010.00992.x 21078065

[65] Frewin B , Chung M , Donnelly N . Bilateral cochlear implantation in Friedreich's ataxia: A case study. Cochlear Implants Int 2013;14(5):287–290. 10.1179/1754762813Y.0000000029 23485447

[66] Milne SC , Corben LA , Roberts M , et al. Can rehabilitation improve the health and well‐being in Friedreich's ataxia: a randomized controlled trial? Clin Rehabil 2018;32(5):630–643. 10.1177/0269215517736903 29072092

[67] Ciancarelli I , Cofini V , Carolei A . Evaluation of neuropsychological functions in patients with Friedreich ataxia before and after cognitive therapy. Funct Neurol 2010;25(2):81–85. 20923605

[68] Garcia Ron A , Rodriguez Mesa M . Compassionate use of human recombinant insulin‐like growth factor‐1 therapy in Friedreich's ataxia. Neurología (English Edition) 2020;35(2):140–143. 10.1016/j.nrl.2017.11.004 29338935

[69] Costantini A , Laureti T , Pala MI , et al. Long‐term treatment with thiamine as possible medical therapy for Friedreich ataxia. J Neurol 2016;263(11):2170–2178. 10.1007/s00415-016-8244-7 27488863

[70] Naeije G , Rovai A , Destrebecq V , Trotta N , de Tiège X . Anodal cerebellar transcranial direct current stimulation reduces motor and cognitive symptoms in Friedreich's ataxia: A randomized, *sham‐controlled trial* . Mov Disord 2023;38(8):1443–1450. 10.1002/mds.29453 37310043

[71] Lynch DR , Willi SM , Wilson RB , et al. A0001 in Friedreich ataxia: biochemical characterization and effects in a clinical trial. Mov Disord 2012;27(8):1026–1033. 10.1002/mds.25058 22744651

[72] Zesiewicz T , Salemi JL , Perlman S , et al. Double‐blind, randomized and controlled trial of EPI‐743 in Friedreich's ataxia. Neurodegener Dis Manag 2018;8(4):233–242. 10.2217/nmt-2018-0013 30051753

[73] Lynch DR , Chin MP , Boesch S , et al. Efficacy of Omaveloxolone in Friedreich's ataxia: delayed‐start analysis of the MOXIe extension. Mov Disord 2023;38(2):313–320. 10.1002/mds.29286 36444905

[74] Lynch DR , Mathews KD , Perlman S , et al. Double blind trial of a deuterated form of linoleic acid (RT001) in Friedreich ataxia. J Neurol 2023;270(3):1615–1623. 10.1007/s00415-022-11501-4 36462055

[75] Nachbauer W , Hering S , Seifert M , et al. Effects of erythropoietin on frataxin levels and mitochondrial function in Friedreich ataxia–a dose‐response trial. Cerebellum 2011;10(4):763–769. 10.1007/s12311-011-0287-9 21597884

[76] Sacca F , Piro R , de Michele G , et al. Epoetin alfa increases frataxin production in Friedreich's ataxia without affecting hematocrit. Mov Disord 2011;26(4):739–742. 10.1002/mds.23435 21506154

[77] Santner W , Schocke M , Boesch S , Nachbauer W , Egger K . A longitudinal VBM study monitoring treatment with erythropoietin in patients with Friedreich ataxia. Acta Radiol Short Rep 2014;3(4):2047981614531573. 10.1177/2047981614531573 PMC4184378 25298866

[78] Corben LA , Collins V , Milne S , et al. Clinical management guidelines for Friedreich ataxia: best practice in rare diseases. Orphanet J Rare Dis 2022;17(1):415. 10.1186/s13023-022-02568-3 PMC9652828 36371255

[79] Seabury J , Alexandrou D , Dilek N , et al. Patient‐reported impact of symptoms in Friedreich ataxia. Neurology 2023;100(8):e808–e821. 10.1212/WNL.0000000000201598 PMC9984224 36443012

[80] Lindheimer JB , O'Connor PJ , Dishman RK . Quantifying the placebo effect in psychological outcomes of exercise training: a meta‐analysis of randomized trials. Sports Med 2015;45(5):693–711. 10.1007/s40279-015-0303-1 25762083

[81] Cassidy E , Naylor S , Reynolds F . The meanings of physiotherapy and exercise for people living with progressive cerebellar ataxia: an interpretative phenomenological analysis. Disabil Rehabil 2018;40(8):894–904. 10.1080/09638288.2016.1277400 28166646

[82] Choi JH , Shin C , Kim HJ , Jeon B . Placebo response in degenerative cerebellar ataxias: a descriptive review of randomized, placebo‐controlled trials. J Neurol 2022;269(1):62–71. 10.1007/s00415-020-10306-7 33219422

[83] Reetz K , Lischewski SA , Dogan I , et al. Friedreich's ataxia‐a rare multisystem disease. Lancet Neurol 2025;24(7):614–624. 10.1016/S1474-4422(25)00175-9 40541211

[84] Kiechle ES , Bailey SC , Hedlund LA , Viera AJ , Sheridan SL . Different measures, different outcomes? A systematic review of performance‐based versus self‐reported measures of health literacy and numeracy. J Gen Intern Med 2015;30(10):1538–1546. 10.1007/s11606-015-3288-4 PMC4579206 25917656

[85] Hussain N , Alt Murphy M , Lundgren‐Nilsson Å , Sunnerhagen KS . Relationship between self‐reported and objectively measured manual ability varies during the first year post‐stroke. Sci Rep 2020;10(1):5093. 10.1038/s41598-020-61834-1 PMC7083900 32198393

[86] Lynch DR , Goldsberry A , Rummey C , et al. Propensity matched comparison of omaveloxolone treatment to Friedreich ataxia natural history data. Ann Clin Transl Neurol 2024;11(1):4–16. 10.1002/acn3.51897 PMC10791025 37691319

[87] Klockgether T , Synofzik M , AGI working group on COAs and Registries , et al. Consensus recommendations for clinical outcome assessments and registry development in ataxias: ataxia global initiative (AGI) working group expert guidance. Cerebellum 2024;23(3):924–930. 10.1007/s12311-023-01547-z PMC11102398 37020147

[88] Indelicato E , Delatycki MB , Farmer J , França MC Jr , Perlman S , Rai M , Boesch S . A global perspective on research advances and future challenges in Friedreich ataxia. Nat Rev Neurol 2025;21(4):204–215. 10.1038/s41582-025-01065-y 40032987

[89] Buchholz M , Monier V , Ewenczyk C , et al. A patient‐reported outcome measure of communication difficulties in Friedreich ataxia: COMATAX. Cerebellum 2026;25(4):85. 10.1007/s12311-026-02020-3 PMC13197451 42174309

[90] Cook CE . Clinimetrics corner: the minimal clinically important change score (MCID): A necessary pretense. J Man Manip Ther 2008;16(4):82E–83E. 10.1179/jmt.2008.16.4.82E PMC2716157 19771185

[91] Wedding IM , Kroken M , Henriksen SP , et al. Friedreich ataxia in Norway‐an epidemiological, molecular and clinical study. Orphanet J Rare Dis 2015;10:108. 10.1186/s13023-015-0328-4 PMC4559212 26338206

[92] Silva CB , Yasuda CL , D'Abreu A , Cendes F , Lopes‐Cendes I , França MC . Neuroanatomical correlates of depression in Friedreich's ataxia: a voxel‐based morphometry study. Cerebellum 2013;12(3):429–436. 10.1007/s12311-012-0424-0 23090212

[93] Destrebecq V , Comet C , Deveylder F , Alaerts N , Naeije G . Determinant of the cerebellar cognitive affective syndrome in Friedreich's ataxia. J Neurol 2023;270(6):2969–2974. 10.1007/s00415-023-11623-3 36790547

[94] Dogan I , Tinnemann E , Romanzetti S , et al. Cognition in Friedreich's ataxia: a behavioral and multimodal imaging study. Ann Clin Transl Neurol 2016;3(8):572–587. 10.1002/acn3.315 PMC4999591 27606341

[95] Sayah S , Rotgé JY , Francisque H , et al. Personality and neuropsychological profiles in Friedreich ataxia. Cerebellum 2018;17(2):204–212. 10.1007/s12311-017-0890-5 29086357

[96] White VB , Leib JR , Farmer JM , Biesecker BB . Exploration of transitional life events in individuals with Friedreich ataxia: implications for genetic counseling. Behav Brain Funct 2010;6(65):1–12. 10.1186/1744-9081-6-65 PMC2987979 20979606

[97] Ejaz R , Chen S , Isaacs CJ , et al. Impact of mobility device use on quality of life in children with Friedreich ataxia. J Child Neurol 2018;33(6):397–404. 10.1177/0883073818764941 29607705

[98] Karatas D . The emotional and physical problems of people with Friedreich's ataxia and their perception about discrimination. Kahramanmaras Sutcu Imam Univ Soc Sci J 2024;21(2):822–835.

[99] Giunti P , Greenfield J , Stevenson AJ , et al. Impact of Friedreich's ataxia on health‐care resource utilization in the United Kingdom and Germany. Orphanet J Rare Dis 2013;28(8):38. 10.1186/1750-1172-8-38 PMC3599305 23448170

[100] Polek B , Roach MJ , Andrews WT , Ehling M , Salek S . Burden of Friedreich's ataxia to the patients and healthcare Systems in the United States and Canada. Front Pharmacol 2013;4:66. 10.3389/fphar.2013.00066 PMC3660667 23734128

[101] Winser SJ , Chan AYY , Whitney SL , Chen CH , Pang MYC . Effectiveness and cost of integrated cognitive and balance training for balance and falls in cerebellar ataxia: a blinded two‐arm parallel group RCT. Front Neurol 2023;14:1267099. 10.3389/fneur.2023.1267099 PMC10834731 38313407

[102] Kelly MJ , Bogdanova‐Mihaylova P , Skeens J , Moran S , Farrelly S , Walsh RA , Murphy SM . The cost of living with inherited ataxia in Ireland. Cerebellum 2022;21(2):280–296. 10.1007/s12311-021-01271-6 PMC8993771 34228323

[103] Buchholz M , Weber N , Borel S , et al. Patient‐reported, health economic and psychosocial outcomes in patients with Friedreich ataxia (PROFA): protocol of an observational study using momentary data assessments via mobile health app. BMJ Open 2023;13(8):e075736. 10.1136/bmjopen-2023-075736 PMC10394552 37527887

[104] Grobe‐Einsler M , Borel S , Buchholz M , et al. Patient‐reported, psychosocial and health economic outcomes in mild to moderate Friedreich's ataxia: baseline results of the PROFA study. Lancet Reg Health Eur 2026;61:101552. 10.1016/j.lanepe.2025.101552 PMC12756708 41488489

[105] Thomas‐Black G , Dumitrascu A , Garcia‐Moreno H , et al. The attitude of patients with progressive ataxias towards clinical trials. Orphanet J Rare Dis 2022;17(1):1. 10.1186/s13023-021-02091-x PMC8729009 34983593

[106] University of Florida . Respiratory Training in Friedreich's Ataxia‐Clinical Study Protocol; 2025. https://clinicaltrials.gov/study/NCT06539598.

[107] IRCCS Eugenio Medea . Efficacy of Stabilometric Platform to Improve Standing Balance in Patients With Friedreich's Ataxia‐Clinical Study Protocol; 2025. https://clinicaltrials.gov/study/NCT06692296.

[108] The University of Melbourne . Speech rehabilitation for dysarthria resulting from spinocerebellar ataxia or Friedreich ataxia; 2025. https://trialsearch.who.int/Trial2.aspx?TrialID=ACTRN12616001582448.

[109] The University of Melbourne . SpeechATAX: A 2‐arm, examiner‐blinded, wait‐list control group randomized controlled trial of intensive home based biofeedback driven speech rehabilitation in hereditary ataxia; 2025. https://trialsearch.who.int/Trial2.aspx?TrialID=ACTRN12618000519257.

[110] Children's Hospital of Philadelphia . NAD+ and Exercise in FA (ExRx in FA); 2025. https://clinicaltrials.gov/study/NCT04192136.

[111] Friedreich's Ataxia Research Alliance . Friedreich's Ataxia drug development pipeline. Downingtown, PA: Friedreich's Ataxia Research Alliance; 2025.

[112] Freidreich's Ataxia Research Aliance . Improve Mitochondrial Function & Reduce Oxidative Stress, Vatiquinone (PTC‐743); 2025. Accessed August28, 2025. https://www.curefa.org/drug-development/vatiquinone-ptc-743/.

[113] The National Institute of Neurological Disorders and Stroke . Start‐up resource–NINDS Friedreich's ataxia CDE recommendations; 2023.

[114] Lynch DR , Pandolfo M , Schulz JB , et al. Common data elements for clinical research in Friedreich's ataxia. Mov Disord 2013;28(2):190–195. 10.1002/mds.25201 PMC3581713 23239403

[115] Brandsema JF , Stephens D , Hartley J , Yoon G . Intermediate‐dose idebenone and quality of life in Friedreich ataxia. Pediatr Neurol 2010;42(5):338–342. 10.1016/j.pediatrneurol.2010.01.004 20399388

[116] Tekin HG , Levent E . Neurological recovery with interferon‐gamma treatment in Friedreich's ataxia. J Coll Physicians Surg Pak 2022;32(5):671–673. 10.29271/jcpsp.2022.05.671 35546709

[117] Egger K , Clemm von Hohenberg C , Schocke MF , et al. White matter changes in patients with friedreich ataxia after treatment with erythropoietin. J Neuroimaging 2014;24(5):504–508. 10.1111/jon.12050 PMC3950637 24015771

[118] Costantini A , Giorgi R , D'Agostino S , Pala MI . High‐dose thiamine improves the symptoms of Friedreich's ataxia. BMJ Case Rep 2013;2013:bcr2013009424. 10.1136/bcr-2013-009424 PMC3669970 23704441

[119] Sanz‐Gallego I , Torres‐Aleman I , Arpa J . IGF‐1 in Friedreich's ataxia – proof‐of‐concept trial. Cerebellum Ataxias 2014;1(1):10. 10.1186/2053-8871-1-10 PMC4552279 26331034

[120] Elincx‐Benizri S , Glik A , Merkel D , et al. Clinical experience with deferiprone treatment for Friedreich ataxia. J Child Neurol 2016;31(8):1036–1040. 10.1177/0883073816636087 27029487

[121] Qureshi MY , Patterson MC , Clark V , et al. Safety and efficacy of (+)‐epicatechin in subjects with Friedreich's ataxia: A phase II, open‐label, prospective study. J Inherit Metab Dis 2021;44(2):502–514. 10.1002/jimd.12285 32677106

[122] Igoillo‐Esteve M , Oliveira AF , Cosentino C , et al. Exenatide induces frataxin expression and improves mitochondrial function in Friedreich ataxia. JCI Insight 2020;5(2):1–19. 10.1172/jci.insight.134221 PMC7098728 31877117

[123] Berntsson SG , Holtz A , Melberg A . Does intrathecal baclofen have a place in the treatment of painful spasms in friedreich ataxia? Case rep neurol. Case Rep Neurol 2013;5(3):201–203. 10.1159/000356823 PMC3861848 24348400

[124] Kalyvas AV , Drosos E , Korfias S , Gatzonis S , Themistocleous M , Sakas DE . Intrathecal baclofen therapy for painful muscle spasms in a patient with Friedreich's ataxia. Stereotact Funct Neurosurg 2018;96(2):127–130. 10.1159/000489220 29886479

[125] Peter S , Manousaridis K , Boesch S , Mennel S . Memantine for optic nerve atrophy in Friedreich's ataxia. Ophthalmologe 2016;113(8):704–707. 10.1007/s00347-015-0191-7 26666888

[126] Seco, J. , Fernández, IG ., Atutxa, AF ., Torres‐Unda, J. , Verdejo, IC. , & Pérez, VR . Improvements in quality of life in individuals with Friedreich's ataxia after participation in a 5‐year program of physical activity: an observational study pre‐post test design, and two years follow‐up. Int J Neurorehabil 2014;01:3.

[127] Battal G , Pinsault N , Hanna‐Boutros B . Intensive multimodal treatment for A young adult with Friedreich ataxia: A case report. Int J Physiother Res 2023;11(3):4508–4516.

[128] Samardzic V . Efficiency of a 3‐week multicomponent rehabilitation on improving the function in a patient with Friedreich's ataxia: A case report. Vojnosanit Pregl 2023;80(2):182–187.

[129] Teixeira FAC , Sales BCM , Ferreira EF , Costa EA , Damasceno LN , Pereira ET . Friedreich's ataxia: dance and somatic education a case report. Motriz Rev Educ Fis 2018;24(3):e0047‐18.

[130] Valero MJ , Muñoz‐Blanco JL , Sanchez AG , et al. Should advanced Friedreich's ataxia Be a contraindication for heart transplantation? A case report of a successful procedure in a 58‐year‐old patient. J Cardiovasc Dev Dis 2022;9(3):80. 10.3390/jcdd9030080 PMC8952784 35323628

[131] Anderson HN , Burkhart HM , Johnson JN . Septal myectomy for hypertrophic obstructive cardiomyopathy in Friedreich's ataxia. Cardiol Young 2016;26(1):175–178. 10.1017/S104795111500013X 25687499

[132] Hayiroglu MI , Kalenderoglu K , Gurkan K . Tachycardiomyopathy treated with ablation by using 3D mapping system in a patient with Friedreich ataxia. Pacing Clin Electrophysiol 2025;48(2):227–229. 10.1111/pace.15125 39717928

[133] Portaro S , Russo M , Bramanti A , et al. The role of robotic gait training and tDCS in Friedrich ataxia rehabilitation: A case report. Medicine (Baltimore) 2019;98(8):e14447. 10.1097/MD.0000000000014447 PMC6407999 30813143

[134] de Freitas Zanona A , Santos AC , Gomes Goncalves AL , et al. Intervenção Terapêutica Ocupacional junto à duas jovens com Ataxia de Friedreich:um relato de caso. Rev Ocup Hum 2015;15(2):18–30.

[135] McGarrell NT , Green ME , McCully KK . Muscle endurance training in a person with Friedreich's ataxia. Muscles 2025;4(1):1. 10.3390/muscles4010001 PMC12121318 40757576

[136] Batista DDJ , Conceição ASD . Improving oral communication in a jornalism student with Friedreich ataxia: A case report. Rev CEFAC 2025;27(3):e10524.

[137] Joyce MR , Nadkarni PA , Kronemer SI , et al. Quality of Life Changes Following the Onset of Cerebellar Ataxia: Symptoms and Concerns Self‐reported by Ataxia Patients and Informants. Cerebellum 2022;21(4):592–605. 10.1007/s12311-022-01393-5 35334077

