# Supplementary material for: Key Interventions in Friedreich's Ataxia and Their Impact on Patient Outcomes: A Systematic Review
Source: Mov Disord. 2026 Jul 6;41(10):2564–91. doi: 10.1002/mds.70423 (PMC13686107; doi:10.1002/mds.70423)
Supplement: Supplementary file 1 [file MDS-41-2564-s001.pdf]

Supplementary materials to the publication:

Key Interventions in Friedreich's Ataxia and their Impact on Patient Outcomes: A Systematic Review

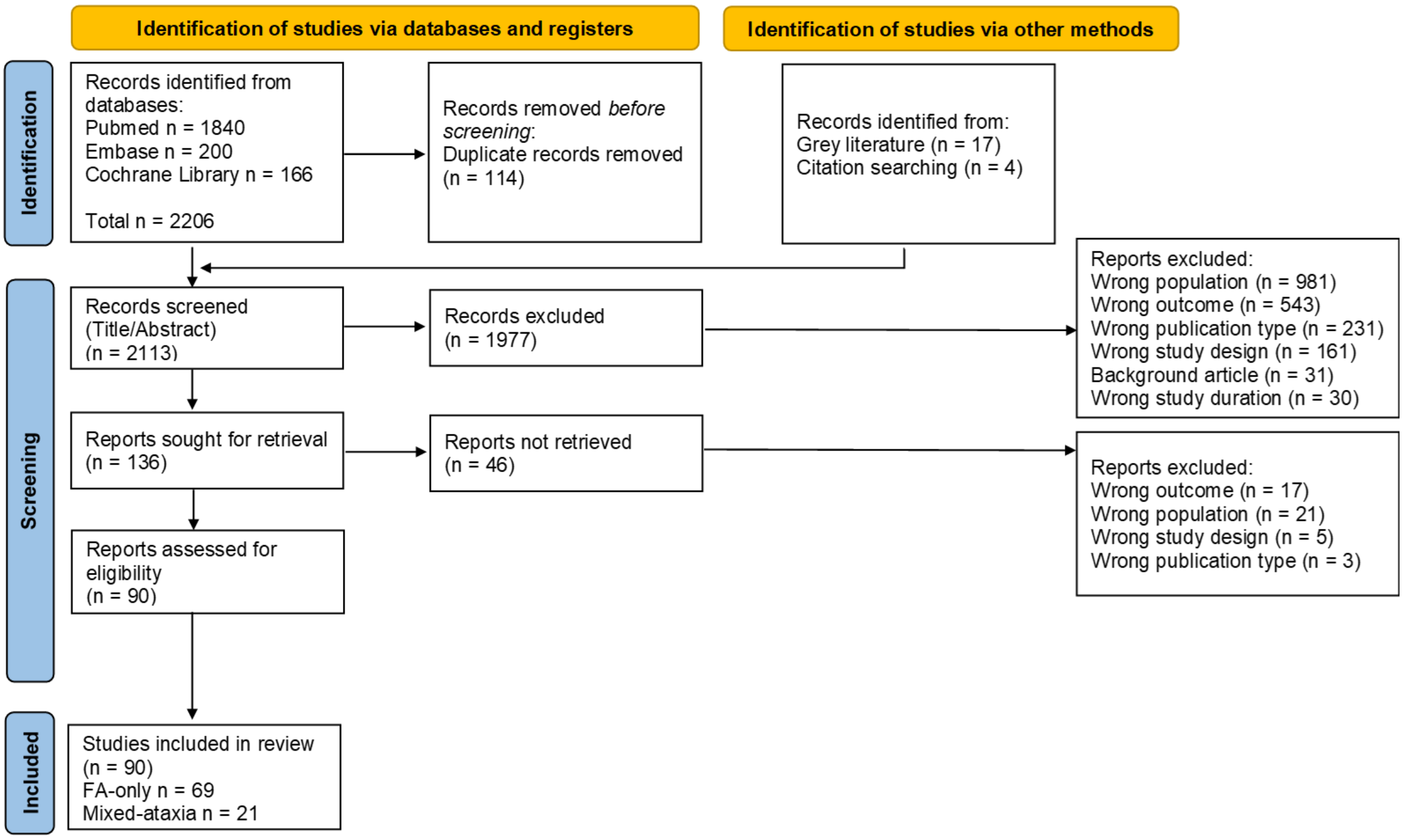

Figure S1: *PRISMA chart: the process of studies selection [1].*

Table S1: *Inclusion and exclusion criteria of studies in the presented review according to PICOS methodology [2].*

		Inclusion criteria	Exclusion criteria
**P**	**Population**	- Patients with Friedreich's Ataxia, at all ages, all onsets and all genotypes,<br>- Studies that include patients with FA in studies together with other ataxias (hereditary ataxia/spinocerebellar ataxia,ect.)	Animal studies<br>Cell studies<br>Studies that include only patients with ataxias other than Friedreich's ataxia
**I**	**Intervention**	Interventions in FA for patients with FA and their caregivers:<br>- pharmacological interventions (symptomatic and disease-modifying), e.g. omaveloxolone, riluzole, gene therapies, idebenone, coenzyme Q10, vitamins,<br>- non-pharmacological interventions; e.g. physiotherapy, physical exercise, neuro-physiotherapy, rehabilitation, exergames/video games, virtual reality, visually guided stepping, treadmill, weightlifting, electric stimulation, nutrition, speech and language therapy (SLT), balance training, strength training, respiratory rehabilitation, brain stimulation, tai chi, yoga, swimming, hydrotherapy, fitness training, lycra garments, axial weighting, biofeedback, robotics, surgery, occupational therapy (OT), psychotherapy, support groups, meetings, workshops, work including potential interventions for caregivers e.g. psychological help, workshops and economic interventions.	*In vitro*, *in silico* calculations/simulations<br>Cell studies<br>Animal studies
**C**	**Control/ Comparison**	- Placebo<br>- Usual care<br>- No intervention<br>- Pre -post-intervention results	Studies without a comparison
**O**	**Outcome**	Outcomes of pharmacological and non-pharmacological interventions were divided in four groups:<br><br>1) Clinical outcomes – objective, clinical assessment, including use of laboratory and imaging outcomes e.g.<br>- Scale for the Assessment and Rating of Ataxia (SARA)<br>- International Cooperative Ataxia Rating Scale (ICARS)<br>- Friedreich's Ataxia Rating Scale (mFARS)<br>- Inventory of Non-ataxia Signs (INAS)<br>- Functional Independence Measure (FIM)<br>- Activities of Daily Living (ADL)<br>- Safety of Pharmacotherapy (Adverse Effects)	Feasibility, validity studies of outcome measures, assessment of diagnostic tools

		2) Patient-reported outcomes and<br>3) Caregiver outcomes - subjective well-being of individuals with FA and their caregivers, perception of living with FA e.g.<br>- Friedreich's Ataxia Impact Scale (FAIS)<br>- Health-Related Quality of Life HRQoL:<br>o EuroQol five Dimensions Questionnaire (EQ-5D/EQ-5D-(Y)-5L)<br>o Short Form-36 Health Survey (SF-36)<br>o Pediatric Quality of Life (PedsQL)<br>- Patient-Reported Outcomes Measures in Ataxia (PROM-Ataxia)<br>- Warwick-Edinburgh Mental Well-Being Scale (WEMWBS)<br>- Voice Handicap Index (VHI)<br>- Speech, Spatial and Qualities of Hearing Scale (SSQ12)<br>- Friedreich's Ataxia-Health Index (FA-HI)<br><br>4) Economic outcomes. e.g. cost and resource use, formal and informal care, medical services, Health Utilisation Questionnaire (HUQ)<br><br>Additional outcomes:<br>Other outcomes, whether subjective or objective, that relate to patients with FA or their caregivers will also be considered	
**S**	**Study design**	Randomised Controlled Trials (RCTs)<br>Quasi-experimental studies<br>Controlled interventional studies, Observational studies (prospective and retrospective) that include the intervention and assessment, cohort studies, quasi-randomised studies, case reports,	Systematic reviews, scoping reviews, qualitative studies, study protocols, observational studies without any intervention, longitudinal studies

Table S2: *Final search strategy applied in the presented review.*

Search strategy for the review: Key Interventions in Friedreich's Ataxia and their Impact on Patient Outcomes: A Systematic Review

The search strategy was developed based on the two dimensions i) Friedreich ataxia and ii) Intervention. The keywords used in the first dimension included Friedreich ataxia (MeSH), cerebellar ataxia, hereditary ataxia, and progressive ataxia. The search did not only include patients with FA due to the rare nature of the disease and the fact that several patients with FA are often included in studies together with patients who suffer from other (rare) ataxias.

Keywords included in the second dimension focused on a variety of interventions, including i.a. Intervention, rehabilitation [MeSH], treatment [MeSH], training, exercise, and therapy, and encompassed pharmacological and non-pharmacological aspects.

The search excluded reviews and systematic reviews from the process to limit the options to manual screening. Potential eligible publications included in previous reviews but not identified by the search strategy were manually screened. The searching strategy used in the certain databases was the following:

1) PubMed

("Friedreich ataxia"[MeSH Terms] OR "friedreich*"[Title/Abstract] OR "Cerebellar ataxia"[Title/Abstract] OR "neurodegenerative ataxia"[Title/Abstract] OR "progressive ataxia"[Title/Abstract] OR "hereditary ataxia"[Title/Abstract])
AND
("Rehabilitation"[MeSH Terms] OR "Rehabilitation"[Title/Abstract] OR "intervention*"[Title/Abstract] OR "Exercise"[MeSH Terms] OR "Exercise Therapy"[MeSH Terms] OR "Exercise"[Title/Abstract] OR "management"[Title/Abstract] OR "training"[Title/Abstract] OR "drug therapy"[MeSH Terms] OR "pharmacotherapy"[Title/Abstract] OR "medication"[Title/Abstract] OR "drug"[Title/Abstract] OR "gene therapy"[Title/Abstract] OR "physiotherapy"[Title/Abstract] OR "physical therapy modalities"[MeSH Terms] OR "treatment"[Title/Abstract] OR "physical activity"[Title/Abstract] OR "surgical procedures, operative"[MeSH Terms] OR "occupational therapy"[MeSH Terms] OR "speech therapy"[MeSH Terms] OR "language therapy"[MeSH Terms] OR "voice"[Title/Abstract] OR "speech"[Title/Abstract] OR "exergame"[Title/Abstract] OR "game"[Title/Abstract] OR "stimulation"[Title/Abstract] OR "use"[Title/Abstract] OR "app"[Title/Abstract] OR "scoliosis"[Title/Abstract] OR "support"[Title/Abstract])
NOT
("review"[Title/Abstract] OR "systematic review"[Title/Abstract])
NOT
("mice"[Title/Abstract] OR "mouse"[Title/Abstract] OR "cell"[Title/Abstract])
AND
(2010:**2025[pdat])**

Table S3: *Characteristics of included studies with pharmacological interventions for mixed ataxia patients, n = 4 studies.*

Author, year, Country	Study design	Sample size (N)<br>Age in years (Mean (+/-SD)<br>FA characteristics (AaO, DD, DoS, DS, AaD, YsD)	Medication<br>Dosis, route of administration	Duration of treatment / follow-up	Outcome type	Assessed feature/symptom	Outcome measure, result:<br>(+) improvement<br>(-) deterioration regardless of the treatment<br>0 no effect<br>**Core outcome measures**	Comparator, reported result
**Disease-modifying treatment:**								
**Ristori et al. 2010 [3]<br>Italy**	RCT	8 FA/40<br>#46.53 ± 15.12<br>DD: #9.90 ± 8.34 years	Riluzole, orally<br>100 mg daily (2x 50 mg)	8 weeks treatment	CL	Ataxia severity	ICARS total (+)*<br>(subscores static function*, kinetic function*, dysarthria*, ocular movement ns)	Placebo
**Romano et al. 2015 [4]<br>Italy**	RCT	20 FA/60<br>#44.65 ± 11.71<br>AaO: #32.7 ± 13.9	Riluzole, orally<br>100 mg daily (2x 50 mg)	12 months	CL	Ataxia severity	**SARA (+)***	Placebo
					PROM (QoL)	Quality of life for 15/15 treatment/placebo	SF-36 physical and mental score 0 ns<br>SF-36 social role functioning (+)*	
					PROM	Depression	BDI 0 ns	
**Blankenberg et al. 2012 [5]<br>United States**	Quasi-experimental study	2 FA/22<br>(17, 33)<br>> 800 GAA repetitions	Vitiquinone,<br>1 month gradual dose escalation up to 100 mg, 3x a day, orally or via gastrostomy or jejunostomy tube feeding for the following 3 months	3 months treatment	CL	Disease severity	NPMDS total (+)<br>(only in subscore quality of life -3 points)<br>Loss of all gains and return to baseline, acute cardiac failure after ~2 years of treatment	Pre- and post-intervention<br>W17
						Physical function	strength, exercise tolerance, speech fluency, social interaction (+)<br>partial regain of eyesight after 7 months	
					CL	Disease severity	NMDA total (+)<br>(the greatest improvement in subscore influence on daily living - 8 points)	W33
						Physical function	visual acuity, vocalization, strength (+)	
					PROM (QoL)	Quality of life	SF-12v2 (+) +12.9 points	
**Symptomatic treatment:**								
**Berntsson et al. 2019 [6]**		1 FA/5	Baclofen<br>Intrathecal delivery pump,	Mean treatment	PROM	Fatigue	FSS (+) 5/5	
						Pain	BPI (+) 4/4	

**Sweden**	Quasi-experimental study	(52 yo) mean all: 53.6 ± 11.3<br>DD: 26.8 years	75-400 µg daily (For FA 110 µg)	time 1.9 ± 1.0 years (1-4)		Life satisfaction	LiSAT-9 (+) 4/4<br>(especially in ability to manage self-care)	Pre- and one year post treatment
					CL	Muscle spasticity	MAS (+) 5/5	
						Spasms frequency	PSFS (+) 5/5	

Info:
*significant effect
#mean (age/at onset, disease duration) calculated from the available data in the publication

Abbreviations:
Disease description: AaO age at onset, DD disease duration, DoS Duration of Symptoms, DS Disease stage, AaD age at diagnosis, YsD Years since diagnosis
Type of the measure: CL clinical, PROM patient reported, C caregiver, L laboratory, IMG imaging, QoL Quality of life, S subjective
Outcome measures: BDI Beck Depression Inventory, BPI The Brief Pain Inventory, FSS The Fatigue Severity Scale, ICARS International Cooperative Ataxia Rating Scale, LiSAT-9 Life Satisfaction Questionnaire, 9-item version, MAS The Modified Ashworth Scale, NMDA Newcastle Mitochondrial Disease Adult Scale, NPMDS Newcastle Paediatric Mitochondrial Disease Scale, ns not significant, PSFS Patient-Specific Functional Scale, SARA Scale for the Assessment and Rating of Ataxia, SF-12v2 shorter version of the SF-36, SF-36 Short Form Health Survey with 36 questions,

Tabelle S4: *Characteristics of included studies with non-pharmacological interventions in mixed ataxia patients, n = 17 studies.*

Author, year, Country	Study design	Sample size (N)<br>Age in years (Mean (+/- SD)<br>FA characteristics<br>(AaO, DD, DS, AaD, YsD)	Intervention	Duration of intervention/ follow-up	Outcome type	Assessed feature/symptom	Outcome measure, result:<br>(+) improvement<br>(-) deterioration regardless of the treatment<br>0 no effect<br>**Core outcome measures**	Comparator, reported result
**Transcranial Direct Current Stimulation**								
**Benussi et al. 2015 [7] Italy**	RCT	1 FA/19<br>53.8 ± 18.4<br>n.i.	Cerebellar tDCS	2 sessions separated at least 1 week	CL	Ataxia severity	**SARA** (+)*	Control - sham stimulation
							ICARS (+)*	
						Manual dexterity and fine motor coordination	**9-HPT** (+) *	
						Walking speed	8MW (+) *	
**Benussi et al. 2017 [8] Italy**	RCT	1 FA/20<br>#53.04 ± 17.84<br>AaO: #39.16 ± 20.99	Cerebellar tDCS	2 weeks, follow-up: 1 month, 3 months	CL	Ataxia severity	**SARA** (+)*/(+)*/(+)*	Pre- and post-treatment: 2 weeks/1 month/3 months,<br>No significant changes in the sham control group
							ICARS (+)*/(+)*/(+)*	
						Manual dexterity and fine motor coordination	**9-HPT** dominant hand (+)*/(+)*/0 ns<br>**9-HPT** non-dominant hand (+)*/(+)*/0 ns	
						Walking speed	8MW 0 ns/0 ns/ 0 ns	
					PROM (QoL)	Quality of life	SAQOL-39 (+)*/0 ns/ 0 ns	
					L	Cerebellar modulation of motor cortex	CBI 0 ns/(+)*/(+)*	
**Benussi, et al. 2018 [9] Italy**	RCT	1 FA/20<br>54.6 ± 14.5<br>AaO: 41.7 ± 19.5	Cerebello-spinal tDCS	2x 2 weeks of stimulation (5 days a week), either treatment or sham. Follow-up after 2 weeks, 1 month and 3 months following 3 months wash out phase.	CL	Ataxia severity	**SARA** (+)*/(+)*/(+)*	Pre- and post-treatment: 2 weeks/1 month/3 months,<br>No significant changes in the sham control group
							ICARS (+)*/(+)*/(+)*	
						Manual dexterity and fine motor coordination	**9-HPT** dominant hand (+)*/(+)*/(+)*<br>**9-HPT** non-dominant hand (+)*/(+)*/(+)*	
						Walking speed	8MW (+)* /(+)*/ (+)*	
					PROM (QoL)	Quality of life	SF-36 (+)*/(+)*/(+)*	
					L	Cerebellar modulation of motor cortex	CBI (+)*/(+)*/(+)*	
**Benussi et al. 2021 [10]**	RCT	7 FA/61<br>56.9 ± 13.9	Cerebello-spinal tDCS	12 weeks sham-controlled phase:	CL	Ataxia severity	**SARA** (+)*/ (+)*	Randomised double-blind
							ICARS (+)*/(+)*	

**Italy**		AaO: 43.6 ± 18.4		2 weeks of stimulation (5 days a week), either treatment or sham. Follow-up after 2 and 12 weeks, open label phase: 2 weeks of stimulation (5 days a week), follow-up: 14 weeks 24 weeks 36 weeks 52 weeks		Cognitive and emotional changes	CCASS (+)*/(+)*	phase, sham-controlled, 2/12 weeks
					PROM (QoL)	Quality of life	SF-36 (+)*/(+)*	
					L	Cerebellar modulation of motor cortex	CBI (+)*/(+)*	
					CL	Ataxia severity	**SARA** (+)*/(+)*/ (+)*/(+)*	Open-label phase, real/real tDCS vs sham/real tDCS, 12/24/36/52 weeks
							ICARS (+)*/(+)*/ (+)*/(+)*	
						Cognitive and emotional changes	CCASS 0 ns/0 ns/ 0 ns/(+)*	
					PROM (QoL)	Quality of life	SF-36 (+)*/(+)*/ (+)*/(+)*	
					L	Cerebellar modulation of motor cortex	CBI 0 ns/0 ns/ 0 ns/(+)*	
**Brito et al. 2024 Brasil [11]**	RCT	2 FA/22 #49.65 ± 16.02 DD for FA patients 6.5 All #6.55 ± 4.18	Cerebellar tDCS + gait training (both simultaneously)	After last interventoion (5 days)	CL	Functional mobility	TUG (+)*/(+)*	Pre- and post-intervention within groups, real/sham
						Balance	MiniBESt 0 ns/ 0 ns	
						Ataxia severity	**SARA** total (+)*/0 ns	
					PROM	Perception of change	PGI-C 0 ns	Between groups
**Speech therapy**								
**Lowit et al. 2020 [12] UK**	Quasi-experimental study	18 FA/21 44.5 ± 17.3 YsD: 18.7 ± 8.9	Speech therapy with Lee Silverman Voice Treatment (video call)	2 years	CL	Speech assessment	Prolonged vowel duration (+)*<br>Voice quality measures (+)*<br>Intelligibility 0 ns<br>Naturalness 0 ns	Pre- and post-treatment
					PROM	Fatigue during treatment	FIS 0 ns	
						Self-perception and impact of dysarthria	VHI 0 ns<br>CPIB 0 ns	
					PROM (S)	Self-perceived speech assessment	Louder speech 14/21<br>Clearer speech (+) 13/21<br>Longer phrases/speaking time (+) 13/21<br>Increased confidence/reduced anxiety (+) 10/21	
**Lowit et al. 2023 [13] Scottland England**	Quasi-experimental study	1 FA/9 57, mean all: #59.67 ± 7.42	Speech therapy with ClearSpeechTogether (video call)	16 weeks	PROM	Fatigue	FIS 0 ns	Pre- and post-treatment
					CL	Speech assessment	Maximum phonation time 0 ns<br>Vocal strain (+)*<br>Reading intelligibility (+)*	

**Netherlands**		YSD: 10, mean all: 6.06 ± 5.27			PROM	Self-perception and impact of dysarthria	CPIB (+)*	
						Self-perceived speech assessment	Stronger, louder, more stable voice<br>Lower effort to speak<br>Impact of group support (+)	
**Knuijt et al. 2024 [14] Netherlands**	Quasi-experimental study	4 FA/21<br>Mean age<br>#56.43 ± 19.03<br>(27, 29, 38, 21)<br>DS: 3, 1, 1, 1	Voice Trainer App<br>(The principles of the Pitch Limiting Voice Treatment)	3 weeks	CL	Speech assessment	Intelligibility 0 ns<br>Naturalness (DME) 0 ns<br>Speaking rate (+)*<br>Mean pause duration (+)*	Pre- and direct post-treatment/ 3 months after the treatment no significant changes
					PROM	Self-perceived speech assessment	Dysarthria questionnaire RDA 0 ns<br>CPIB-10 0 ns	
					PROM C	Satisfaction with speech quality	Patient- / caregiver reported 0 ns/ 0 ns	
**Video games**								
**Romano et al. 2022 [15] Italy**	RCT	6 FA/18<br>11.6 ± 3.5<br>n.i.	Rehabilitation through IMU-Based Exergame Motor activity program Niurion® Exergame	12 weeks	CL	Manual dexterity and fine motor coordination	**9-HPT** dominant hand (+)*<br>**9-HPT** non-dominant hand (+) ns	Control group
						Ataxia severity	**SARA** 0 ns	
						Walking speed, mobility	**T25FW** 0 ns	
**Ilg et al. 2012 [16] Germany**	Quasi-experimental study	4 FA/10<br>15.4 ± 3.5<br>AaO: 7, 6, 14, 12	Video game-based (Microsoft Xbox Kinect, games: “Table Tennis,” “Light Race,” and “20000 Leaks) coordinative training	8 weeks	CL	Ataxia severity	SARA (+) *<br>(especially in the posture score)	Pre- and post-treatment
						Gait stability	DGI (+) *	
						Gait analysis	Gait (+) ns<br>lateral sway (+)*, step length variability (+)* and in goal-directed leg placement (+)*	
					PROM	Confidence in maintaining balance during activities	ABC (+) ns	
						Pleasure and motivation for training	Pleasure and motivation for training (+)	
**Schatton et al. 2017 [17] Germany**	Quasi-experimental study	5 FA/10<br>#16.0 ± 7.4 (for all)<br>(13, 11, 29, 24, 19)<br>Advanced stadium FA	Exergame training, body-controlled videogames	12 weeks	CL	Ataxia severity	**SARA** (+)*	Pre- and post-intervention
					PROM	Progress in the individual goal	GAS (+)*	
					CL	Postural stability	Body sway with eyes open/eyes closed 0 ns/ (+)*	
**Rehabilitation**								
**Yigit et al. 2022 [18]**	RCT	5 FA/20<br>#13.45 ± 3.28	Routine rehabilitation program + the Bobath	3 days a week, 8 weeks	CL	Ataxia severity	ICARS total 0 ns	
						Trunk control	TIS total 0 ns	

**Turkey**		n.i.	Neurodevelopmental Treatment (NDT)			Manual dexterity and fine motor coordination	**9-HPT** (R,L) 0 ns	Control group - routine rehabilitation<br><br>Pre-and post intervention - significant improvement in both groups
					PROM	Upper limb disability	Q-DASH 0 ns	
					CL	Dynamic balance of the trunk	MFRT (AP,ML) (+)*	
**Cabanas-Valdes et al. 2024 [19] Spain**	RCT	6 FA/23<br>51.8 ± 11.1<br>n.i.	Rehabilitation for core stability<br>Standard care + CSE programm	5 weeks treatment and follow-up<br>10 weeks follow-up	CL	Ataxia severity	**SARA** total 0 ns/ 0 ns	Control - standard care<br>5 weeks/10 weeks
						Trunk control and stability	S-TIS 2.0 total 0 ns/ 0 ns	
						Confidence in maintaining balance during activities	ABC (+)*/ (+)*	
						Short-distance walking speed	4-MWT (+)*/ 0 ns	
						Lower limb motor function	30-s sit-to-stand test 0 ns / 0 ns	
					PROM (QoL)	Quality of life	EQ-5D-5L (+)* / (+)*<br>EQ-5D health status (+)*/(+)*	
**Ilg et al. 2010 [20] Germany**	Quasi-experimental study	3 FA/6 in a group with afarent ataxia<br>(51, 69, 64)<br>AaO: 31, 44, 44	Physiotherapy - coordinative training	4 weeks training and follow-up,<br>1 year home training follow-up	CL	Ataxia severity	**SARA** (+)* / **SARA** (-)*	Pre- and post intervention (After 4 weeks/after 1 year)
						Balance and risk of fails	BBS (+)* / BBS (-)*	
						Gait and dynamic balance	Gait and dynamic balance 0 ns	
					PROM	Progress in the individual goal	GAS (+)	
**Vibration**								
**Leonardi et al. 2017 [21] Italy**	Quasi-experimental study	3 FA/9<br>35.33 ± 16.3<br>FA: 15.67 ± 3.1<br>DD: total 6.22 ± 1.6<br>DD FA 6.33 ± 0.2	Focal mechanical vibrations (A wearable proprioceptive stabiliser (Equitasi device)) + physiotherapy	3 weeks device utilisation, 3 weeks after the discontinuation	CL	Ataxia severity	**SARA** (+)*	Pre- and post-treatment
						Manual dexterity and fine motor coordination	**9-HPT** dominant hand (+)*<br>**9-HPT** non-dominant 0 ns	
						Speech motor control	PATA test (+)*	
						Mobility, endurance	6-MWT (+)*	
						Gait	Gait parameters (+)*<br>(except speed, length step right/cycle, and length step left/cycle)	
						All (-) after discontinuation (except length of cycle)		
**Schirinzi, T., et al 2018 [22]**	Quasi-experimental study	6 FA/10<br>13.10 ± 7.30<br>FA: 15.66 ± 8.40	Non-invasive focal mechanical vibrations (NIFMV)	4 weeks device use,	CL	Ataxia severity	**SARA** (+) (-2.13 points) (ns)	Pre- and post-treatment, for FA patients
						Manual dexterity and fine motor coordination	**9-HPT** dominant (+) (-6.78 s) (ns)<br>**9-HPT** non-dominant (+) (-3.43 s) (ns)	

**Italy**		AaO: < 18 yo	+ physiotherapy	follow-up after 8 weeks (4 weeks wash-out after 4 weeks of use)		Mobility, endurance	6-MWT (+) (+6.04 m) (ns)	
						Gait	Gait parameters 0 ns	
**Multi-disciplinary therapy/combined therapy (rehabilitation + other)**								
**Jorge-Rodríguez 2013 [23] Cuba**	Quasi-experimental study	4 FA/20<br>#27.50 ± 8.46<br>(19, 16, 30, 22)<br>n.i.	Neurorestorative treatment (incl. physical therapy, occupational therapy, speech therapy)	28 days<br>42 days<br>After treatment	CL	Ataxia severity	ICARS total (+)*/ (+)*<br>(subscales:<br>posture and gait disturbances, kinetic function (+)*/(+)*<br>Speech disorders 0 ns/ (+)*)	Pre- and post-intervention 28/42 days

Info:

*significant effect

#mean (age/at onset, disease duration) calculated from the available data in the publication

Abbreviations:

Disease description: AaO age at onset, DD disease duration, DS Disease stage, AaD age at diagnosis, YsD Years since diagnosis

Type of the measure: CL clinical, PROM patient reported, C caregiver, L laboratory, IMG imaging, QoL Quality of life, S subjective

Outcome measures: ABC Activities Specific Balance Confidence, CBI Cerebellar Brain Inhibition, CCASS Cerebellar Cognitive Affective Syndrome Scale, CPIB (-10) Communicative Participation Item Bank (-short form), DGI Dynamic Gait Index, EQ-5D-(5L) EuroQol 5-Dimension (5-Level) questionnaire, FIS Fatigue Impact Scale, GAS Goal Attainment Score, ICARS International Cooperative Ataxia Rating Scale, MFRT (AP,ML) Modified Functional Reach Test (Anterior-Posterior, Mediolateral), MiniBESt Short version of Balance Evaluation System Test, n.i. no information, ns not significant, PGI-C Patient Global Impresion of Change, Q-DASH Quick Disabilities of the Arm, Shoulder, and Hand questionnaire, RDA Radboud Dysarthria Assessment, SARA Scale for the Assessment and Rating of Ataxia, S-TIS Spanish-version of Trunk Impairment Scale 2.0, SAQOL-39 Stroke and Aphasia Quality of Life Scale – 39 items, SF-36 SF-36 Short Form Health Survey with 36 questions, TIS Trunk Impairment Scale, TUG Timed Up and go test, T25FW Timed 25-Foot Walk, VHI Voice Handicap Index, 4-MWT 4-meter walk test, 6MWT 6-Minute Walk, 8MWT 8-Meter Walking Time, 9-HPT 9-Hole Peg Test,

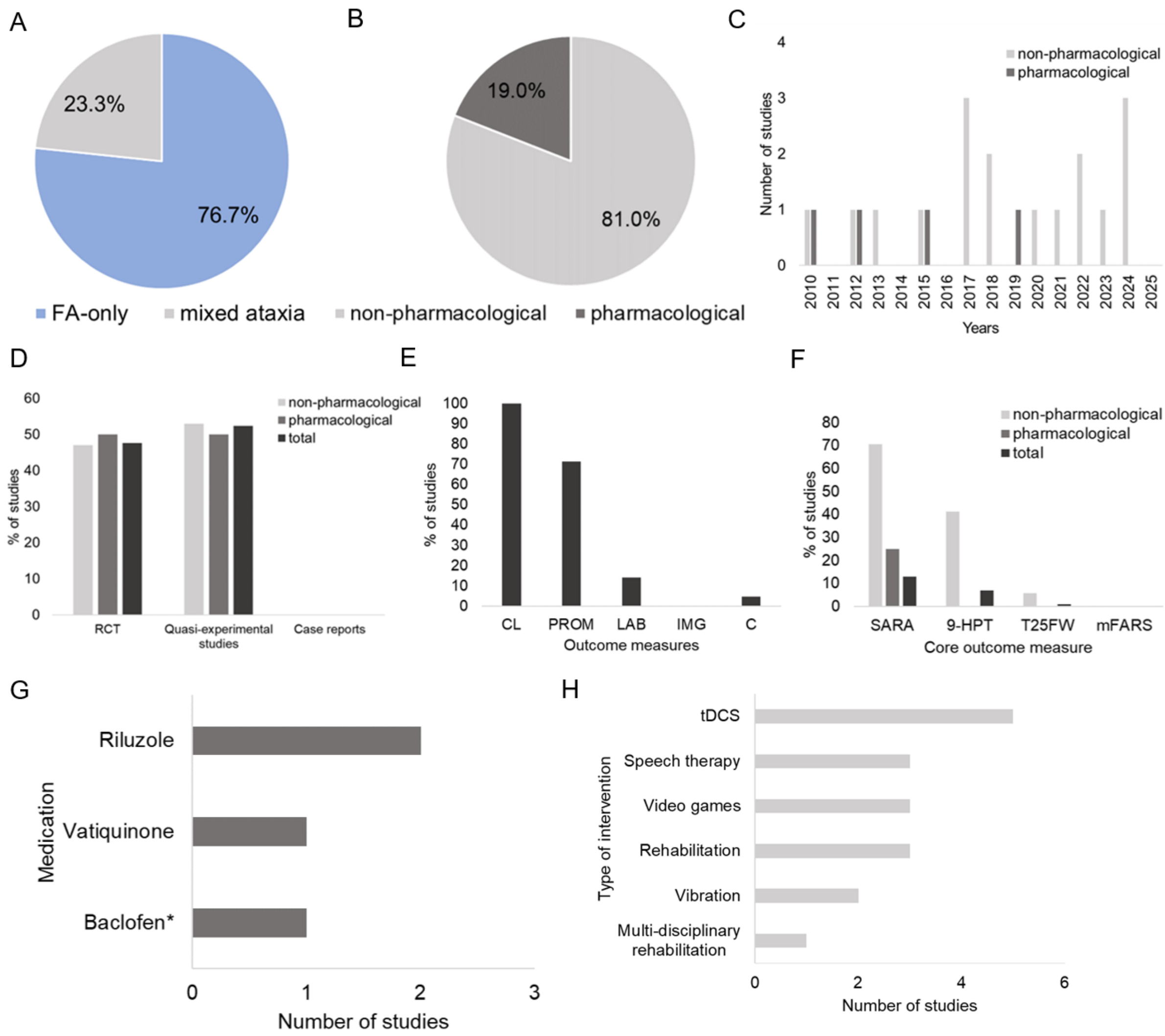

Figure S2: *Characteristics of included studies for mixed-ataxia patients. A) Study populations from all included publications, n = 69 studies for FA-only patients, n = 21 for mixed ataxia patients. B) Interventions types, n = 4 pharmacological interventions (P), n = 17 non-pharmacological interventions (NP); C: Timeline for pharmacological and non-pharmacological studies; D: Study designs of included studies regarding an intervention type and total number of studies; E: Outcome measures used in included studies: CL Clinical, L Laboratory, PROM Patient reported outcome measure (incl. Subjective and Quality of life), IMG Imaging; F: The prevalence of core outcome measures used in included studies: SARA, Scale for the Assessment and Rating of Ataxia; 9-HPT, 9-Hole Peg Test; T25FW, Timed 25-Foot Walk; mFARS, modified Friedreich Ataxia Rating Scale [24, 25]; G: Types of pharmacological interventions, n = 4: disease-modifying n = 3, *symptomatic n = 1; H: Types of non-pharmacological interventions n = 17.*

Table S5: *Risk of bias assessment for Randomized Controlled Trials in FA-only population, n = 22, Cochrane RoB-2 [26].*

Study ID	Experimental	Comparator	Outcome	Weight	D1	D2	D3	D4	D5	Overall
Lynch 2012	Linoleic acid	Placebo	FARS, T25FW, 9HPT, LCSLA, GICS, MFIS, ADLs	1	!	+	+	+	+	!
Pandolfo 2014	Deferiprone	Placebo	9HPT, T25FW, LCLA, ICARS, FARS, heart function, QoL, ADL	1	+	!	+	-	+	!
Sacca 2016	Epoetin alfa	placebo	VO2, frataxin, echo, SARA, 9-HPT, EQ-5D IADL,	1	+	+	+	+	+	+
Mariotti 2012	Erythropoietin	Placebo	SARA, 9-HPT, SF-36, frataxin	1	!	+	+	!	!	!
Boesch 2014	Erythropoietin	Placebo	frataxin, SARA, FARS, CGI-I/S,	1	!	+	+	-	!	-
Paparella 2024	Etravirine	no comparator	VO2, SARA, heart function, WHO-DAS V2, SF-36, frataxin	1	!	+	+	!	+	!
Drinkard 2010	Idebenone	placebo	VO2, ICARS, FARS, echo, workload	1	+	!	!	-	!	-
Lynch 2010	idebenone	placebo	ICARS, FARS, FACT-Z, ADL,	1	+	+	+	+	+	+
Lagedrost 2011	Idebenone	placebo	ICARS, cardiac function	1	+	+	+	+	+	+
Meier 2012	idebenone	open label	ICARS, FARS,	1	+	+	+	+	+	+
Cook 2019	idebenone	placebo	heart function, status and change questionnaire, MFIS, 9-HPT, AIDS, CGI-C, ICARS	1	+	+	+	+	+	+
Lynch 2019	Interferon gamma	placebo	mFARS, total FARS, T25FW, 9-HPT, LCSLC, frataxin, ADL, PGA, CGA, MFIS, PedsQL, SF-36	1	+	+	+	+	+	+
Pandolfo 2022	leriglitazone	placebo	change in spinal cord, CCFS, 9-HPT, SARA, EQ-5D-5L, CGI-I, PGI-I, ADL	1	!	+	!	-	+	-
Zesiewicz 2018	linoleic acid (RT001)	inactive comparator	FARS-Neuro, T25FW, CPET, VO2 peak	1	+	!	+	+	!	!
Lynch 2023	deuterated linoleic acid	placebo	peak workload, VO2 max, T1MW, FARS-Neuro, CGI, mFACIT, GST	1	!	+	+	+	+	!
Wang 2021	luvadaxistat	placebo	9-HPT, ADL, mFARS Neuro, T25FW, LCLA, CGI-I, PGI-I,	1	+	+	+	+	+	+
Lynch 2019	omaveloxolone	placebo	mFARS, peak workload, T25FW, SF-36, FSS, 9-HPT, LCLVA	1	!	+	+	+	-	!
Lynch 2021	omaveloxolone	placebo	mFARS, PGCI, CGIC, 9-HPT, T25FW, frequency of falls, peak workload, FA-ADL,	1	+	+	+	!	+	!
Zesiewicz 2018	vatiquinone	placebo	LCVA, FARS neuro, T25FW, 9-HPT, echo	1	!	!	+	+	+	!
Seco Calvo 2014	rehabilitation+pharmacotherapy	pharmacotherapy	ICARS, SF-36, FIM	1	!	+	+	+	+	!
Milne 2018	rehabilitation	cross-over design (delayed start)	FIM, FAIS, FARS, PGIC, GAS, BBS, modified tardieu scale, foot posture index, gaitrite, phone-fi	1	!	+	+	!	!	!
Naeije 2023	anodal cerebellar tDCS	sham stimulation, cross-over design	SARA, CCFS, CCAS-S, fMRI	1	!	!	+	!	!	!

+	Low risk
!	Some concerns
-	High risk

D1	Randomisation process
D2	Deviations from the intended interventions
D3	Missing outcome data
D4	Measurement of the outcome
D5	Selection of the reported result

Table S6*: Risk of bias assessment for quasi-experimental studies in FA-only population, n = 24, JBI Critical Appraisal Tool [27]. The answers have been standardized and presented in the color manner according to the literature [28]:* ***≥75% "Yes" answers = low bias - high quality; 50–74% = moderate bias; <50% = high bias.***

Study identification			Critical appraisal (yes 2, no 0, unclear 1, not applicable 9)									Bias assessment, min to include 60% (10/16)			
			1	2	3	4	5	6	7	8	9				
Author	Year	Intervention category (internal question) P/NP	Clarity of intervention and outcome	Baseline characteristics equivalence	Consistency of intervention	Study design strength	Measurement quality over time	Attrition bias	Measurement reliability	Instrument /tool validity	Validity of conclusions	Sum	low (18-15)	moderate (14 - 10)	high (<9)
Alemany-Perna	2024	P	2	2	2	1	2	2	2	2	2	17	x		
Qureshi	2021	P	2	2	2	0	2	1	2	2	2	15	x		
Sacca	2011	P	2	2	2	1	2	2	2	2	2	17	x		
Nachbauer	2011	P	2	2	2	0	2	2	2	2	2	16	x		
Egger	2014	P	2	2	2	0	2	2	2	2	2	16	x		
Santner	2014	P	2	2	1	2	2	2	2	2	2	17	x		
Igoillo-Esteve	2020	P	2	2	1	1	2	1	2	2	1	14		x	
Sanz-Gallego	2014	P	2	2	1	1	2	2	2	2	2	16	x		
Seyer	2015	P	2	2	2	1	2	2	2	2	2	17	x		
Marcotulli	2016	P	2	2	2	0	2	2	2	2	0	14		x	
Vavla	2020	P	2	2	2	0	2	2	2	2	2	16	x		
Patel	2019	P	2	2	2	0	2	2	2	2	2	16	x		
Libri	2014	P	2	2	2	1	2	2	2	2	2	17	x		
Lynch (omav)	2023	P	2	2	2	2	2	2	2	2	2	18	x		
Lynch	2024	P	2	1	1	2	2	2	1	2	2	15	x		
Yiu	2015	P	2	2	2	0	2	2	2	2	2	16	x		
Costantini	2016	P	2	2	1	0	2	1	2	2	2	14		x	
Bolotta	2020	P	2	2	1	2	2	2	1	2	2	16	x		
Arpa	2013	P	2	2	2	0	2	1	2	2	2	15	x		
Herrero	2011	NP	2	2	2	0	2	2	2	2	2	16	x		
Rance	2010	NP	2	2	1	2	2	2	2	2	0	15	x		
Ciancarelli	2011	NP	2	2	1	0	2	2	2	2	1	14		x	
Paparella	2023	NP	2	2	2	0	2	2	2	2	2	16	x		
Brandsema	2010	P	2	2	1	0	2	2	2	2	2	15	x		
													20	4	0

Table S7: *Risk of bias assessement performed for case reports for FA-only population, n = 21 [29]. The answers have been standardized and presented in the color manner according to the literature [28]:* ***≥75% "Yes" answers = low bias - high quality; 50–74% = moderate bias; <50% = high bias.***

Study identification			Rating in each category 2 points per category (2 - yes, 1 - not full information, 0 - no information)									Bias assessment, min to include 60% (10/16)		
Author	Year	Intervention category (internal question) P/NP	Demographic characteristics	Disease history	Current clinical condition description	Diagnostic tests and assessment	Intervention and treatment procedure	Post-intervention	Adverse and unanticipated events	Take away lesson	Final result, max 16	low (16-13)	moderate (12 - 9)	high (<8)
Costantini	2013	P	2	2	2	2	2	2	2	2	16	x		
Berntsson	2013	P	2	2	2	2	2	2	2	2	16	x		
Peter	2016	P	2	2	2	2	1	2	0	2	13	x		
Kalyvas	2018	P	2	2	2	2	2	2	2	2	16	x		
Garcia Ron	2020	P	2	2	2	2	2	2	2	2	16	x		
Tekin and Levent	2022	P	2	2	2	2	2	2	2	2	16	x		
Yoon	2012	NP	2	2	2	2	2	2	2	2	16	x		
Frewin	2013	NP	2	2	2	2	2	2	2	2	16	x		
Ivak	2016	NP	2	2	2	2	2	2	2	2	16	x		
Anderson	2016	NP	1	2	2	2	2	2	2	2	15	x		
Teixera	2018	NP	2	1	1	1	2	2	0	2	11		x	
Portaro	2019	NP	2	2	2	2	2	2	2	2	16	x		
Valero	2022	NP	2	2	2	2	2	2	2	2	16	x		
Samardzic	2023	NP	2	2	2	2	2	2	0	2	14	x		
Battal	2023	NP	2	2	2	2	2	2	0	2	14	x		
Batista	2025	NP	1	1	2	2	2	2	0	2	12		x	
McGarrel	2025	NP	2	0	2	2	2	1	2	2	13	x		
Hayiroglu	2025	NP	1	1	2	2	2	2	0	2	12		x	
De Freitas	2015	NP	2	1	2	2	2	2	0	2	13	x		
McCormick	2017	NP	2	2	2	1	2	2	1	2	14	x		
Leighton	2013	NP	1	1	2	2	2	2	2	2	14	x		
												18	3	0

Table S8: *Risk of bias assessement performed for case series n = 2 for FA-only population, [30]. The answers have been standardized and presented in the color manner according to the literature [28]:* ***≥75% "Yes" answers = low bias - high quality; 50–74% = moderate bias; <50% = high bias.***

Study identification			Rating in each category 2 points per category (2 - yes, 1 - not full information, 0 - no information)											Bias assessment, min to include 60% (12/16)		
Author	Year	Intervention category (internal question)	Inclusion	Measurement of a disease	Disease identification in all participants	Consecutive inclusion of participants	Complete inclusion of participants	Reporting of demographics	Reporting of clinical information	Outcomes and follow-up results	Study sample description	Statistical analysis	Final result, max 20	low (20-16)	moderate (15 - 11)	high (<10)
Ciancarelli	2010	NP	2	2	2	2	2	2	2	2	1	2	19	x		
Elincx-Benizri	2015	P	2	2	1	0	1	2	2	2	0	2	14		x	
														1	1	0

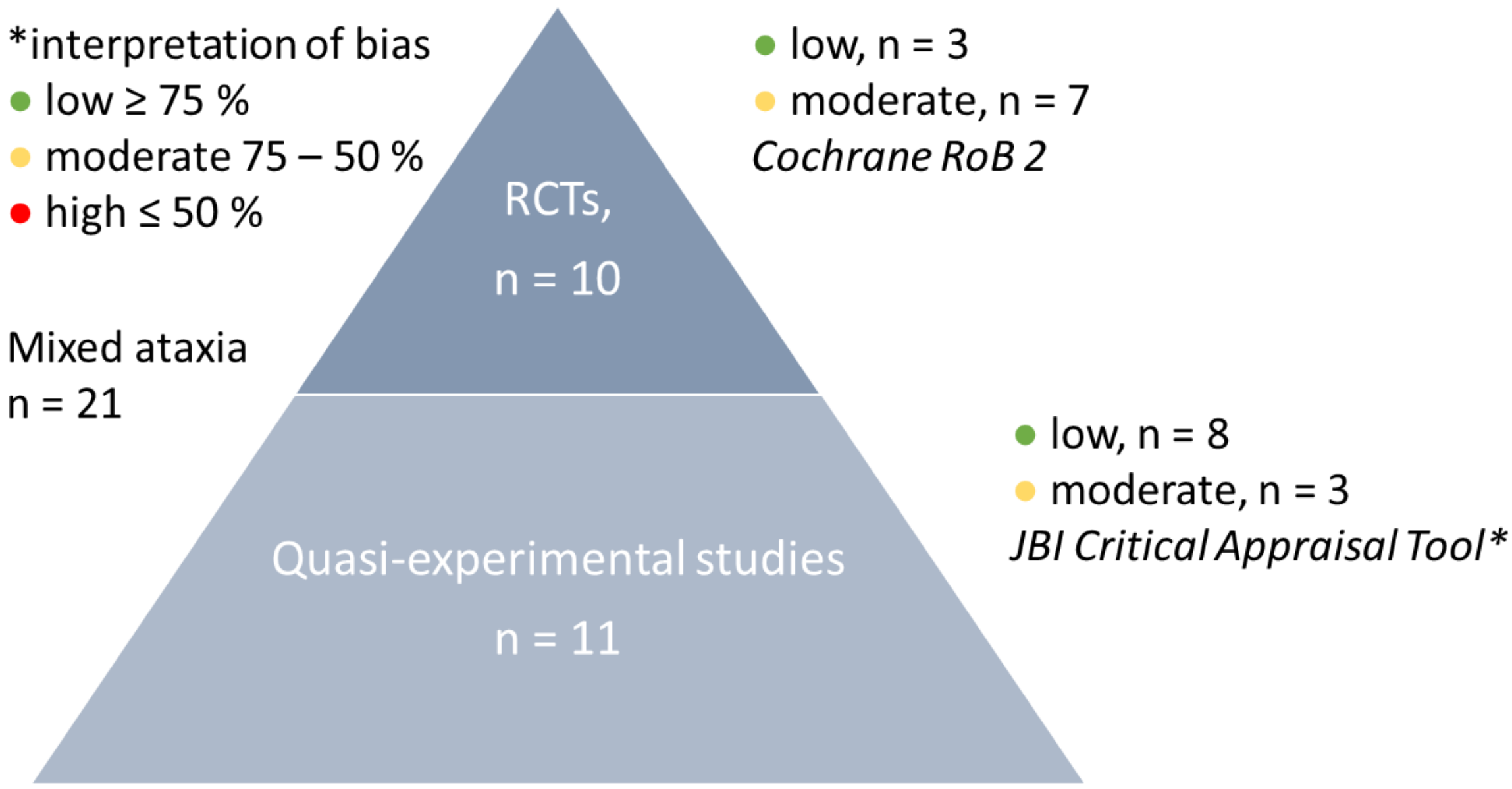

Figure S3: *Risk of bias assessment performed for all included studies for mixed-ataxia population (n = 21) using certain tools depending on the study design. *The answers from JBI Critical Appraisal Tool have been standardized [28] and presented in the color manner.*

Table S9: *Risk of bias assessment for Randomized Controlled Trials in mixed-ataxia population, n = 10, Cochrane RoB-2 [26].*

Study ID	Experimental	Comparator	Outcome	Weight	D1	D2	D3	D4	D5	Overall
Ristori 2010	riluzole	placebo	ICARS,	1	+	!	+	+	+	+
Romano 2015	riluzole	placebo	SARA, SF-36, BDI	1	+	!	!	+	+	!
Yigit 2022	rehabilitation (functional trunk training)	standard trunk control training	ICARS, TIS, modified functional reach test, 9-HPT, QDASH	1	!	!	+	!	+	!
Cabanas Valdes 2024	Home-base core stability exercise	standard care	SARA, S-TIS 2.0, ABC, 4-MWT, EQ-5D-5L,	1	+	!	+	+	+	!
Romano 2022	IMU-Based Exergame	control group	9-HPT, SARA, T25FW	1	!	+	+	+	!	!
Benussi 2015	Cerebellar tDCS	sham stimulation	SARA, ICARS, 9-HPT, 8MW,	1	!	+	+	+	!	!
Benussi 2017	Cerebellar tDCS	sham stimulation	SARA, ICARS, 9-HPT, 8MW, SAQOL-39, CBI	1	!	+	+	+	!	!
Benussi 2018	Cerebello-spinal tDCS (cathodal)	sham stimulation (anodal), cross-over, 3 months wash out	SARA, ICARS, 9-HPT, 8MW, SF-36, CBI	1	!	+	+	+	+	!
Benussi 2021	Cerebello-spinal tDCS	Sham stimulation	SARA, ICARS, CCASS, SF-36, CBI	1	!	+	+	+	+	+
Brito 2024	Cerebellar tDCS + gait training	sham stimulation + gait training	TUG, MiniBESt, SARA, PGIC	1	+	+	+	+	+	+

+ Low risk
! Some concerns
- High risk

D1 Randomisation process
D2 Deviations from the intended interventions
D3 Missing outcome data
D4 Measurement of the outcome
D5 Selection of the reported result

Table S10: *Risk of bias assessment for quasi-experimental studies in mixed-ataxia population, n = 11, JBI Critical Appraisal Tool [27]. The answers have been standardized and presented in the color manner according to the literature [28]:* ***≥75% "Yes" answers = low bias - high quality; 50–74% = moderate bias; <50% = high bias.***

Study identification			Critical appraisal (yes 2, no 0, unclear 1, not applicable 9)									Bias assessment, min to include 60% (10/16)			
			1	2	3	4	5	6	7	8	9				
Author	Year	Intervention category (internal question) P/NP	Clarity of intervention and outcome	Baseline characteristics equivalence	Consistency of intervention	Study design strength	Measurement quality over time	Attrition bias	Measurement reliability	Instrument/ tool validity	Validity of conclusions	Sum	low (18-15)	moderate (14 - 10)	high (<9)
Berntsson	2019	P	2	2	0	0	2	2	2	2	1	13		x	
Blankenberg	2012	P	2	2	2	0	2	2	2	2	2	16	x		
Ilg	2010	NP	2	2	1	0	2	2	2	2	2	15	x		
Jorge-Rodríguez	2013	NP	2	2	1	0	2	2	2	2	2	15	x		
Ilg	2012	NP	2	2	1	0	2	2	2	2	2	15	x		
Schatton	2017	NP	2	2	1	0	2	2	2	2	2	15	x		
Lowit	2020	NP	2	2	1	0	2	2	2	2	2	15	x		
Lowit	2023	NP	2	2	1	0	2	2	2	2	2	15	x		
Knuijt, Nonnekes	2024	NP	2	2	2	0	2	2	0	2	2	14		x	
Leonardi	2017	NP	2	2	1	0	2	2	2	2	2	15	x		
Schirinzi	2018	NP	2	0	1	0	2	2	2	2	2	13		x	
													8	3	

Table S11: *Patient-centered outcome measures and their effects on certain health domains and subdomains in FA-only studies, n = 280 assessments from 69 studies.*

Domain	Subdomain	Pharmacological interventions			Non-pharmacological interventions			Studies (nr/%)	Assess. (nr/%)	Studies (nr/%)	Asses. (nr/%)
		RCTs	QE	CR	RCTs	QE	CR				
Physical health	Fatigue	[31] A0001 (+) ns<br>[32] idebenone 0 ns<br>[33] deuterated linoleic acid (RT001) 0 ns<br>[34] interferon gamma 0 ns	[35] interferon gamma 0 ns<br>[36] methylprednisolone 0 ns<br>[37] thiamine 0 ns	[38] thiamine (+)				8<br>11.6%	8<br>2.9%	54<br>78.3%	118<br>42.1%
	Mobility and gait	[39] luvadaxistat 0 ns<br>[40] vatiquinone 0 ns<br>[41] deferiprone 0 ns<br>[34] interferon gamma 0 ns<br>[42] linoleic acid 0 ns<br>[33] linoleic acid as T1Mw 0 ns, as GST 0 ns<br>[43] omaveloxolone 0 ns<br>[44, 45] omaveloxolone 0 ns<br>[31] A0001 0 ns	**[36] methylprednisolone** 0 ns, **but (+)* in children**<br>[46] calcitriol (-)*<br>[47] epicatechin (+) 33%<br>[48] nicotinamide 0 ns<br>[49] resveratrol 0 ns<br>[35] interferon gamma 0 ns	[50] interferon gamma 0			[51] Scoliosis surgery (-)	17<br>24.6%	17<br>6.1%		
	Coordination and balance			[52] IGF-1 (+)	[53] rehabilitation (+) ns for ambulant patients			2<br>2.9%	2<br>0.7%		
	Sleeping			[54] Baclofen (+)				1<br>1.4%	1<br>0.4%		
	Falls	[44, 45] Omaveloxolone (+) ns						1<br>1.4%	1<br>0.4%		
	Spasms			[55] Baclofen (+)<br>[54] Baclofen (+)				2<br>2.9%	2<br>0.7%		
	Upper extremity, manual dexterity	[39] luvadaxistat 0 ns<br>[32] idebenone 0 ns<br>[56] leriglitazone 0 ns<br>**[57] epoetin alfa (+)***<br>[40] vatiquinone 0 ns<br>[41] deferiprone 0 ns<br>[58] erythropoietin 0 ns<br>[31] A0001 0 ns	[35] interferon gamma 0 ns<br>[36] methylprednisolone 0 ns<br>[46] calcitriol 0 ns<br>[47] epicatechin (+)<br>[48] nicotinamide 0 ns	[59] deferiprone (+)<br>[50] interferon gamma 0	[60] multimodal rehabilitation 0 ns	[61] occupational therapy 0 ns	[51] scoliosis surgery 0	21<br>30.4%	21<br>7.5%		

		[34] interferon gamma 0 ns<br>[43] omaveloxolone 0 ns<br>[44, 45] omaveloxolone 0 ns	[49] resveratrol 0 ns								
	Pain			[54] Baclofen (+)			[51] scoliosis surgery (+)	2<br>2.9%	2<br>0.7%		
	Vision	[40] vatiquinone 0 ns<br>[39] luvadaxistat 0 ns<br>[41] deferiprone (-) ns (20 and 40 mg doses)<br>[43] omaveloxolone 0 ns<br>[34] interferon gamma 0 ns	[49] resveratrol 0 ns<br>[35] interferon gamma (-) ns	[62] memantine (+)				8<br>11.6%	8<br>2.9%		
	Hearing					[63] **auditory device (+)***	[64] bilateral cochlear implantation (+)	2<br>2.9%	2<br>0.7%		
	Speech	**[32] idebenone (+)***	[48] nicotinamide 0 ns<br>[46] calcitriol 0 ns<br>**[49] resveratrol (+)* in high doses**	[38] thiamine (+)			[65] speech therapy (+)	6<br>8.7%	6<br>2.2%		
	General physical functioning	[39] luvadaxistat 0 ns	[66] exenatide as INAS 0 ns			[67] whole-body vibration (+)<br>[61] occupational therapy (-)*	[68] multimodal rehabilitation (dance and somatic education (+)<br>[51] scoliosis surgery (+)<br>[69] rehabilitation (+)	7<br>10.1%	7<br>2.5%		
	Cardiac function	[70] idebenone 0 ns<br>[71] idebenone 0 ns in ECG and echo<br>[57] epoetin alfa 0 ns<br>**[41] deferiprone echo LV (+)*,** LVSF, LVEF 0 ns<br>[72] etravirine 0 ns<br>[56] leriglitazone 0 ns<br>[40] vatiquinone 0 ns	**[73] interferon gamma ECG (+)*,** echo 0 ns<br>[37] thiamine +/-<br>[74] IGF-1 0 ns<br>[75] triple therapy with darbepoetin alfa, idebenone, and riboflavin +/-<br>[47] epicatechin 0 ns	[50] interferon gamma (-)<br>[52] IGF-1 (+)<br>[77] epoetin alfa 0 ns<br>[59] deferiprone (+)			[78] cardiac transplantation (+)<br>[79] cardiac transplantation (+)<br>[80] cardiac transplantation (+)<br>[81] septal myectomy (+)	23<br>33.3%	23<br>8.2%		

			[49] resveratrol 0 ns<br>[76] tocotrienol cardiac MRI (+) ns				[82] catheter ablation (+)				
	Exercise capacity	[70] idebenone 0 ns<br>[43] omaveloxolone 0 ns<br>[44, 45] omaveloxolone (+) ns<br>**[72] etravirine (+)***<br>[33] linoleic acid 0 ns<br>**[42] linoleic acid (+)***						6<br>8.7%	6<br>2.1%		
	Aerobic capacity	[70] idebenone 0 ns<br>[42] linoleic acid (+) ns<br>[33] linoleic acid 0 ns<br>[57] epoetin alfa (+) ns<br>[72] etravirine 0 ns						5<br>7.2%	5<br>1.8%		
	Dysphagia		[37] thiamine (+)					1<br>1.4%	1<br>0.4%		
	Dysmetria			[38] thiamine (+)				1<br>1.4%	1<br>0.4%		
	Neuromuscular function			[37] thiamine (+) as deep tendon reflexes		**[67] whole body vibration (+)* as circulation in muscles and (+)* as neuromuscular activity**	[83] occupational therapy 0 (strength and range of motion)<br>[84] Neuromuscular electrical stimulation (+) as muscle endurance and (+) muscle response to single stimulus)	4<br>5.8%	4<br>1.4%		
	Scoliosis						[51] scoliosis surgery (+)	1<br>1.4%	1<br>0.4%		
	Pes cavus 0							0<br>0%	0<br>0%		

Overall health	Quality of life	[31] A0001 (-) ns/ 0 ns (low/ high dose)<br>[57] epoetin alfa 0 ns<br>**[72] etravirine in vitality (+)***<br>[34] interferon gamma nd<br>[56] leriglitazone 0 ns<br>[58] erythropoietin QoL 0 ns<br>[43] omaveloxolone 0 ns	**[46] calcitriol 0 ns (+)* (reported health transition subscale)**<br>[85] idebenone (+) ns (especially social and emotional), physical (-)*<br>[74] IGF-1 3/5 fair satisfaction<br>[35] interferon gamma 0 ns<br>[36] methylprednisolone 0 ns<br>[49] resveratrol 0 ns<br>[75] triple therapy with darbepoetin alfa, idebenone, and riboflavin 44.4% felt better	[59] deferiprone 1/5 (+)	[86] rehabilitation 0 ns		[64] bilateral cochlear implantation (+) in cochlear implantation users<br>General QoL 0 (after unilateral)<br>[78] cardiac transplantation (+)	18<br>26.1%	18<br>6.4%	30<br>43.5%	42<br>15.0%
	Activities of daily living	[56] leriglitazone 0 ns<br>[39] luvadaxistat 0 ns<br>[41] deferiprone 0 ns (20mg); (-)* (40mg)<br>[31] A0001 0 ns<br>**[44, 45] omaveloxolone (+)***<br>[34] interferon gamma 0 ns<br>[57] epoeitin alfa 0 ns<br>[87] idebenone 0 ns	[66] exenatide 0 ns<br>[48] nicotinamide (+) ns<br>[36] methylprednisolone 0 ns<br>[35] interferon gamma 0 ns<br>[85] idebenone (+) ns		**[53] Rehabilitation as FAIS Impact on movement (+)* ADL 0 ns**			14<br>20.3%	14<br>5.0%		
	General well-being	[72] Etravirine (as WHODAS mobility, self-care, life activities) (+)	[49] Resveratrol 0 ns (as FAIS)	[49, 72]				3<br>4.3%	3<br>1.1%		
	Independence		[46] Calcitriol 0 ns		[86] rehabilitation 0 ns<br>[53] rehabilitation 0 ns	**[61] occupational therapy (+)* (Barthel index, Katz index)**	[69] rehabilitation (+)<br>[88] multimodal rehabilitation (+)<br>[83] occupational therapy (+)	7<br>10.1%	7<br>2.5%		
Mental health	Depression					[89] Cognitive therapy 0 ns		1<br>1.4%	1<br>0.4%	4<br>5.8%	5<br>1.8%

	Cognitive function				**[90] Anodal tDCS (+)* (as CCASS)**	**[89] Cognitive therapy 0 ns (except short term memory, cognitive flexibility and inhibition, where (+)***		2<br>2.9%	2<br>0.7%		
	Energy - vitality	**[72] Etravirine (+)* (as subdomain of SF-36)**		[52] IGF-1 (+)<br>[52, 72][51, 71]				2<br>2.9%	2<br>0.7%		
Social health	Hearing					[63] **auditory device (+)***	[64] **bilateral cochlear implantation (+)**	2<br>2.9%	2<br>0.7%	8<br>11.6%	8<br>2.9%
	Speech	**[32] idebenone (+)***	[48] nicotinamide 0 ns<br>**[49] Resveratrol (high dose) (+)***<br>[46] calcitriol 0 ns	[38] thiamine (+)			[65] speech therapy (+)	6<br>8.7%	6<br>2.1%		
	Interpersonal functioning 0							0<br>0%	0<br>0%		
Clinical status	Patient-reported impression of disease	[32] idebenone 0 ns<br>[44, 45] omaveloxolone (+) ns<br>[39] luvadaxistat 0 ns<br>[34] interferon gamma nd<br>[56] leriglitazone nd	**[36] methylprednisolone (+)* in overall population**		**[53] Rehabilitation (+)***			7<br>10.1%	7<br>2.5%	54<br>78.3%	103<br>36.8%
	Clinically reported impression of disease	[32] idebenone 0 ns<br>[34] interferon gamma 0 ns<br>[39] luvadaxistat 0 ns<br>[56] leriglitazone 0/ nd<br>[44, 45] omaveloxolone (+) ns<br>[91] carbamylated erythropoietin 0 ns<br>[31] A0001 (+) ns<br>[33] linoleic acid 0 ns	[36] methylprednisolone 0 ns					9<br>13.0%	9<br>3.2%		
	Molecular markers	[34] Interferon gamma frataxin level (whole blood and buccal samples) (+) ns	**[35] interferon gamma frataxin level buccal cells and red blood cells (+)***				[84] Neuromuscular	17<br>24.6%	17<br>6.1%		

		[58] erythropoietin frataxin level 0 ns<br>[57] epoetin alfa frataxin level in PBMCs 0 ns<br>[91] carbamylated erythropoietin Frataxin level in PBMCs and buccal cells, and mRNA 0 ns,<br>oxidative stress 0 ns<br>[72] etravirine Frataxin levels in PBMCs, mRNA nd	(platelets and whole blood (-)*)<br>[92] interferon gamma frataxin level 0 ns<br>[93] **erythropoietin frataxin level (+)***<br>**Iron metabolism (+)***<br>[37] thiamine FXN gene expression 0 ns<br>**[77] epoetin alfa frataxin level (+) gradual increase Total iron (+)*, ferritin (+)***<br>**[46] calcitriol frataxin level (+)***<br>[66] GLP-1 analogues frataxin level (+)<br>[36] methylprednisolone frataxin level 0 ns<br>[48] **nicotinamide dose-response relation with frataxin protein concentration*,**<br>sustained frataxin upregulation<br>[49] resveratrol Frataxin level in PBMC (+) ns;<br>Frataxin mRNA 0 ns;<br>**Oxidative stress 0 ns/ (+)* (low/high dose)**<br>[73] interferon gamma frataxin level in PBMC (+) ns				electrical stimulation<br>Mitochondrial capacity 0				
	Neuroimaging	[56] leriglitazone (spinal cord) 0 ns	[47] epicatechin (spinal cord) 0 ns<br>[94] **erythropoietin (white matter anisotropy) (+)***					4<br>5.8%	4<br>1.4%		

			[95] erythropoietin<br>**VBM gray matter volume (+)***								
	Cerebellar function	[56] Leriglitazone (as cerebellar dysfunction of upper limb) (-) ns			[90] **anodal cerebellar tDCS as CCASS (+)***	[61] occupational therapy as Klockgether scale 0 ns	[96] robotic gait training and tDCS as CBI (+)	4<br>5.8%	4<br>1.4%		
	General ataxia severity	[87] idebenone as ICARS (+) ns<br>[97] idebenone as ICARS total (subscores speech, posture and stance (-)*)- 12 months open label;<br>**ICARS total (+) ns in all subscales (+)*** exept posture and stance (-)* - 18 months (6 month RCT + open label)<br>**[32] idebenone as ICARS (+) ns;**<br>**ICARS (+)* for ambulatory patients**,<br>[58] erythropoietin as SARA 0 ns<br>[57] epoetin alfa as SARA 0 ns<br>[41] deferiprone as ICARS 0 ns/ (-)* (low/high dose)<br>[91] carbamylated erythropoietin as SARA 0 ns<br>**[72] etravirine as SARA (+)* after 2 and 4 months**<br>[56] leriglitazone as SARA 0 ns	[85] idebenone as ICARS 0 ns<br>[92] interferon gamma as SARA 0 ns<br>[93] erythropoietin as SARA 0 ns<br>[94] erythropoietin SARA 7/9 (+) (at least -2 points); FARS 7/9 (+) (at least -4 points);<br>No correlation between DTI changes and changes in SARA.<br>[95] erythropoietin SARA improvement correlated with VBM changes<br>**[37] thiamine as SARA (+)***<br>[74] IGF-1 as SARA 2/5 (+)/ (-)<br>[77] epoetin alfa ICARS 0 ns (at frataxin peak 0 ns)<br>[46] calcitriol as SARA (-) ns<br>[75] triple therapy with darbepoetin alfa, idebenone, and riboflavin as SARA 0 ns (2 years pre and post); (+) vs annual worsening rate	[38] thiamine as SARA (+) in two patients: 58.9 %, 66.7 %<br>[52] IGF-1 SARA total (+) (9 points), stable over 36 months<br>[59] deferiprone as SARA 2/5 (+)	**[86] rehabilitation (+)* as ICARS**<br>**[90] anodal cerebellar tDCS (+)* SARA**	[60] **multimodal rehabilitation (+)* as SARA**	[96] robotic gait training and tDCS (+) as SARA<br>[69] rehabilitation (+) as SARA<br>[88] multimodal rehabilitation (+) as SARA<br>[79] cardiac transplantation as neurological function (+)<br>[80] heart transplantation as neurological function (+)	35<br>50.7%	35<br>12.5%		

			[66] GLP-1 analogues as SARA 0 ns<br>[48] nicotinamide as SARA 0 ns, as SCAFI 0 ns<br>**[49] resveratrol as SARA (+) ns; ICARS /(+)*(mostly in posture)**<br>[76] tocotrienol as ICARS (-) ns<br>**[73] interferon gamma as SARA – stabilised *** worsening trend after the discontinuation								
	FA-specific severity	[87] idebenone as FARS (+) ns, as FACT-Z3 0 ns<br>[97] idebenone as FARS total (-)* **Subscore speech (+)*** - 12 months<br>FARS (-)*, subscales **speech, lower limb coordination (+)* -** 18 months (6 months RCT + open label)<br>[41] deferiprone as FARS 0 ns/ (-)* (low/high dose)<br>[91] carbamylated erythropoietin as FARS 0 ns<br>[34] interferon gamma as FARS 0 ns, mFARS 0 ns 6 months + open label; mFARS (+) ns after 52 weeks extention<br>**[43] omaveloxolone as mFARS (+)***<br>**[44, 45] omaveloxolone as mFARS (+)*** subscores: upper limb coordination and upright stability;	[94] erythropoietin FARS 7/9 (+) (at least -4 points) No correlation between DTI changes and changes in FARS.<br>[95] erythropoietin FARS improvement correlated with VBM changes<br>**[35] interferon gamma as FARS total (+)*, mainly upper extermities subscale**<br>[47] epicatechin as FARS/mFARS (+) ns; FARS 56% (+)<br>[36] methylprednisolone as FARS 0 ns; as mFARS 0 ns<br>**[49] resveratrol FARS (+)* (mostly upper limb and bulbar components**)	[59] deferiprone as FARS 3/5 (+)<br>[50] interferon gamma as FARS (+)	[53] rehabilitation 0 ns	[60] **multimodal rehabilitation as FARS (+)***	[78] cardiac transplantation (-)<br>[99] cardiac transplantation (+) then gradually (-)	27<br>39.1%	27<br>9.6%		

		**[98] omaveloxolone 3 years propensity matched mFARS (+)***<br>[42] linoleic acid as FARS Neuro (+) ns<br>**[33] linoleic acid as mFARS (+)***<br>**[31] A0001 as FARS (+)* / (+)** (FARS subscore lower limb (+)* for a high dose)**<br>[91] carbamylated erythropoietin as FARS 0 ns<br>[39] luvadaxistat as mFARS-Neuro (+) ns<br>**[40] vatiquinone:**<br>**FARS-Neuro 0 ns;**<br>**FARS-Neuro (+)* 73% (vs 43) ≥ 3 points, 64% (vs 33) ≥ 5 points;**<br>**FARS (+)* vs natural history data**									
Caregiver	Satisfaction with the intervention						[51] Scoliosis surgery (+)	1<br>1.4%	1<br>0.4%	4<br>5.8%	4<br>1.4%
	Quality of life of FA patient	[34]<br>Interferon gamma nd	[35] Interferon gamma 0 ns					2<br>2.9%	2<br>0.7%		
	Functional assessment of FA patient		[48] Nicotinamide (+) ns (as FARS ADL)					1<br>1.4%	1<br>0.4%		
	Burden/well-being of a caregiver							0<br>0%	0<br>0%		
Economic	Economic assessment 0							0<br>0%	0<br>0%	0<br>0%	0<br>0%

Table S12: *Patient-centered outcome measures and their effects on certain health domains and subdomains in mixed-ataxia studies, n = 71 assessments from 21 studies.*

Domain	Subdomain	Pharmacological interventions			Non-pharmacological interventions			Studies (nr/%)	Assess. (nr/%)	Studies (nr/%)	Asses. (nr/%)
		RCTs	QE	CR	RCTs	QE	CR				
Physical health	Fatigue			[6] Baclofen (+)		[12] Speech therapy 0 ns<br>[13] Speech therapy 0 ns		3<br>14.3%	3<br>4.2%	17<br>81.0%	34<br>47.9%
	Mobility and gait				**[19] rehabilitation as 4-MWT (+)* after 5 weeks; 0 ns after 10 weeks**<br>**[7] cerebellar tDCS as 8MW (+)***<br>[8] cerebellar tDCS as 8MW 0 ns<br>**[9] cerebello-spinal tDCS as 8 MW (+)* after 2 weeks/1 month/3 months**<br>**[11] cerebellar tDCS + gait training as TUG (+)***<br>[15] video games as T23FW 0 ns	**[16] video game as gait analysis (+)* lateral sway, step length, and goal-directed leg placement**<br>**[21] wearable proprioceptive stabilizer for rehabilitation as 6-MWT (+)***<br>[22] non-invasive focal mechanical vibrations via wearable devices as 6-MWT (+) ns<br>[20] coordinative training 0		10<br>47.6%	10<br>14.1%		
	Balance, Postural stability, trunk control				[19] rehabilitation 0 ns<br>[11] cerebellar tDCS + gait training as miniBest 0 ns<br>**[18] rehabilitation as TIS 0 ns; as MFRT (+)***	**[16] video games as DGI (+)***<br>**[20] rehabilitation as BBS (+)* after the intervention**<br>**[17] video games (+)* as body sway with eyes closed; 0 ns with eyes open**		6<br>28.6%	6<br>8.5%		
	Falls				**[20] rehabilitation (+)* after the intervention**			1<br>4.8%	1<br>1.4%		
	Spasms			[6] Baclofen (+)				1<br>4.8%	1<br>1.4%		
	Upper extremity, manual dexterity				**[7] cerebellar tDCS (+)***<br>**[8] cerebellar tDCS (+)* for dominant and non dominant hand after 2 weeks and 1 month**	**[21] wearable proprioceptive stabilizer for rehabilitation (+)* for dominant hand**, 0 ns for non dominant hand<br>[22] non-invasive focal		7<br>33.3%	7<br>9.9%		

					**[9] cerebello-spinal tDCS (+)* for dominant and non-dominant hand for 2 weeks/1 month/3 months**<br>**[15] IMU-based exergame (+)* for dominant hand, 0 ns for non dominant hand**<br>[18] rehabilitaion 0 ns	mechanical vibrations via wearable devices (+) ns for dominant hand, 0 ns for non dominant hand					
	Pain			[6] Baclofen (+)				1<br>4.8%	1<br>1.4%		
	Speech					**[12] Speech therapy (+)* (as voice quality, prolonged vowel duration)**<br>**[13] Speech therapy (+)* (as vocal strain, reading inteligibility)**<br>**[14] speech therapy (+)* (as speaking rate, mean pause duration)**<br>**[21] wearable proprioceptive stabilizer (focal mechanical vibration) + physiotherapy (+)***		4<br>19.0%	4<br>5.6%		
	General physical functioning		[5] vatiquinone (+)					1<br>4.8%	1<br>1.4%		
Overall health	Quality of life	**[4] riluzole (+)* for social role functioning**	[5] vatiquinone (+)		**[19] rehabilitation (+)***	**[8] cerebellar tDCS (+)* only after 1 week**<br>**[9] cerebello-spinal tDCS (+)* after 2 week/1 months/3 months**<br>**[10] cerebello-spinal tDCS (+)* after 2/ 12 weeks, up to 52 weeks**		6<br>28.6%	6<br>8.5%	7<br>33.3%	7<br>9.9%
	General well-being			[6] Baclofen (+)				1<br>4.8%	1<br>1.4%		

Mental health	Depression	[4] riluzole 0 ns						1<br>4.8%	1<br>1.4%	2<br>9.5%	2<br>2.8%
	Cognitive function				**[10] tDCS (+)* after 52 weeks**			1<br>4.8%	1<br>1.4%		
Social health	Speech					**[12] Speech therapy (+)* (as voice quality, prolonged vowel duration)**<br>**[13] Speech therapy (+)* (as vocal strain, reading inteligibility)**<br>**[14] speech therapy (+)* (as speaking rate, mean pause duration)**<br>**[21] wearable proprioceptive stabilizer (focal mechanical vibration) + physiotherapy (+)***		4<br>19.0%	4<br>5.6%	4<br>19.0%	4<br>5.6%
Clinical status	Patient-reported impression of disease				[11] tDCS + gait training 0 ns			1<br>4.8%	1<br>1.4%	17<br>81.0%	23<br>32.4%
	Cerebellar function				**[8] cerebellar tDCS (+)* (after 1 month and 3 months)**<br>**[9] cerebello-spinal tDCS (+)* after 2 weeks/1 month/3 months**<br>**[10] cerebello-spinal tDCS (+)* after 2/12 weeks as CBI Open labe las CCASS and CBI (+)***			3<br>14.3%	3<br>4.2%		
	General ataxia severity	**[3] riluzole as ICARS (+)***<br>**[4] riluzole as SARA (+)***	[5] vatiquinone (+)		[19] rehabilitation as SARA 0 ns<br>[7] **cerebellar tDCS as SARA (+)* and ICARS (+)***<br>[8] **cerebellar DCS as SARA (+)* and ICARS (+)* (2 weeks/1 month/3 months**	**[16] video game–based coordinative training as SARA (+)***<br>**[21] wearable proprioceptive stabilizer for gait and limb ataxia rehabilitation as SARA (+)***		17<br>81.0%	17<br>23.9%		

					[9] **cerebello-spinal tDCS as SARA (+)* and ICARS (+)* (2 weeks/1 month/3 months**<br>[11] **cerebellar tDCS + gait training as SARA (+)* in intervention group**<br>[15] IMU-based exergame upper body rehabilitation as SARA 0 ns<br>[18] rehabilitation as ICARS 0 ns<br>[10] **cerebello-spinal tDCS as SARA (+)*, ICARS (+)* up to 52 weeks**<br>[23] **multidisciplinary rehabilitation (+)* as ICARS 28 and 42 days**	[22] non-invasive focal mechanical vibrations via wearable devices as SARA (+) ns<br>**[20] coordinative training as SARA (+)* after 4 week intervention** ((-)* 1 year follow up)<br>**[17] individualized exergame training for postural control as SARA (+)***					
	Progress in individual goal					[20] rehabilitation (+)<br>**[17] video games (+)***		2<br>9.5%	2<br>2.8%		
Caregiver	Satisfaction with the intervention					[14] speech therapy 0 ns		1<br>4.8%	1<br>1.4%	1<br>4.8%	1<br>1.4%
Economic	Economic assessment							0<br>0%	0<br>0%	0<br>0%	0<br>0%

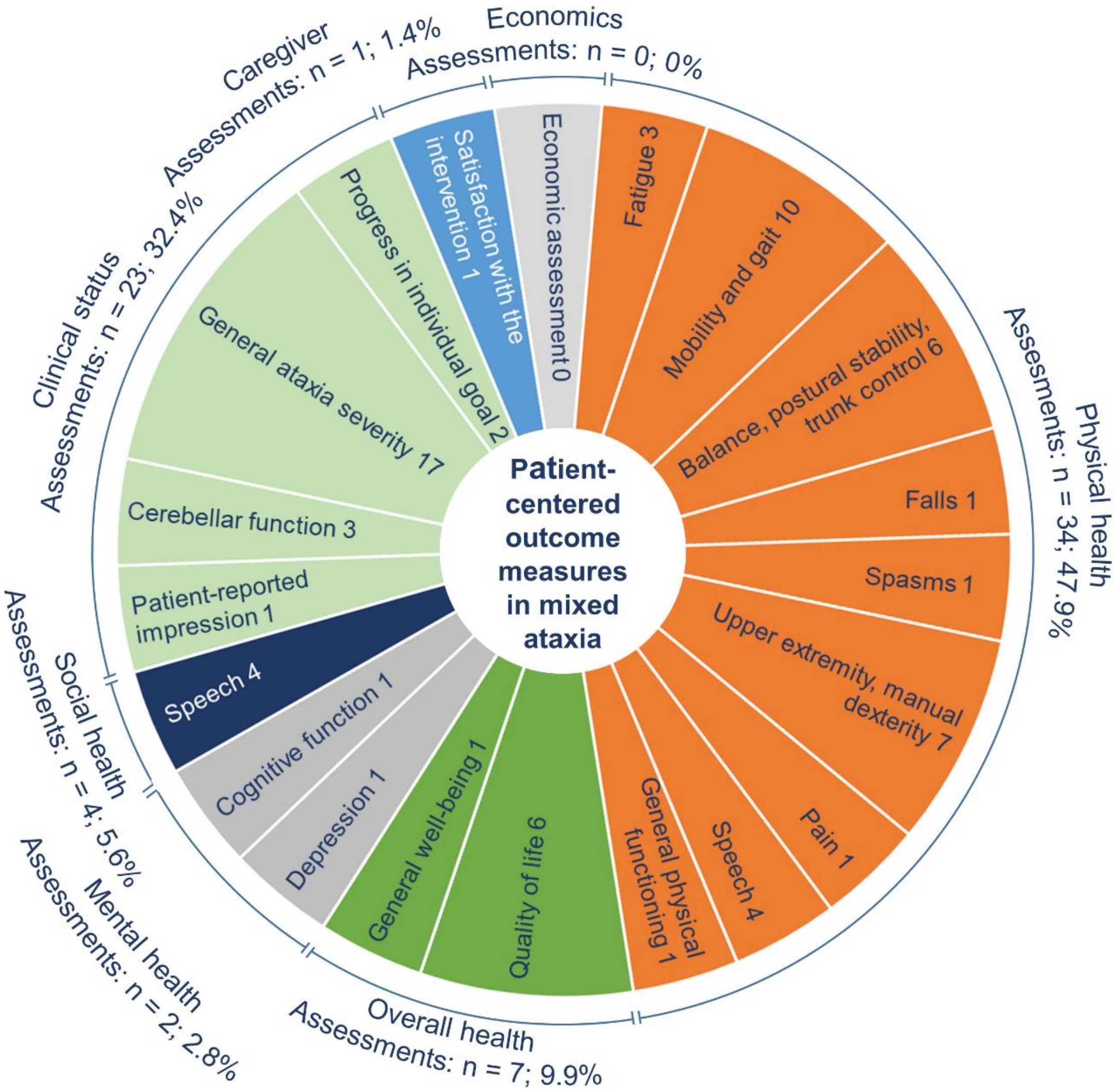

Figure S4: *Patient-centered outcome measures used in the included studies; n = 71 assessments from 21 studies for mixed-ataxia patients and the division in domains and subdomains.*

References:

1. Page, M.J., et al., *The PRISMA 2020 statement: an updated guideline for reporting systematic reviews.* BMJ, 2021. **372**: p. n71.
2. Higgins JPT, T.J., Chandler J, Cumpston M, Li T, Page MJ, Welch VA, *Cochrane Handbook for Systematic Reviews of Interventions version 6.5 (updated August 2024)*. 2024: Cochrane.
3. Ristori G., R.S., Visconti A., Cannoni S., Spadaro M., Frontali M., Pontieri F. E., Vanacore N., Salvetti M., *Riluzole in cerebellar ataxia: A randomized, double-blind, placebo-controlled pilot trial.* Neurology, 2010. **74**(10): p. 839–845.
4. Romano, S., et al., *Riluzole in patients with hereditary cerebellar ataxia: a randomised, double-blind, placebo-controlled trial.* Lancet Neurol, 2015. **14**(10): p. 985–91.
5. Blankenberg, F.G., et al., *Brain uptake of Tc99m-HMPAO correlates with clinical response to the novel redox modulating agent EPI-743 in patients with mitochondrial disease.* Mol Genet Metab, 2012. **107**(4): p. 690–9.
6. Berntsson, S.G., et al., *Inherited Ataxia and Intrathecal Baclofen for the Treatment of Spasticity and Painful Spasms.* Stereotact Funct Neurosurg, 2019. **97**(1): p. 18–23.
7. Benussi, A., et al., *Cerebellar transcranial direct current stimulation in patients with ataxia: A double-blind, randomized, sham-controlled study.* Mov Disord, 2015. **30**(12): p. 1701–5.
8. Benussi, A., et al., *Long term clinical and neurophysiological effects of cerebellar transcranial direct current stimulation in patients with neurodegenerative ataxia.* Brain Stimulation, 2017. **10**(2): p. 242–250.
9. Benussi, A., et al., *Cerebello-spinal tDCS in ataxia: A randomized, double-blind, sham-controlled, crossover trial.* Neurology, 2018. **91**(12): p. e1090–e1101.
10. Benussi, A., et al., *Motor and cognitive outcomes of cerebello-spinal stimulation in neurodegenerative ataxia.* Brain, 2021. **144**(8): p. 2310–2321.
11. Brito, R., et al., *Differential Effects of Cerebellar Transcranial Direct Current Stimulation with Gait Training on Functional Mobility, Balance, and Ataxia Symptoms.* Cerebellum, 2024. **23**(6): p. 2457–2467.
12. Lowit, A., A. Egan, and M. Hadjivassiliou, *Feasibility and Acceptability of Lee Silverman Voice Treatment in Progressive Ataxias.* Cerebellum, 2020. **19**(5): p. 701–714.
13. Lowit, A., et al., *ClearSpeechTogether: a Rater Blinded, Single, Controlled Feasibility Study of Speech Intervention for People with Progressive Ataxia.* Cerebellum, 2023. **22**(5): p. 865–876.
14. Knuijt, S., et al., *The Use of the Voice Trainer App for Vocal Control in People with a Degenerative Ataxia: A Pilot Intervention Study.* Cerebellum, 2024. **23**(4): p. 1490–1497.
15. Romano, A., et al., *Upper Body Physical Rehabilitation for Children with Ataxia through IMU-Based Exergame.* J Clin Med, 2022. **11**(4).
16. Ilg, W., Schatton C, Schicks J, Giese M.A, Schöls L, Synofzik M, *Video game–based coordinative training improves ataxia in children with degenerative ataxia.* Neurology, 2012. **79**(20): p. 2056–2060.
17. Schatton, C., et al., *Individualized exergame training improves postural control in advanced degenerative spinocerebellar ataxia: A rater-blinded, intra-individually controlled trial.* Parkinsonism Relat Disord, 2017. **39**: p. 80–84.
18. Yigit, S., et al., *Effectiveness of functional trunk training on trunk control and upper limb functions in patients with autosomal recessive hereditary ataxia.* NeuroRehabilitation, 2022. **51**(1): p. 41–50.
19. Cabanas-Valdes, R., et al., *Effect of a Home-Base Core Stability Exercises in Hereditary Ataxia. A Randomized Controlled Trial. A Pilot Randomized Controlled Trial.* Mov Disord Clin Pract, 2024. **11**(6): p. 666–675.
20. Ilg, W., et al., *Long-term effects of coordinative training in degenerative cerebellar disease.* Mov Disord, 2010. **25**(13): p. 2239–46.
21. Leonardi, L., et al., *A wearable proprioceptive stabilizer for rehabilitation of limb and gait ataxia in hereditary cerebellar ataxias: a pilot open-labeled study.* Neurol Sci, 2017. **38**(3): p. 459–463.

22. Schirinzi, T., et al., *Non-invasive Focal Mechanical Vibrations Delivered by Wearable Devices: An Open-Label Pilot Study in Childhood Ataxia.* Front Neurol, 2018. **9**: p. 849.
23. Jorge L Jorge-Rodríguez, E.F.M., Daymi Rodríguez Pérez, Antonio Peralta-Flores, Jorge A Bergado, *Motor improvement in cerebellar ataxia after integral rehabilitation.* Journal of Neurorestoratology, 2013.
24. The National Institute of Neurological Disorders and Stroke, *Start-up Resource – NINDS Friedreich's ataxia CDE Recommendations.* 2023.
25. Lynch, D.R., et al., *Common data elements for clinical research in Friedreich's ataxia.* Mov Disord, 2013. **28**(2): p. 190–5.
26. Sterne, J.A.C., et al., *RoB 2: a revised tool for assessing risk of bias in randomised trials.* BMJ, 2019. **366**: p. l4898.
27. Barker, T.H., et al., *The revised JBI critical appraisal tool for the assessment of risk of bias for quasi-experimental studies.* JBI Evid Synth, 2024. **22**(3): p. 378–388.
28. de Smalen, A.W., et al., *Developing an evidence assessment framework and appraising the academic literature on migrant health in Malaysia: a scoping review.* BMJ Open, 2021. **11**(1): p. e041379.
29. Aromataris E, L.C., Porritt K, Pilla B, Jordan Z, editors, *JBI Manual for Evidence Synthesis.* JBI, 2024.
30. Munn, Z., et al., *Methodological quality of case series studies: an introduction to the JBI critical appraisal tool.* JBI Evid Synth, 2020. **18**(10): p. 2127–2133.
31. Lynch, D.R., et al., *A0001 in Friedreich ataxia: biochemical characterization and effects in a clinical trial.* Mov Disord, 2012. **27**(8): p. 1026–33.
32. Cook, A., et al., *Patient-reported outcomes in Friedreich's ataxia after withdrawal from idebenone.* Acta Neurol Scand, 2019. **139**(6): p. 533–539.
33. Lynch, D.R., et al., *Double blind trial of a deuterated form of linoleic acid (RT001) in Friedreich ataxia.* J Neurol, 2023. **270**(3): p. 1615–1623.
34. Lynch, D.R., et al., *Randomized, double-blind, placebo-controlled study of interferon-gamma 1b in Friedreich Ataxia.* Ann Clin Transl Neurol, 2019. **6**(3): p. 546–553.
35. Seyer, L., et al., *Open-label pilot study of interferon gamma-1b in Friedreich ataxia.* Acta Neurol Scand, 2015. **132**(1): p. 7–15.
36. Patel, M., et al., *Open-label pilot study of oral methylprednisolone for the treatment of patients with friedreich ataxia.* Muscle Nerve, 2019. **60**(5): p. 571–575.
37. Costantini, A., et al., *Long-term treatment with thiamine as possible medical therapy for Friedreich ataxia.* J Neurol, 2016. **263**(11): p. 2170–2178.
38. Costantini, A., et al., *High-dose thiamine improves the symptoms of Friedreich's ataxia.* BMJ Case Rep, 2013. **2013**.
39. Wang, H., et al., *Results of a randomized double-blind study evaluating luvadaxistat in adults with Friedreich ataxia.* Ann Clin Transl Neurol, 2021. **8**(6): p. 1343–1352.
40. Zesiewicz T., S.J.L., Perlman S., Sullivan K.L., Shaw J.D., Huang Y., Isaacs C., Gooch C., Lynch D.R., Klein M.B., *Double-blind, Randomized and Controlled Trial of EPI-743 in Friedreich's Ataxia.* Neurodegenerative Disease Management, 2018. **8**(4): p. 233–242.
41. Pandolfo, M., et al., *Deferiprone in Friedreich ataxia: a 6-month randomized controlled trial.* Ann Neurol, 2014. **76**(4): p. 509–21.
42. Zesiewicz, T., et al., *Randomized, clinical trial of RT001: Early signals of efficacy in Friedreich's ataxia.* Mov Disord, 2018. **33**(6): p. 1000–1005.
43. Lynch, D.R., et al., *Safety, pharmacodynamics, and potential benefit of omaveloxolone in Friedreich ataxia.* Ann Clin Transl Neurol, 2019. **6**(1): p. 15–26.
44. Lynch, D.R., et al., *Safety and Efficacy of Omaveloxolone in Friedreich Ataxia (MOXIe Study).* Ann Neurol, 2021. **89**(2): p. 212–225.
45. Lynch, D.R., et al., *Safety and Efficacy of Omaveloxolone in Friedreich Ataxia (MOXIe Study) - correction.* Ann Neurol, 2021. **89**(2): p. 212–225.

46. Alemany-Perna, B., et al., *Calcitriol Treatment Is Safe and Increases Frataxin Levels in Friedreich Ataxia Patients.* Mov Disord, 2024. **39**(7): p. 1099–1108.
47. Qureshi, M.Y., et al., *Safety and efficacy of (+)-epicatechin in subjects with Friedreich's ataxia: A phase II, open-label, prospective study.* J Inherit Metab Dis, 2021. **44**(2): p. 502–514.
48. Libri, V., et al., *Epigenetic and neurological effects and safety of high-dose nicotinamide in patients with Friedreich's ataxia: an exploratory, open-label, dose-escalation study.* Lancet, 2014. **384**(9942): p. 504–13.
49. Yiu, E.M., et al., *An open-label trial in Friedreich ataxia suggests clinical benefit with high-dose resveratrol, without effect on frataxin levels.* J Neurol, 2015. **262**(5): p. 1344–53.
50. Tekin, H.G. and E. Levent, *Neurological Recovery with Interferon-gamma Treatment in Friedreich's Ataxia.* J Coll Physicians Surg Pak, 2022. **32**(5): p. 671–673.
51. Leighton, J., Howard, J, El-Hawary R, , *Posterior Spinal Fusion for Friedreich Ataxia-Related Scoliosis in Twin Girls, A Case Report.* Journal of bone and joint surgery / Case Connector, 2013. **3**(1).
52. Garcia Ron, A. and M. Rodriguez Mesa, *Compassionate use of human recombinant insulin-like growth factor-1 therapy in Friedreich's ataxia.* Neurologia (Engl Ed), 2020. **35**(2): p. 140–143.
53. Milne, S.C., et al., *Can rehabilitation improve the health and well-being in Friedreich's ataxia: a randomized controlled trial?* Clin Rehabil, 2018. **32**(5): p. 630–643.
54. Kalyvas, A.V., et al., *Intrathecal Baclofen Therapy for Painful Muscle Spasms in a Patient with Friedreich's Ataxia.* Stereotact Funct Neurosurg, 2018. **96**(2): p. 127–130.
55. Berntsson, S.G., A. Holtz, and A. Melberg, *Does intrathecal baclofen have a place in the treatment of painful spasms in friedreich ataxia?* Case Rep Neurol, 2013. **5**(3): p. 201–3.
56. Pandolfo, M., et al., *Efficacy and Safety of Leriglitazone in Patients With Friedreich Ataxia: A Phase 2 Double-Blind, Randomized Controlled Trial (FRAMES).* Neurol Genet, 2022. **8**(6): p. e200034.
57. Sacca, F., et al., *Long-term effect of epoetin alfa on clinical and biochemical markers in friedreich ataxia.* Mov Disord, 2016. **31**(5): p. 734–41.
58. Mariotti C., F.R., Caldarazzo S., Nanetti L., Di Bella D., Plumari M., Lauria G., Cappellini M.D., Duca L., Solari A., Taroni F., *Erythropoietin in Friedreich Ataxia: No Effect on Frataxin in a Randomized Controlled Trial.* Movement Disordorders, 2012. **27**(3): p. 446–449.
59. Elincx-Benizri, S., et al., *Clinical Experience With Deferiprone Treatment for Friedreich Ataxia.* J Child Neurol, 2016. **31**(8): p. 1036–40.
60. Paparella, G., et al., *Effectiveness of rehabilitation intervention in persons with Friedreich ataxia.* Front Neurol, 2023. **14**: p. 1270296.
61. Ciancarelli I, C.V., Carolei A, *Disabilità e terapia occupazionale nei pazienti con atassia di Friedreich.* G Ital Med Lav Erg, 2011. **33**(2): p. 201–204.
62. Peter, S., et al., *[Memantine for optic nerve atrophy in Friedreich's Ataxia].* Ophthalmologe, 2016. **113**(8): p. 704–7.
63. Rance, G., et al., *Successful treatment of auditory perceptual disorder in individuals with Friedreich ataxia.* Neuroscience, 2010. **171**(2): p. 552–5.
64. Frewin B., C.M., Donnelly N., *Bilateral cochlear implantation in Friedreich's ataxia: A case study.* Cochlear Implants International, 2013. **14**(5): p. 287–290.
65. Batista, D.d.J. and A.S.d. Conceição, *Improving oral communication in a jornalism student with Friedreich ataxia: A case report.* Revista CEFAC, 2025. **27**(3).
66. Igoillo-Esteve, M., et al., *Exenatide induces frataxin expression and improves mitochondrial function in Friedreich ataxia.* JCI Insight, 2020. **5**(2).
67. Herrero, A.J., et al., *Whole-body vibration alters blood flow velocity and neuromuscular activity in Friedreich's ataxia.* Clin Physiol Funct Imaging, 2011. **31**(2): p. 139–44.
68. Teixeira, F.A.C., et al., *Friedreich's Ataxia: dance and somatic education a case report.* Motriz: Revista de Educação Física, 2018. **24**(3).

69. Battal, G., N. Pinsault, and B. Hanna-Boutros, *Intensive Multimodal Treatment for A Young Adult with Friedreich Ataxia: A Case Report.* International Journal of Physiotherapy and Research, 2023. **11**(3): p. 4508–4516.
70. Drinkard, B.E., et al., *Exercise capacity and idebenone intervention in children and adolescents with Friedreich ataxia.* Arch Phys Med Rehabil, 2010. **91**(7): p. 1044–50.
71. Lagedrost, S.J., et al., *Idebenone in Friedreich ataxia cardiomyopathy-results from a 6-month phase III study (IONIA).* Am Heart J, 2011. **161**(3): p. 639–645 e1.
72. Paparella, G., et al., *A Pilot Phase 2 Randomized Trial to Evaluate the Safety and Potential Efficacy of Etravirine in Friedreich Ataxia Patients.* Children (Basel), 2024. **11**(8).
73. Vavla, M., et al., *Safety and efficacy of interferon gamma in friedreich's ataxia.* Mov Disord, 2020. **35**(2): p. 370–371.
74. Sanz-Gallego I., T.-A.I., Arpa J., *IGF-1 in Friedreich's Ataxia – proof-of-concept trial.* Cerebellum & Ataxia, 2014. **1**(10).
75. Arpa, J., et al., *Triple therapy with darbepoetin alfa, idebenone, and riboflavin in Friedreich's ataxia: an open-label trial.* Cerebellum, 2013. **12**(5): p. 713–20.
76. Bolotta, A., et al., *Effects of tocotrienol supplementation in Friedreich's ataxia: A model of oxidative stress pathology.* Exp Biol Med (Maywood), 2020. **245**(3): p. 201–212.
77. Sacca, F., et al., *Epoetin alfa increases frataxin production in Friedreich's ataxia without affecting hematocrit.* Mov Disord, 2011. **26**(4): p. 739–42.
78. Yoon, G., et al., *Cardiac transplantation in Friedreich ataxia.* J Child Neurol, 2012. **27**(9): p. 1193–6.
79. McCormick, A., et al., *Cardiac transplantation in Friedreich Ataxia: Extended follow-up.* J Neurol Sci, 2017. **375**: p. 471–473.
80. Valero, M.J., et al., *Should Advanced Friedreich's Ataxia Be a Contraindication for Heart Transplantation? A Case Report of a Successful Procedure in a 58-Year-Old Patient.* J Cardiovasc Dev Dis, 2022. **9**(3).
81. Anderson, H.N., H.M. Burkhart, and J.N. Johnson, *Septal myectomy for hypertrophic obstructive cardiomyopathy in Friedreich's ataxia.* Cardiol Young, 2016. **26**(1): p. 175–8.
82. Hayiroglu, M.I., K. Kalenderoglu, and K. Gurkan, *Tachycardiomyopathy Treated With Ablation by Using 3D Mapping System in a Patient With Friedreich Ataxia.* Pacing Clin Electrophysiol, 2025. **48**(2): p. 227–229.
83. De Freitas Zanona A, S.A.C.A., Gomes Goncalves A.L, Da Silva M.P, Dos Santos R.N, Do Espirito Santo R.P, Dos Santos T.M.S, Da Silva M.N.S., *Intervenção Terapêutica Ocupacional junto à duas jovens com Ataxia de Friedreich:um relato de caso.* Revista Ocupación Humana, 2015.
84. McGarrell, N.T., M.E. Green, and K.K. McCully, *Muscle Endurance Training in a Person with Friedreich's Ataxia.* Muscles, 2025. **4**(1).
85. Brandsema, J.F., et al., *Intermediate-dose idebenone and quality of life in Friedreich ataxia.* Pediatr Neurol, 2010. **42**(5): p. 338–42.
86. Seco Calvo J., G.F.I., Casado Verdejol, Rodriguez Perez V., Fernandez-Atutxa A, Torres-Unda J., *Improvements in Quality of Life in Individuals with Friedreich's Ataxia after Participation in a 5-Year Program of Physical Activity: An observational Study Pre-Post Test Design, and Two Years Follow-Up.* International Journal of Neurorehabilitation, 2014. **01**(03).
87. Lynch D.R., P.S.L., Meier T., *A Phase 3, Double-blind, Placebo-Controlled Trial of Idebenone in Friedreich Ataxia.* ARCH NEUROL, 2010. **67**(8).
88. Samardzic, V., *Efficiency of a 3-week multicomponent rehabilitation on improving the function in a patient with Friedreich's ataxia: A case report.* Vojnosanitetski pregled, 2023. **80**(2): p. 182–187.
89. Ciancarelli I, C.V., Carolei A, *Evaluation of neuropsychological functions in patients with Friedreich ataxia before and after cognitive therapy* Functional Neurology, 2010. **25**(2): p. 81–85.

90. Naeije, G., et al., *Anodal Cerebellar Transcranial Direct Current Stimulation Reduces Motor and Cognitive Symptoms in Friedreich's Ataxia: A Randomized, Sham-Controlled Trial.* Mov Disord, 2023. **38**(8): p. 1443–1450.
91. Boesch, S., et al., *Safety and tolerability of carbamylated erythropoietin in Friedreich's ataxia.* Mov Disord, 2014. **29**(7): p. 935–9.
92. Marcotulli, C., et al., *GIFT-1, a phase IIa clinical trial to test the safety and efficacy of IFNgamma administration in FRDA patients.* Neurol Sci, 2016. **37**(3): p. 361–4.
93. Nachbauer, W., et al., *Effects of erythropoietin on frataxin levels and mitochondrial function in Friedreich ataxia--a dose-response trial.* Cerebellum, 2011. **10**(4): p. 763–9.
94. Egger, K., et al., *White matter changes in patients with friedreich ataxia after treatment with erythropoietin.* J Neuroimaging, 2014. **24**(5): p. 504–8.
95. Santner, W., et al., *A longitudinal VBM study monitoring treatment with erythropoietin in patients with Friedreich ataxia.* Acta Radiol Short Rep, 2014. **3**(4): p. 2047981614531573.
96. Portaro, S., et al., *The role of robotic gait training and tDCS in Friedrich ataxia rehabilitation: A case report.* Medicine (Baltimore), 2019. **98**(8): p. e14447.
97. Meier, T., et al., *Assessment of neurological efficacy of idebenone in pediatric patients with Friedreich's ataxia: data from a 6-month controlled study followed by a 12-month open-label extension study.* J Neurol, 2012. **259**(2): p. 284–91.
98. Lynch, D.R., et al., *Efficacy of Omaveloxolone in Friedreich's Ataxia: Delayed-Start Analysis of the MOXIe Extension.* Mov Disord, 2023. **38**(2): p. 313–320.
99. Ivak, P., A. Zumrova, and I. Netuka, *Friedreich's ataxia and advanced heart failure: An ethical conundrum in decision-making.* J Heart Lung Transplant, 2016. **35**(9): p. 1144–5.
