# Supplementary material for: Key Interventions in Friedreich's Ataxia and Their Impact on Patient Outcomes: A Systematic Review
Source: Mov Disord. 2026 Jul 6;41(10):2564–91. doi: 10.1002/mds.70423 (PMC13686107; doi:10.1002/mds.70423)
Supplement: Supplementary file 2 [file MDS-41-2564-s002.docx]

| ICMJE DISCLOSURE FORM | |
| --- | --- |
| **Date:** | 1/7/2025 |
| **Your Name:** | Dr. Dorota Sarwinska |
| **Manuscript Title:** | Key Interventions in Friedreich’s Ataxia and their Impact on Patient Outcomes: A Systematic Review |
| **Manuscript Number (if known):** | MDS70423 |
| In the interest of transparency, we ask you to disclose all relationships/activities/interests listed below that are related to the content of your manuscript. “Related” means any relation with for-profit or not-for-profit third parties whose interests may be affected by the content of the manuscript. Disclosure represents a commitment to transparency and does not necessarily indicate a bias. If you are in doubt about whether to list a relationship/activity/interest, it is preferable that you do so.  The author’s relationships/activities/interests should be defined broadly. For example, if your manuscript pertains to the epidemiology of hypertension, you should declare all relationships with manufacturers of antihypertensive medication, even if that medication is not mentioned in the manuscript.  In item #1 below, report all support for the work reported in this manuscript without time limit. For all other items, the time frame for disclosure is the past 36 months. | |

|  | | | **Name all entities with whom you have this relationship or indicate none (add rows as needed)** | **Specifications/Comments (e.g., if payments were made to you or to your institution)** |
| --- | --- | --- | --- | --- |
| **Time frame: Since the initial planning of the work** | | | | |
| **1** | All support for the present manuscript (e.g., funding, provision of study materials, medical writing, article processing charges, etc.)  **No time limit for this item.** | | ☒ \| **None** | |
| **Time frame: past 36 months** | | | | |
| **2** | | Grants or contracts from any entity (if not indicated in item #1 above). | ☐ \| **None** Jennifer Faber was funded within the Advanced Clinician Scientist Programme (ACCENT, funded by the Federal Ministry of Research, Technology and Space (BMFTR) under the funding code (FKZ): 01EO2107) and as a PI of the iBehave Network, sponsored by the Ministry of Culture and Science of the State of North Rhine-Westphalia and received funding from the National Ataxia Foundation (NAF). JF is a member of the European Reference Network for Rare Neurological Diseases. \| Not related to this project; Marcus Grobe-Einsler received research support from the National Ataxia Foundation and Ataxia UK, and the European Joint Program for Rare Diseases (EJP‐RD) 2021 Transnational Call for Rare Disease Research Projects with local funding from the Bundesministerium für Bildung, Wissenschaft, Forschung und Technologie (Award Number 01GM2110). \| Not related to this project; Bernhard Michalowsky was funded by Gemeinsamer Bundesausschuss (G-BA), Bundesministerium für Bildung und Forschung (BMBF), Deutsche Demenzhilfe, and EuroQol. \| Not related to this project; Thomas Klockgether is receiving research support from the Bundesministerium für ­Forschung, Technologie und Raumfahrt (BMFTR), Servier, and UCB. \| Not related to this project | |
| **3** | | Royalties or licenses | ☒ \| **None** | |
| **4** | | Consulting fees | ☐ \| **None** Jennifer Faber received the consultancy honoraria from Vico therapeutics and Biogen. \| Not related to this project \| \|; Marcus Grobe-Einsler received honoraria from Biogen. \| Not related to this project \| \|; Bernhard Michalowsky received consultancy honoraria from Biogen, Lilly and EuroQol, \| Not related to this project \| \|; Thomas Klockgether has received within the last 24 months consulting fees from Arrowhead, Bristol-Myers Squibb, and UCB. \| Not related to this project \| \| | |
| **5** | | Payment or honoraria for lectures, presentations, speakers bureaus, manuscript writing or educational events | ☒ \| **None** | |
| **6** | | Payment for expert testimony | ☒ \| **None** | |
| **7** | | Support for attending meetings and/or travel | ☐ \| **None** Marcus Grobe-Einsler received travel support from Apario and the Ataxia Global initiative. \| Not related to this project | |
| **8** | | Patents planned, issued or pending | ☒ \| **None** | |
| **9** | | Participation on a Data Safety Monitoring Board or Advisory Board | ☒ \| **None** | |
| **10** | | Leadership or fiduciary role in other board, society, committee or advocacy group, paid or unpaid | ☒ \| **None** | |
| **11** | | Stock or stock options | ☒ \| **None** | |
| **12** | | Receipt of equipment, materials, drugs, medical writing, gifts or other services | ☒ \| **None** | |
| **13** | | Other financial or non-financial interests | ☒ \| **None** | |
|  | |  |  | |
| **Please place an “X” next to the following statement to indicate your agreement:** | | | | |
| ☒ | | I certify that I have answered every question and have not altered the wording of any of the questions on this form. | | |
